# Supplementary material for: BNP-Track: a framework for superresolved tracking
Source: Nat Methods. 2024 Jul 22;21(9):1716–24. doi: 10.1038/s41592-024-02349-9 (PMC11399105; doi:10.1038/s41592-024-02349-9)
Supplement: Supplementary file 1 — Supplementary Figs. 1–9, Tables 1–5, Discussion and Notes. [file 41592_2024_2349_MOESM1_ESM.pdf]

nature methods

Supplementary information https://doi.org/10.1038/s41592-024-02349-9

BNP-Track: a framework for superresolved tracking

In the format provided by the authors and unedited

Contents

A Supplementary Figures **2**

B Supplementary Tables **11**

C Supplementary Discussion **15**
C.1 Detailed comparison with TrackMate . . . 15
C.2 Robustness tests for parameter regimes . . . 16
C.3 Robustness tests for motion models . . . 17

D Supplementary Notes **18**
D.1 Probabilistic modeling . . . 18
D.1.1 Statistical notation . . . 18
D.1.2 Statistical inference . . . 19
D.1.3 Statistical simulation . . . 19
D.2 Frame of reference . . . 20
D.3 Time schedule . . . 21
D.4 Optics representation . . . 23
D.4.1 Point spread function . . . 23
D.4.2 Effective pixel function . . . 24
D.5 Dynamics representation . . . 25
D.6 Temporal discretization . . . 25
D.6.1 Simplification for time-independent emitter brightness and background photon flux . . 26
D.6.2 Emitter motion . . . 26
D.6.3 Extended BNP-Track for other motion models . . . 27
D.6.4 Extended BNP-Track to estimate multiple positions of one emitter within one frame . 28
D.7 Model likelihood . . . 30
D.8 Model priors . . . 31
D.8.1 Nonparametric prior distributions . . . 31
D.8.2 Parametric prior distributions . . . 31
D.9 Summary of model equations . . . 33
D.10 Evaluation and interpretation of the model posterior . . . 34
D.10.1 Evaluation . . . 34
D.10.2 Interpretation . . . 36
D.11 Calibration . . . 38
D.12 Camera read-out . . . 38
D.12.1 Point spread function and illumination profile . . . 38

References **40**

A Supplementary Figures

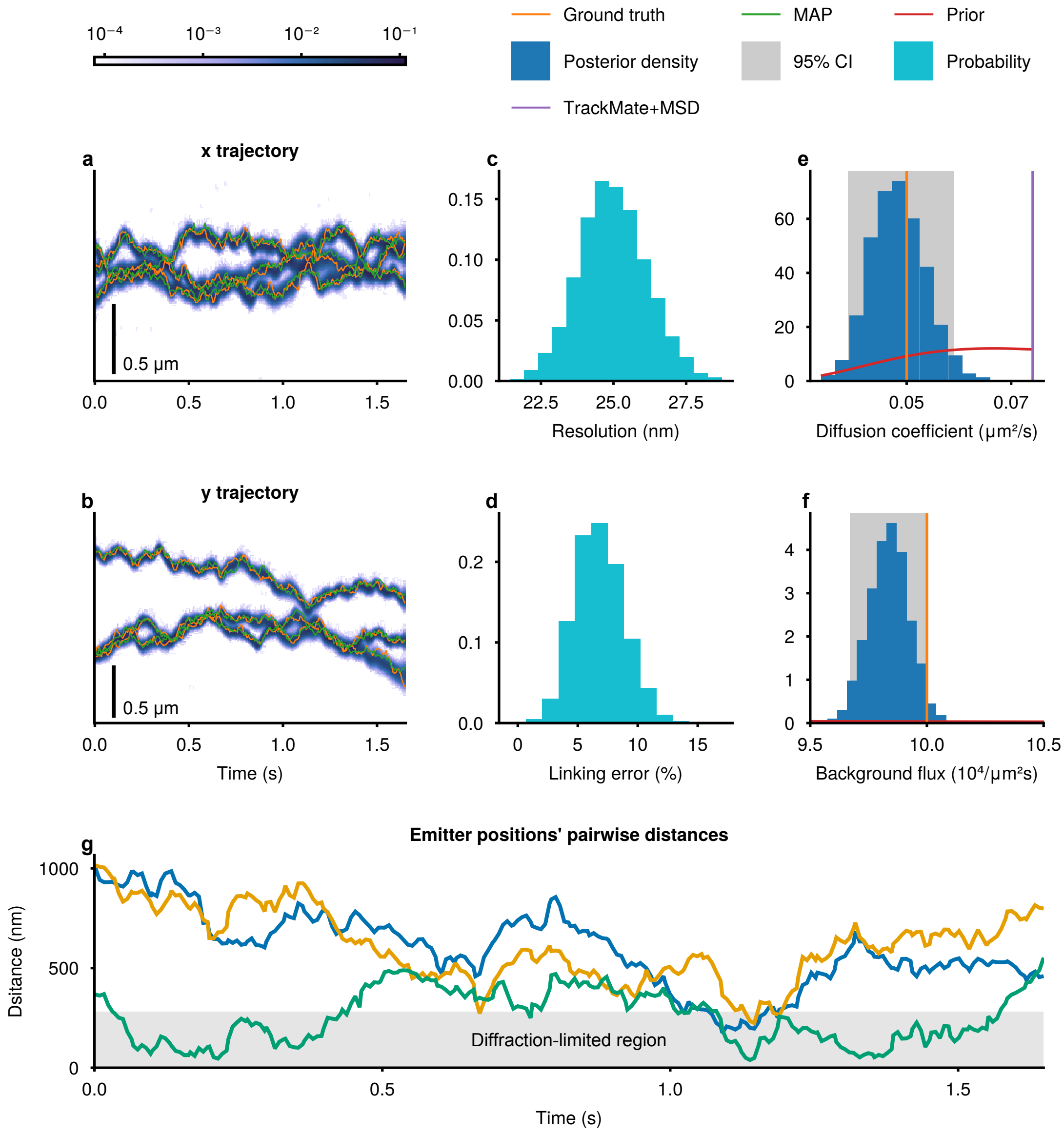

Figure A.1: Comprehensive tracking results obtained using BNP-Track for Supplementary Data 6. **a**–**b**, BNP-Track's tracking results for the x and y trajectories are displayed. Ground truth tracks are depicted in orange, while BNP-Track's MAP tracks are green. Shading denotes confidence (the probability of the emitter being in a 10 nm bin), with darker shades indicating higher confidence. **c**–**d**, Histograms illustrating BNP-Track's localization resolution and linking errors, respectively. **e**–**f**, BNP-Track's estimates of the diffusion coefficient and background flux are presented as posterior probability densities. Ground truth values are shown in orange, prior probability distributions in red, and the 95% CIs are depicted as shaded regions. **g**, The pairwise spatial separation between each emitter pair is plotted as a function of time, with the Rayleigh diffraction limit represented by the shaded region.

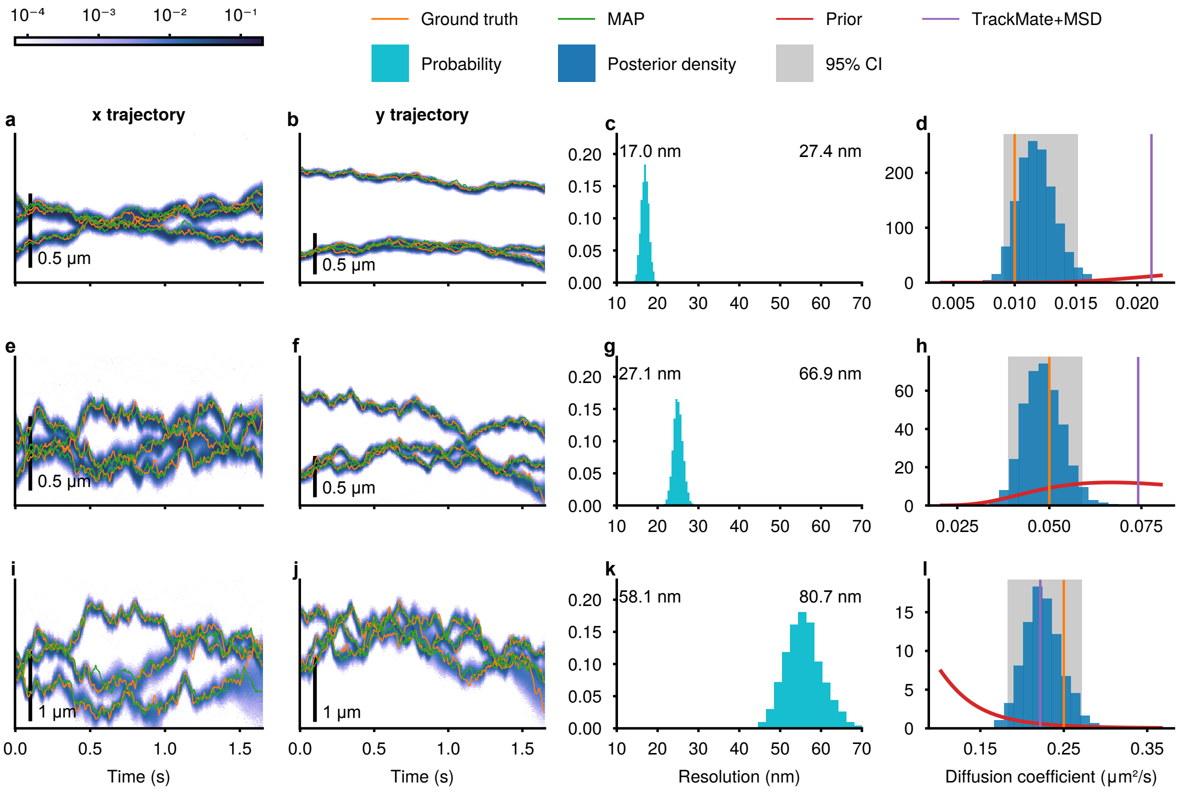

Figure A.2: Performance of BNP-Track under variable diffusion coefficients. **a–b**, Tracking results of BNP-Track for x and y trajectories, corresponding to Supplementary Data 7 with a diffusion coefficient of $0.01\,\mu m^2 s^{-1}$. The ground truth tracks are orange, while BNP-Track's MAP tracks are green. Shading indicates confidence (the probability of the emitter being in a 10 nm bin), with darker colors representing higher confidence. **c**, Histogram illustrating the localization resolution of BNP-Track, derived from the results in **a** and **b**. BNP-Track's MAP tracks' localization resolution and tracking error are displayed in the top-left and top-right, respectively. **d**, BNP-Track's estimate of the diffusion coefficient presented as its posterior probability density. The ground truth value is represented in orange, the prior probability distribution in red, and the 95% CI is depicted as the shaded region. **e–h**, same layout as **a–b** but for Supplementary Data 6 with a diffusion coefficient of $0.05\,\mu m^2 s^{-1}$. **i–l**, same layout as **a–b** but for Supplementary Data 8 with a diffusion coefficient of $0.25\,\mu m^2 s^{-1}$.

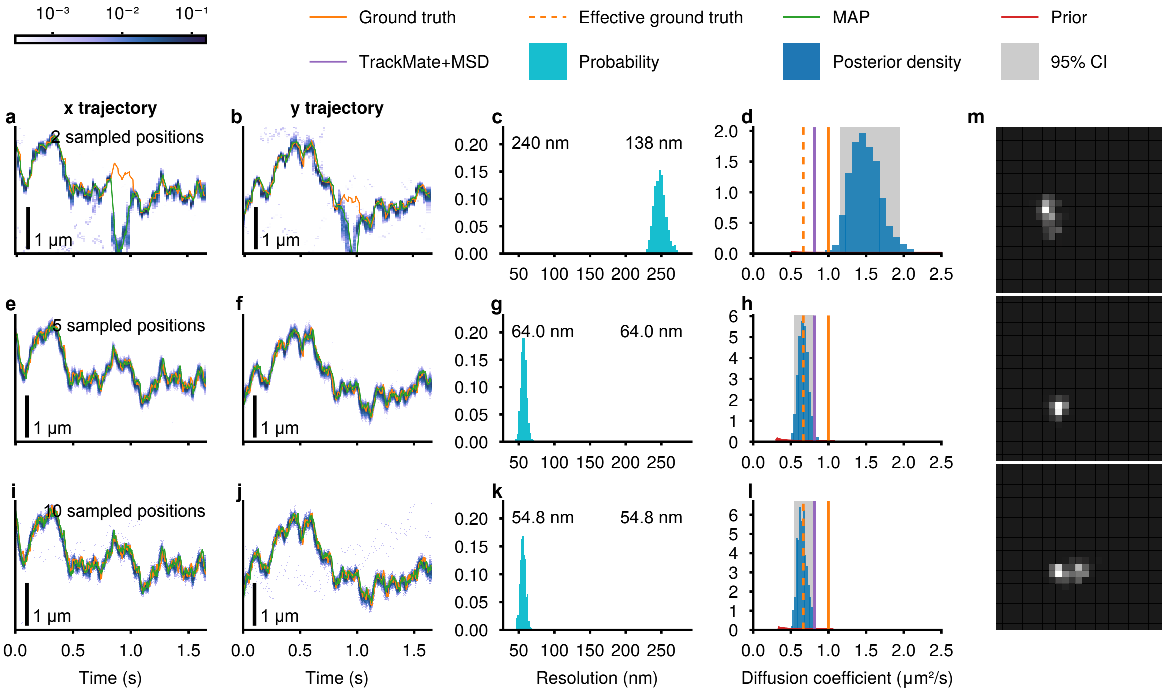

Figure A.3: BNP-Track's performance was evaluated across varying numbers of positions sampled within the exposure period of a single frame. The synthetic movie, presented in Supplementary Data 9, features a single emitter diffusing at $1\,\mu\mathrm{m}^2\mathrm{s}^{-1}$ only in the lateral plane. **a**–**b**, BNP-Track's tracking results with two positions sampled in each exposure period for the x and y trajectories are displayed. Ground truth tracks are depicted in orange, while BNP-Track's MAP tracks are green. Shading denotes confidence (the probability of the emitter being in a 10 nm bin), with darker shades indicating higher confidence. **c**, Histogram illustrating BNP-Track's localization resolution. The localization resolution and tracking error of BNP-Track's MAP track are presented in the top-left and top-right, respectively. **d**, BNP-Track's estimate of the diffusion coefficient is presented as a posterior probability density. The ground truth value is depicted as a solid orange line, the prior probability distribution in red, and the 95% CI shaded. The dashed orange lines represent the effective ground truth diffusion coefficient, $0.67\,\mu\mathrm{m}^2\mathrm{s}^{-1}$. **e**–**h**, Same layout as **a**–**d**, with Supplementary Data 9 analyzed using five positions sampled in each exposure period. **i**–**l**, Same layout as **a**–**d**, with Supplementary Data 9 analyzed using five positions sampled in each exposure period. **m**, Frame 24, 25, and 26 in Supplementary Data 9 (noise removed for illustration) show highly aberrated PSFs due to motion blur.

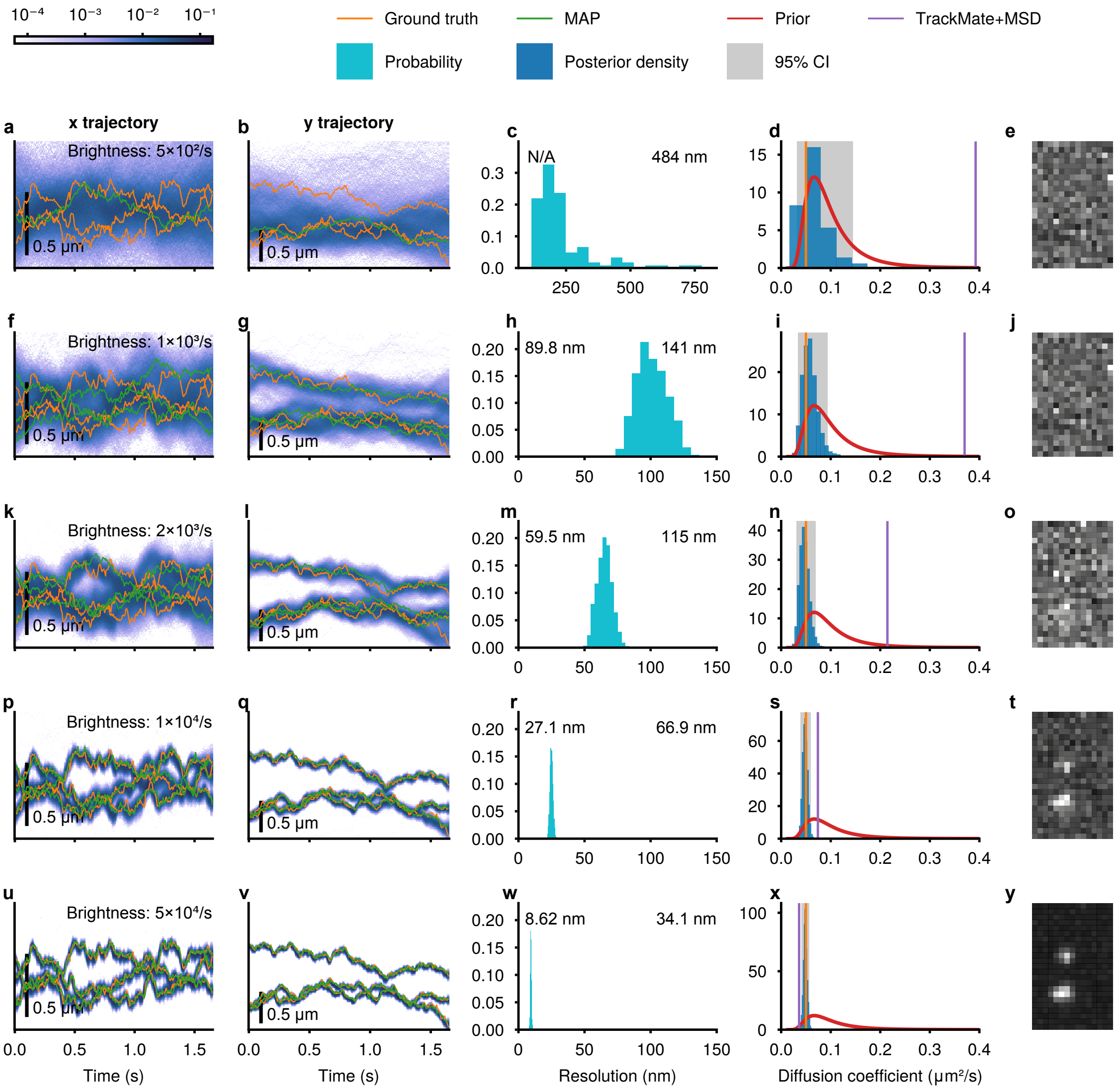

Figure A.4: Performance of BNP-Track across various levels of emitter brightness. See appendix D.4 for details on how we define emitter brightness. All datasets here share a common set of ground truth tracks. **a**–**b**, Tracking results of BNP-Track for Supplementary Data 10 ($5 \times 10^2\,\mathrm{s}^{-1}$ brightness) are presented for the x and y trajectories. Ground truth tracks are orange, while BNP-Track's MAP tracks are green. Shading indicates confidence (the probability of the emitter being in a 10 nm bin), with darker shades signifying higher confidence. **c**, Histogram illustrating BNP-Track's localization resolution. The localization resolution and tracking error of BNP-Track's MAP track are presented in the top-left and top-right corners, respectively. **d**, BNP-Track's estimate of the diffusion coefficient is presented as a posterior probability density. The ground truth value is depicted as a solid orange line, the prior probability distribution in red, and the 95% CI shaded. **e**, Frame 2 of the movie analyzed. **f**–**j**, **k**–**o**, **p**–**t**, **u**–**y**, Similar layout as **a**–**e**, but for Supplementary Data 11 ($1 \times 10^3\,\mathrm{s}^{-1}$ brightness), 12 ($2 \times 10^3\,\mathrm{s}^{-1}$ brightness), 6 ($1 \times 10^4\,\mathrm{s}^{-1}$ brightness), and 13 ($5 \times 10^4\,\mathrm{s}^{-1}$ brightness), respectively.

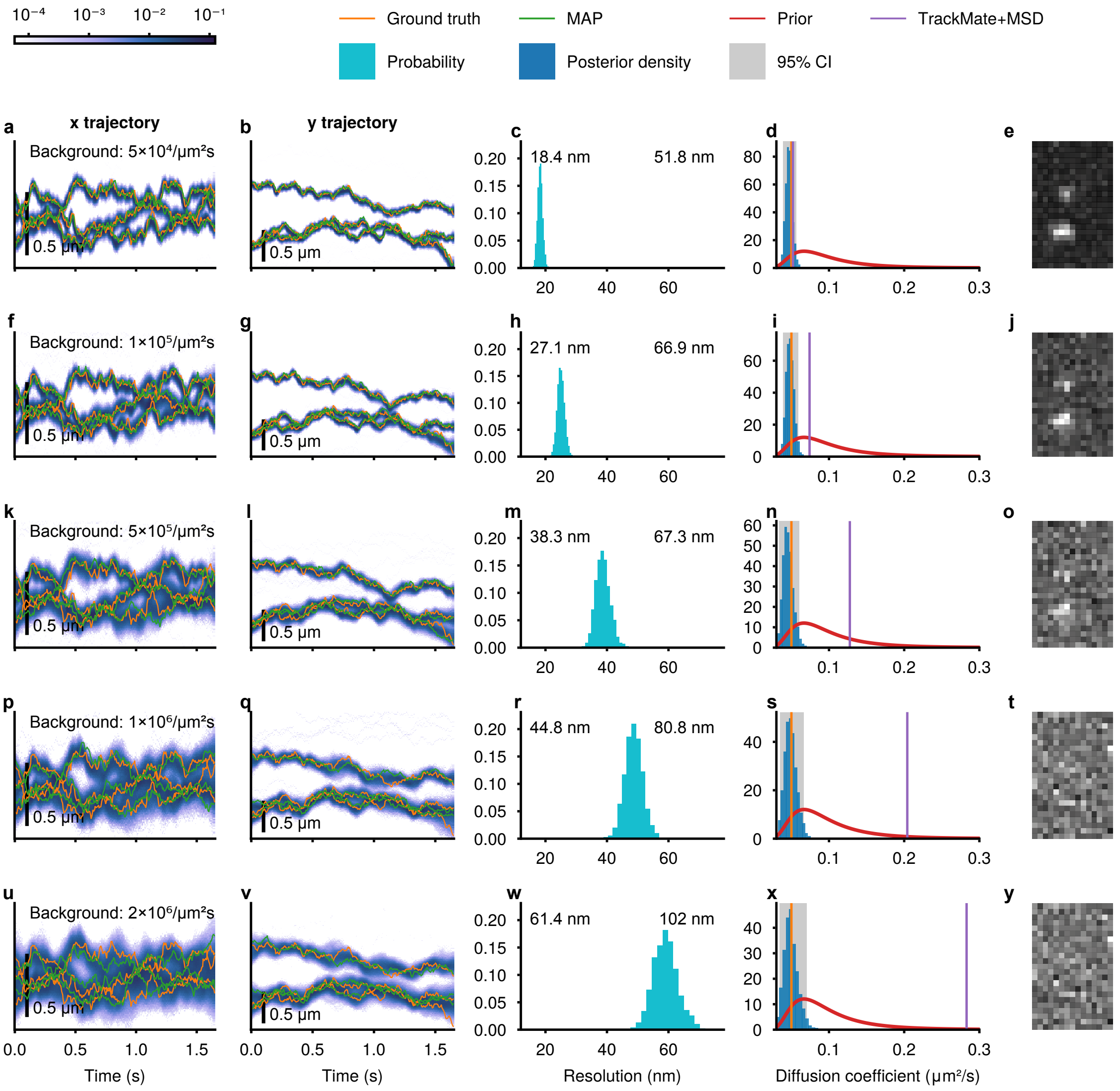

Figure A.5: Performance of BNP-Track across various levels of background photon flux. See appendix D.4 for details on how we define background flux. All datasets here share a common set of ground truth tracks. **a**–**b**, Tracking results of BNP-Track for Supplementary Data 14 ($5 \times 10^4\ \mu m^{-2}s^{-1}$ background) are presented for the x and y trajectories. Ground truth tracks are orange, while BNP-Track's MAP tracks are green. Shading indicates confidence (the probability of the emitter being in a 10 nm bin), with darker shades signifying higher confidence. **c**, Histogram illustrating BNP-Track's localization resolution. The localization resolution and tracking error of BNP-Track's MAP track are presented in the top-left and top-right corners, respectively. **d**, BNP-Track's estimate of the diffusion coefficient is presented as a posterior probability density. The ground truth value is depicted as a solid orange line, the prior probability distribution in red, and the 95% CI shaded. **e**, Frame 2 of the movie analyzed. **f**–**j**, **k**–**o**, **p**–**t**, **u**–**y**, Similar layout as **a**–**e**, but for Supplementary Data 6 ($1 \times 10^5\ \mu m^{-2}s^{-1}$ background), 15 ($5 \times 10^5\ \mu m^{-2}s^{-1}$ background), 16 ($1 \times 10^6\ \mu m^{-2}s^{-1}$ background), and 17 ($2 \times 10^6\ \mu m^{-2}s^{-1}$ background), respectively.

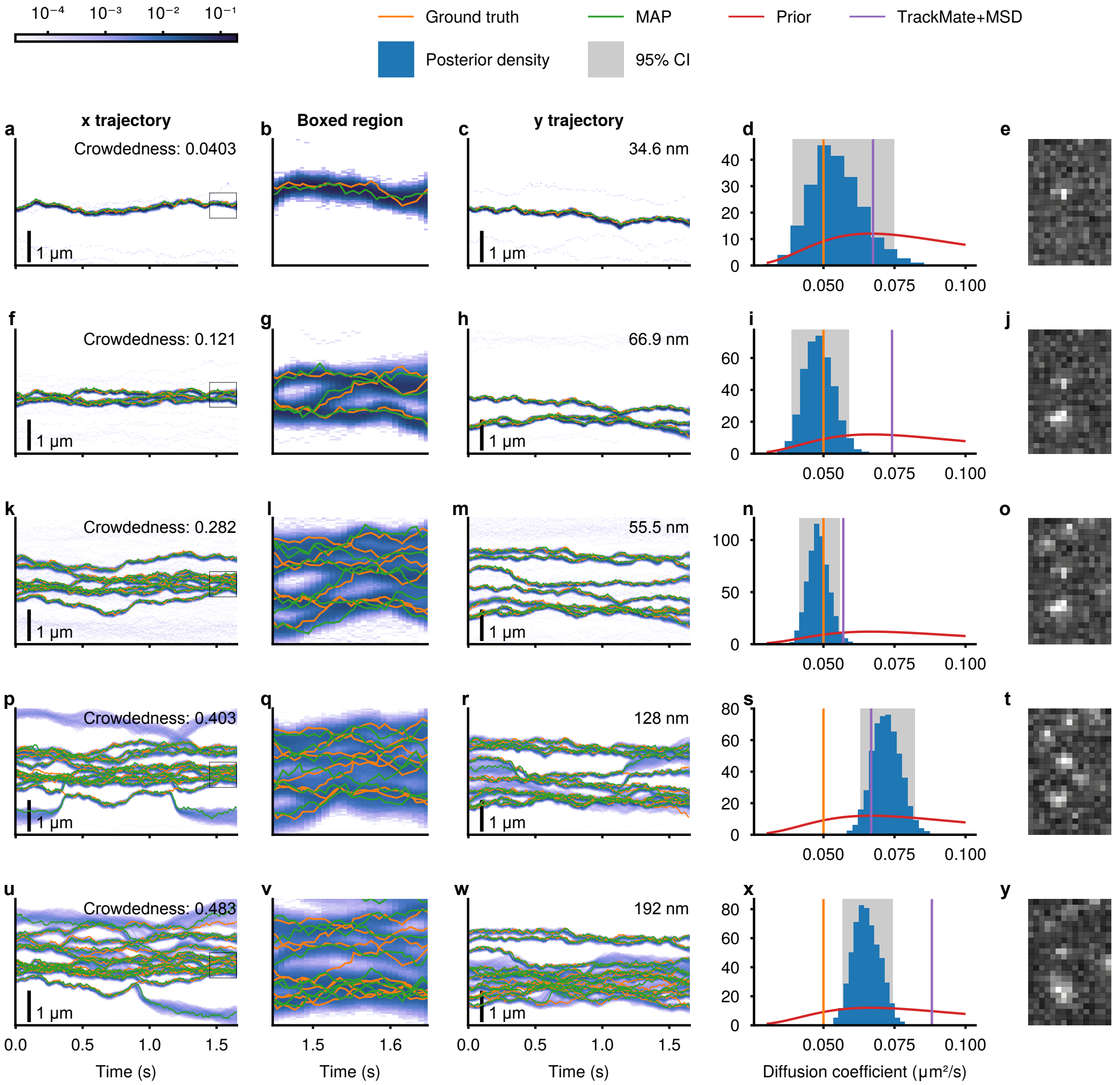

Figure A.6: BNP-Track's performance under for various numbers of emitters in the fixed field of view. **a**, Tracking results of BNP-Track for Supplementary Data 5 with one emitter are presented for the x trajectory. Ground truth tracks are orange, while BNP-Track's MAP tracks are green. Shading indicates confidence (the probability of the emitter being in a 10 nm bin), with darker shades signifying higher confidence. Crowdedness (see main text equation 4) is presented in the top-right corner. **b**, Zoomed-in view for the boxed region in **a**. **c**, Tracking results of BNP-Track for the y trajectory. The tracking error of BNP-Track's MAP track is presented in the top-right corner. **d**, BNP-Track's estimate of the diffusion coefficient is presented as a posterior probability density. The ground truth value is depicted as a solid orange line, the prior probability distribution in red, and the 95% CI shaded. **e**, Frame 2 of the movie analyzed. **f**–**j**, **k**–**o**, **p**–**t**, **u**–**y**, Similar layout as **a**–**e**, but for Supplementary Data 6 (three emitters), 24 (seven emitters), 25 (ten emitters), and 26 (twelve emitters), respectively.

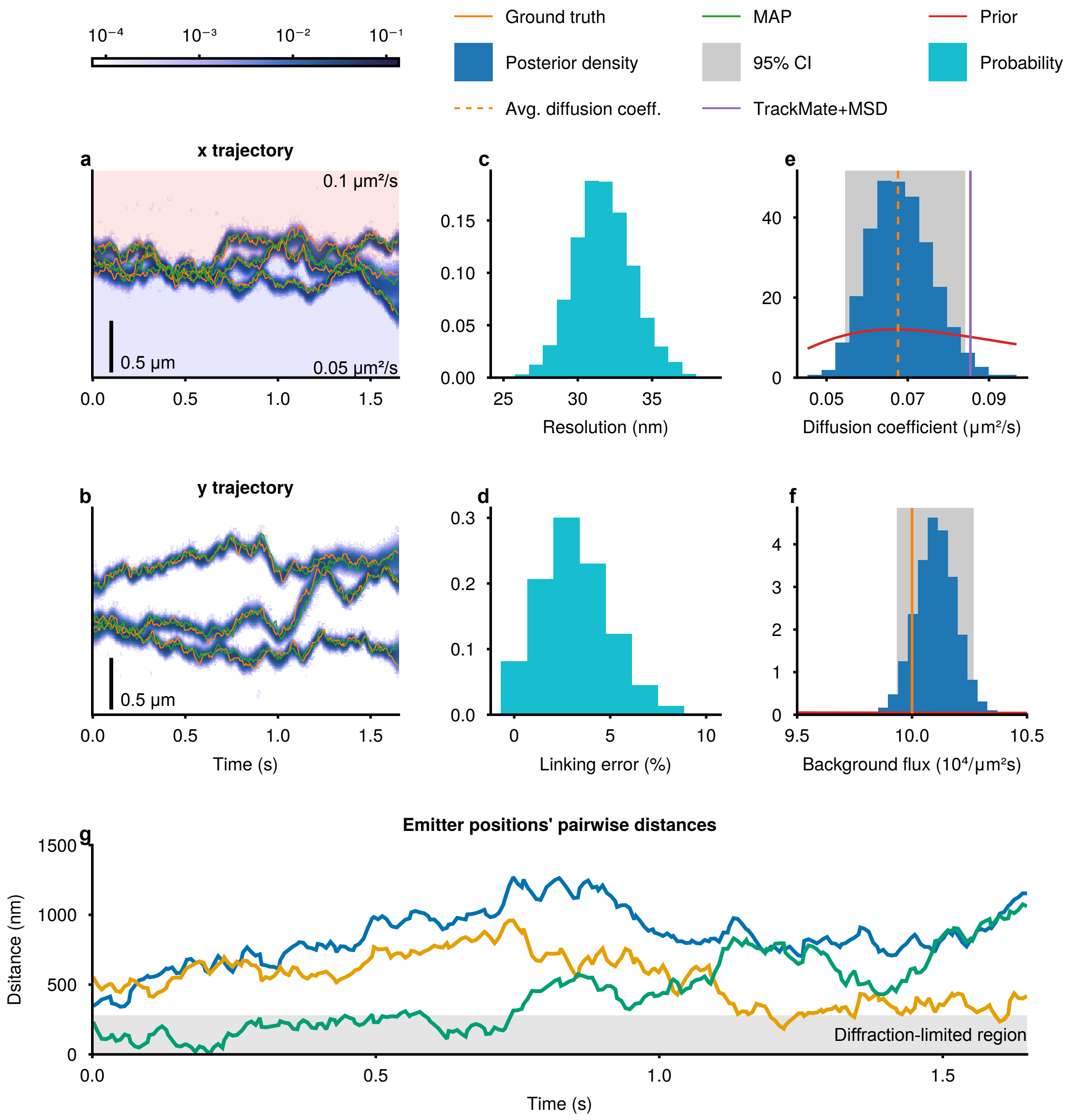

Figure A.7: Tracking results for Supplementary Data 27 using BNP-Track. The layout of this figure mirrors that of Fig. A.1. Shading indicates confidence (the probability of the emitter being in a 10 nm bin), with darker shades signifying higher confidence. Furthermore, the shaded areas in **a** indicate the spatial variation of the diffusion coefficient. The diffusion coefficient values are presented in the respective regions. In **e**, the dashed orange line represents the average ground truth diffusion coefficient, 0.068 $\mu m^2 s^{-1}$, and the purple line represents the diffusion coefficient estimated by TrackMate with MSD, 0.085 $\mu m^2 s^{-1}$.

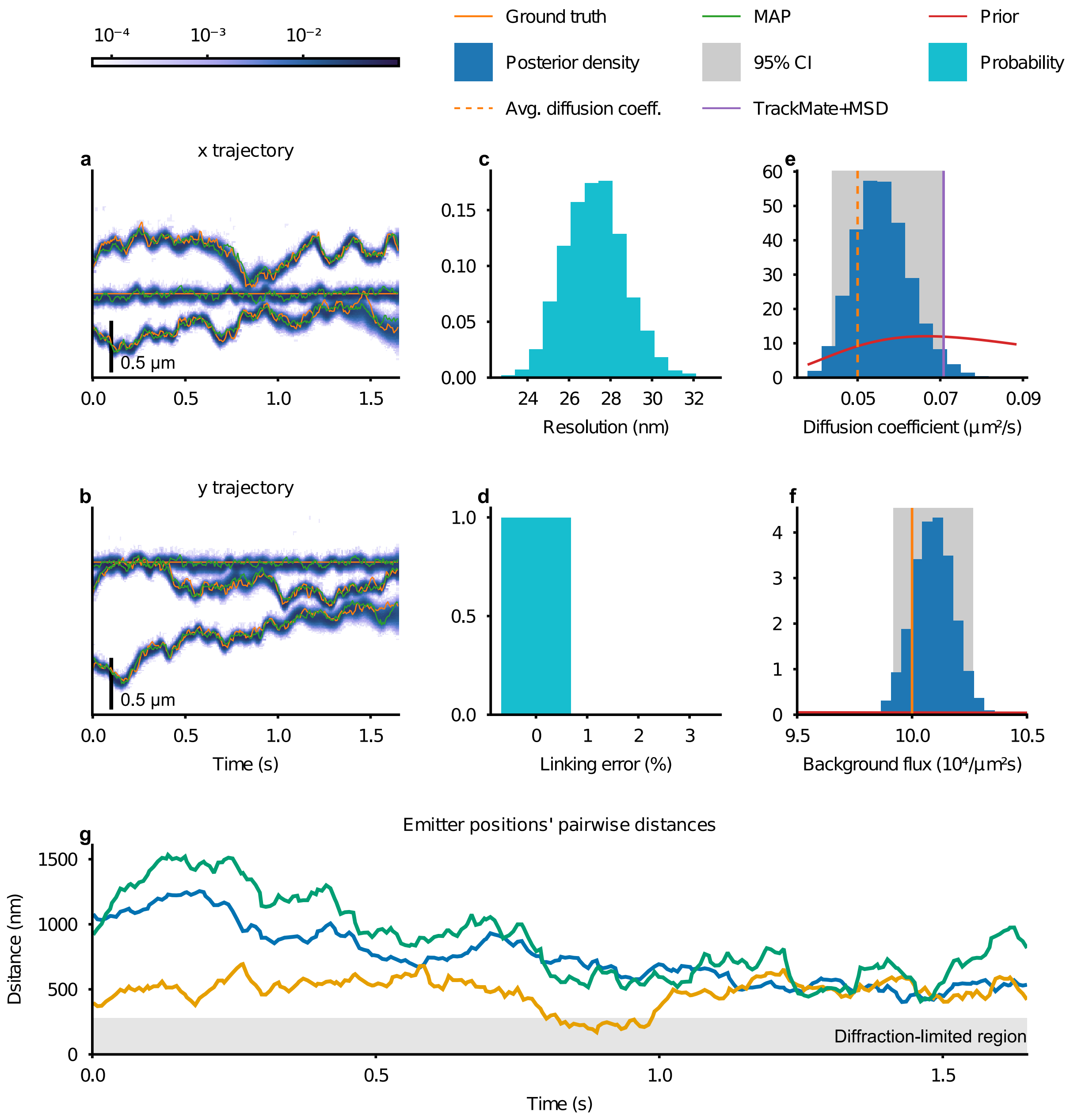

Figure A.8: Tracking results for Supplementary Data 28 using BNP-Track. The three emitters have ground truth diffusion coefficients $0\,\mu\text{m}^2\text{s}^{-1}$, $0.05\,\mu\text{m}^2\text{s}^{-1}$, and $0.1\,\mu\text{m}^2\text{s}^{-1}$, respectively. The layout of this figure mirrors that of Fig. A.1. Shading indicates confidence (the probability of the emitter being in a 10 nm bin), with darker shades signifying higher confidence. Furthermore, in **e**, the dashed orange line represents the average ground truth diffusion coefficient, $0.05\,\mu\text{m}^2\text{s}^{-1}$, and the purple line represents the diffusion coefficient estimated by TrackMate with MSD, $0.071\,\mu\text{m}^2\text{s}^{-1}$.

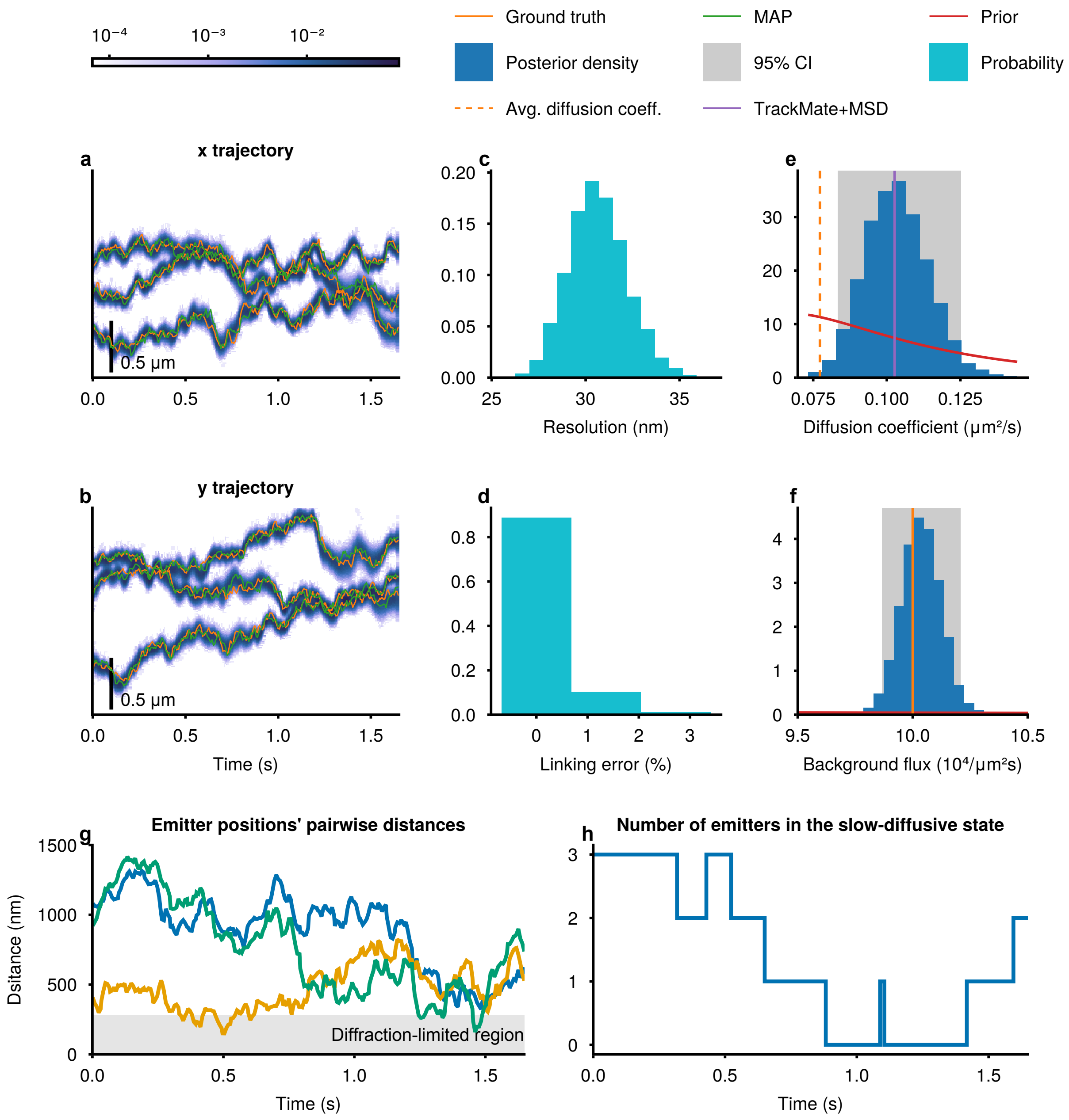

Figure A.9: Tracking results for Supplementary Data 29 using BNP-Track. Each emitter can undergo independent transitions between a slow-diffusive state ($0.05\,\mu\mathrm{m}^2\mathrm{s}^{-1}$) and a fast-diffusive state ($0.1\,\mu\mathrm{m}^2\mathrm{s}^{-1}$). The layout of this figure mirrors that of Fig. A.1. Shading indicates confidence (the probability of the emitter being in a 10 nm bin), with darker shades signifying higher confidence. Furthermore, in **e**, the dashed orange line represents the average ground truth diffusion coefficient, $0.077\,\mu\mathrm{m}^2\mathrm{s}^{-1}$, and the purple line represents the diffusion coefficient estimated by TrackMate with MSD, $0.10\,\mu\mathrm{m}^2\mathrm{s}^{-1}$. In **h**, the number of emitters in the slow-diffusive state is plotted as a function of time.

B Supplementary Tables

Description	Symbol	Unit	Value
Background flux	C	$\mu m^{-2}s^{-1}$	10^5
Emission wavelength	λ	nm	665
Emitter brightness	h	s^{-1}	10^4
Exposure period	τ	s	0.03
Frame period		s	0.033
Frame height		pixel	23
Frame with		pixel	15
Numerical aperture	NA		1.45
Pixel size		nm	133
Refractive index	n_{RI}		1.515
Noise excess factor	f		2

Table B.1: List of parameter values used in data simulation.

	BNP-Track MAP	TrackMate	u-track	BNP-Track MAP	TrackMate	u-track	BNP-Track MAP	TrackMate	u-track
Gate value (ϵ, pixel)		2			5			10	
**Global measures**									
Pairing distance (pixel)	13.0	13.3	12.1	13.0	13.3	12.1	13.0	13.3	12.1
Normalized pairing score (α)	0.867	0.867	0.879	0.948	0.947	0.952	0.974	0.973	0.976
Full normalized score (β)	0.867	0.867	0.879	0.948	0.947	0.952	0.974	0.973	0.976
**Tracks**									
Number of reference tracks	1	1	1	1	1	1	1	1	1
Number of candidate tracks	1	1	1	1	1	1	1	1	1
Similarity between tracks	1.0	1.0	1.0	1.0	1.0	1.0	1.0	1.0	1.0
Number of paired tracks	1	1	1	1	1	1	1	1	1
Number of missed tracks	0	0	0	0	0	0	0	0	0
Number of spurious tracks	0	0	0	0	0	0	0	0	0
**Detections**									
Number of reference detections	50	50	50	50	50	50	50	50	50
Number of candidate detections	50	50	50	50	50	50	50	50	50
Similarity between detections	1.0	1.0	1.0	1.0	1.0	1.0	1.0	1.0	1.0
Number of paired detections	50	50	50	50	50	50	50	50	50
Number of missed detections	0	0	0	0	0	0	0	0	0
Number of spurious detections	0	0	0	0	0	0	0	0	0
**Detection accuracy (pixel)**									
Root mean-square error	0.300	0.314	0.297	0.300	0.314	0.297	0.300	0.314	0.297
Minimum distance	0.0310	0.0168	0.0241	0.0310	0.0168	0.0241	0.0310	0.0168	0.0241
Maximum distance	0.660	0.809	0.888	0.660	0.809	0.888	0.660	0.809	0.888
Distance standard deviation	0.150	0.166	0.173	0.150	0.166	0.173	0.150	0.166	0.173

Table B.2: A comprehensive comparison of tracking performance, generated using the Tracking Performance Measures plugin in Icy [1]. The comparison involves tracks from BNP-Track's MAP, TrackMate, and u-track, focusing on the one-emitter movie presented in Supplementary Data 6, as depicted in Fig. 3a–c. Details on how these tracks were obtained can be found in the main text. The evaluation was performed three times with varying gate values. For an in-depth explanation of each metric, please refer to Ref. [2].

	BNP-Track MAP	Track-Mate A	Track-Mate B	BNP-Track MAP	Track-Mate A	Track-Mate B	BNP-Track MAP	Track-Mate A	Track-Mate B
Gate value (ϵ, pixel)		2			5			10	
**Global measures**									
Pairing distance (pixel)	69.9	154	183	75.4	337	386	75.4	642	672
Normalized pairing score (α)	0.767	0.486	0.389	0.899	0.550	0.484	0.950	0.572	0.552
Full normalized score (β)	0.767	0.486	0.389	0.899	0.550	0.484	0.950	0.572	0.552
**Tracks**									
Number of reference tracks	3	3	3	3	3	3	3	3	3
Number of candidate tracks	3	3	3	3	3	3	3	3	3
Similarity between tracks	1.0	1.0	1.0	1.0	1.0	1.0	1.0	1.0	1.0
Number of paired tracks	3	3	3	3	3	3	3	3	3
Number of missed tracks	0	0	0	0	0	0	0	0	0
Number of spurious tracks	0	0	0	0	0	0	0	0	0
**Detections**									
Number of reference detections	150	150	150	150	150	150	150	150	150
Number of candidate detections	150	106	93	150	106	93	150	106	93
Similarity between detections	0.899	0.583	0.455	1.00	0.593	0.620	1.00	0.593	0.620
Number of paired detections	142	88	76	150	89	93	150	89	93
Number of missed detections	8	62	74	0	61	57	0	61	57
Number of spurious detections	8	1	17	0	0	0	0	0	0
**Detection accuracy (pixel)**									
Root mean-square error	0.518	0.424	0.596	0.807	0.473	1.77	0.807	0.473	1.77
Minimum distance	0.0441	0.0242	0.0227	0.0441	0.0242	0.0227	0.0441	0.0242	0.0227
Maximum distance	1.90	1.07	1.96	3.62	1.07	4.65	3.62	6.19	4.65
Distance standard deviation	0.353	0.247	0.371	0.631	0.303	1.40	0.631	0.303	1.40

Table B.3: A comprehensive comparison of tracking performance, generated using the Tracking Performance Measures plugin in Icy [1]. The comparison involves tracks from BNP-Track's MAP and TrackMate, focusing on the three-emitter movie presented in Supplementary Data 7, as depicted in Fig. 3d–f. Details on how these tracks were obtained can be found in the main text. The evaluation was performed three times with varying gate values. For an in-depth explanation of each metric, please refer to Ref. [2].

Supplementary Data	18		19		20		21		22		23	
Corresponding panel in Fig. 5	**b**	**c**	**e**	**f**	**h**	**i**	**k**	**l**	**n**	**o**	**q**	**r**
**Global measures**												
Pairing distance (pixel)	10.9	74.1	8.67	110	12.1	111	12.2	122	12.0	118	16.47	121
Normalized pairing score (α)	0.950	0.663	0.961	0.501	0.945	0.498	0.945	0.443	0.946	0.464	0.925	0.452
Full normalized score (β)	0.950	0.663	0.961	0.501	0.945	0.498	0.945	0.443	0.946	0.464	0.925	0.452
**Tracks**												
Number of reference tracks	2	2	2	2	2	2	2	2	2	2	2	2
Number of candidate tracks	2	2	2	2	2	2	2	1	2	2	2	1
Similarity between tracks	1.0	1.0	1.0	1.0	1.0	1.0	1.0	0.5	1.0	1.0	1.0	0.5
Number of paired tracks	2	2	2	2	2	2	2	1	2	2	2	1
Number of missed tracks	0	0	0	0	0	0	0	1	0	0	0	1
Number of spurious tracks	0	0	0	0	0	0	0	0	0	0	0	0
**Detections**												
Number of reference detections	44	44	44	44	44	44	44	44	44	44	44	44
Number of candidate detections	44	38	44	31	44	27	44	22	44	23	44	22
Similarity between detections	1.00	0.795	1.00	0.659	1.00	0.614	1.00	0.500	1.00	0.523	1.00	0.500
Number of paired detections	44	35	44	29	44	27	44	22	44	23	44	22
Number of missed detections	0	9	0	15	0	17	0	22	0	21	0	22
Number of spurious detections	0	0	0	0	0	0	0	0	0	0	0	0
**Detection accuracy (pixel)**												
Root mean-square error	0.277	1.13	0.221	1.64	0.302	1.05	0.309	0.694	0.300	0.633	0.420	0.576
Minimum distance	0.0275	0.101	0.0439	0.103	0.0353	0.290	0.0195	0.0366	0.0599	0.0254	0.0803	0.0620
Maximum distance	0.511	3.54	0.495	3.73	0.508	2.09	0.664	1.91	0.566	1.15	1.09	1.16
Distance standard deviation	0.122	0.767	0.101	1.12	0.125	0.454	0.136	0.401	0.127	0.293	0.191	0.321

Table B.4: Comprehensive comparison of tracking performance for the synthetic movies illustrated in Fig. 4. Each column in the table corresponds to specific panels.

Probability distribution	Variable	Values	Probability density
Normal(μ, v)	x	from $-\infty$ to $+\infty$	$\frac{1}{\sqrt{2\pi v}} \exp\left(-\frac{(\mu-x)^2}{2v}\right)$
Gamma(ϕ, β)	w	from 0 to ∞	$\frac{1}{\beta\Gamma(\phi)} \left(\frac{w}{\beta}\right)^{\phi-1} \exp\left(-\frac{w}{\beta}\right)$
InvGamma(ϕ, β)	d	from 0 to ∞	$\frac{1}{\beta\Gamma(\phi)} \left(\frac{\beta}{d}\right)^{\phi+1} \exp\left(-\frac{\beta}{d}\right)$
Uniform$_{[x,y]}$	z	from x to y	$\frac{1}{y-x}$
Beta(α, β)	q	from 0 to 1	$\frac{1}{\mathrm{B}(\alpha,\beta)} q^{\alpha-1}(1-q)^{\beta-1}$
Bernoulli(q)	b	0 or 1	$q\delta_1(b) + (1-q)\delta_0(b)$
Binomial(m, r)	b	0 or 1 or 2 or ...	$\sum_{k=0}^{\infty} \frac{m!}{k!(m-k)!} r^k (1-r)^{m-k} \delta_k(b)$
Poisson(γ)	b	0 or 1 or 2 or ...	$\sum_{k=0}^{\infty} \frac{\gamma^k}{k!} e^{-\gamma} \delta_k(b)$
Uniform$_{1:N}$	k	from 1 to N	$\frac{1}{N}$

Table B.5: Summary of probability distributions. In this table $\Gamma(\cdot)$ and $\mathrm{B}(\cdot,\cdot)$ denote the Gamma and Beta functions, respectively [3]. Further, $x!$ denotes the factorial of x and $\delta_x(\cdot)$ denotes the Dirac delta centered at x.

C Supplementary Discussion

C.1 Detailed comparison with TrackMate

We start with the simplest possible case of a single emitter in the FOV. In this case, both BNP-Track (Fig. 3a) and TrackMate (Fig. 3b) successfully track the single emitter throughout the movie with similar resolution (34.6 nm vs. 35.4 nm). To further demonstrate that conventional SPT tools typically perform well in this simple scenario, we also include the track obtained with u-track [4] using the point source particle detection process (Fig. 3c), which again achieves a similar resolution at 32.1 nm. We note that in Figs. 3a to 3c, the resolution is equal to the tracking error, since there are no missing segments or incorrect links in the tracks.

Having established that BNP-Track and TrackMate work well for the simple case, we move on to a more complicated dataset with three emitters. Predictably, as we begin encountering PSF overlap in multiple emitters for the three-emitter dataset (see Fig. A.1g and Supplementary Data 6), the performances of BNP-Track and TrackMate diverge (we note that u-track's performance is not shown here because the current FOV is too small for running its algorithm). While BNP-Track remains capable of tracking all three emitters throughout the entire movie with a resolution of 27.1 nm from the MAP tracks, even as these fall below the diffraction limit in time intervals 0.03 s to 0.4 s and 1.1 s to 1.5 s (see Fig. A.1a), several issues arise for the TrackMate tracks (Figs. 3e and 3f). For instance, diffraction-limited emitters get interpreted as one emitter (solid boxes), incorrect links with large jumps (dashed boxes), and spurious detections (the dotted box) from TrackMate Set B. These issues indicate that TrackMate can no longer resolve the emitters in this dataset; hence, no resolution is calculated. As for the tracking error between the ground truth tracks and each SPT method's output, BNP-Track's MAP tracks have a tracking error of 66.9 nm, while both TrackMate track sets yield tracking errors of no less than 300 nm.

These benchmarks highlight BNP-Track's superior tracking performance. For instance, as illustrated by the dashed boxes in Figs. 3e and 3f, TrackMate tracks exhibit mislinks with sizes that exceed the nominal diffraction limit (approximately half of the scale bar). Consequently, estimating the diffusion coefficient through the mean squared displacement (MSD) using these tracks results in approximately $0.074\,\mu\text{m}^2\text{s}^{-1}$ and $0.11\,\mu\text{m}^2\text{s}^{-1}$, respectively. In comparison to the ground truth value of $0.05\,\mu\text{m}^2\text{s}^{-1}$, these values correspond to an overestimation of roughly 50% and 100%, respectively.

In contrast, BNP-Track reports the diffusion coefficient's 95% CI to be $0.039\,\mu\text{m}^2\text{s}^{-1}$ to $0.059\,\mu\text{m}^2\text{s}^{-1}$ with both mean and median at $0.048\,\mu\text{m}^2\text{s}^{-1}$. This underscores the substantial impact of BNP-Track's tracking accuracy on the reliability of research findings.

Three additional points are furthermore in order: (i) As illustrated in Fig. 3d, BNP-Track's tracking error is larger than its resolution, which is due to mislinks. However, we posit that this is not a major concern since it only occurs near 1.1 s to 1.2 s (frame 35) where two emitters are laterally less than 40 nm apart (see Fig. A.1a) while one of them is out-of-focus (300 nm away from the in-focus plane). Under this circumstance, these two emitters are very difficult to distinguish. (ii) Even though the three-emitter dataset contains the same track as the one-emitter dataset, BNP-Track actually achieves a better resolution in the more complicated three-emitter dataset. We attribute this finding to the fact that BNP-Track leverages all spatiotemporal correlations, as detailed in the next section, to localize more out-of-focus emitters. (iii) TrackMate Set B does a little better than Set A in estimating the emitter numbers by increasing the overall tracking error.

More quantitative comparisons generated by the Tacking Performance Measures plugin [2] in Icy [1] are provided in tables B.2 and B.3.

C.2 Robustness tests for parameter regimes

Here, we test the robustness of BNP-Track regarding several quantities of interest: diffusion coefficient, emitter brightness, background photon flux, and the spatial separation between emitters. BNP-Track remains robust in analyzing image sequences generated with different diffusion coefficients, as shown in Fig. A.2. Here, BNP-Track accurately tracks all emitters, as determined by localization resolution, and determines the correct diffusion coefficient, even when the diffusion coefficient changes by a factor of 25 from the slowest to the fastest emitter. As observed in Fig. A.2, the distribution of emitter positions becomes broader as the diffusion coefficient increases. This is supported by the 95% CIs of the localization resolutions, which range from (15.2 to 18.8) nm in Figs. A.2a to A.2d, to (22.8 to 27.3) nm in Figs. A.2e to A.2h, to (47.8 to 65.4) nm in Figs. A.2i to A.2l. One major factor contributing to this trend is the motion blur introduced by increasing the diffusion coefficient. Another important factor is that faster diffusing species have less time to remain within the FOV or move away from the in-focus plane, resulting in fewer informative frames (frames with sufficient detected photons).

As a general framework, BNP-Track can also be extended to incorporate faster-diffusing species when needed. Similarly to the approach taken in Ref. [5], we estimate multiple positions for each emitter within each camera exposure period. See appendix D.6.4 for a mathematical explanation and, in Fig. A.3, we test this extension using a synthetic dataset, Supplementary Data 9, consisting of an emitter diffusing at $1\,\mu\text{m}^2\text{s}^{-1}$ with camera exposure being 30 ms, where motion blur creates significant PSF aberrations, see Fig. A.3m.

In addition to assessing the robustness of BNP-Track with various diffusion coefficients, the performance of a particle tracking tool under different noise levels holds significant interest. To investigate this, we created Supplementary Data 10–13, maintaining fixed parameters except for varying the emitter brightness within the range of $5 \times 10^2\,\text{s}^{-1}$ to $5 \times 10^4\,\text{s}^{-1}$, and Supplementary Data 14–17, where the background photon flux is adjusted only within the range of $5 \times 10^4\,\mu\text{m}^{-2}\text{s}^{-1}$ to $2 \times 10^6\,\mu\text{m}^{-2}\text{s}^{-1}$ (refer to appendix D.4 for the

interpretation of these values). The corresponding outcomes are illustrated in Figs. A.4 and A.5. Throughout most of these assessments, BNP-Track consistently accurately estimates emitter numbers, yielding a localization resolution twice as small as the nominal diffraction limit. The sole exception arises when the emitter brightness equals $5 \times 10^2\,\mathrm{s}^{-1}$. Nevertheless, as depicted in Fig. A.4e, the human eye would fail to detect any emitter in this scenario, indicating that this failure occurs in a very low brightness regime.

Next, in Fig. 4, we test how closely two emitters can come together while retaining the ability of BNP-Track to enumerate the number of emitters and track them. To this end, a pair of estimated tracks from ROI-2 of Fig. 2 were used as the ground truth for the simulation of synthetic data (shown in the top row of Fig. 4) using the same parameters. Then, the mean displacement between emitters is gradually decreased. The middle row of Fig. 4 shows reconstructed tracks and comparisons with the ground truth. Remarkably, the tracking error remains $\approx$40 nm in 2D and increases slightly (by $\approx$50 nm) in 3D throughout synthetic scenarios, below the diffraction limit.

As seen in Figs. 3e and 3f, existing tracking methods, such as TrackMate, can fail to resolve emitters with separations close to or below the diffraction limit. To further demonstrate this point, we show the results from TrackMate Set B (which better estimates emitter numbers). When the mean displacement between emitters remains well above the lateral diffraction limit (the first three columns of Fig. 4), TrackMate Set B can be tuned to resolve two emitters, albeit with missing track segments. These missing track segments are attributed to out-of-focus emitters. This is most clearly evidenced by noting that one emitter's average intensity distribution is broader in the first three columns of Fig. 4. More quantitatively, this observation is because this emitter, as seen in the second row of Fig. 4, has a $|z|$ position at a distance 200 nm from the in-focus plane. Once the mean displacement is close to or below the lateral diffraction limit (the fourth and fifth columns of Fig. 4), even Set B can no longer be tuned to resolve two emitters in any frame. In contrast, leveraging spatiotemporal information, BNP-Track successfully tracks out-of-focus emitters. Additional comparisons (using quantitative performance metrics) are again provided in table B.4.

Similar to the approach presented in Fig. 4, where emitters are gradually brought closer together to achieve a higher local emitter number density, we can employ a different strategy by fixing the FOV and introducing additional emitters. This test is illustrated in Fig. A.6. As we increment the number of emitters from one to ten within a FOV of approximately $6\,\mu\mathrm{m}^2$, BNP-Track encounters challenges in connecting emitter positions across frames. This results in tracking errors surpassing the nominal diffraction limit and overestimating the diffusion coefficient, akin to the issues observed with TrackMate in Figs. 3e and 3f for three emitters. In essence, BNP-Track can resolve approximately three times as many emitters as TrackMate.

While establishing a precise boundary for the emitter number density regime in which BNP-Track maintains robustness proves challenging, given its substantial dependence on factors such as the system's diffraction limit and the axial positions of emitters, we propose the following heuristic unitless metric for "crowdedness"

$$\text{crowdedness} = \frac{\text{expected number of emitters} \times \text{area occupied by one emitter}}{\text{area of the FOV}}. \tag{C.1}$$

Here, the area occupied by one emitter is approximated by a circle with a radius equal to the nominal diffraction limit. As presented in Fig. A.6, with this metric, BNP-Track remains robust until the crowdedness approaches about 0.4.

C.3 Robustness tests for motion models

Thus far, we have validated the effectiveness of BNP-Track using experimental datasets, where the assumption of the real motion model aligning with the currently employed 3D normal diffusion model in BNP-Track cannot be assured. Nevertheless, we can strengthen this argument by incorporating synthetic data explicitly generated with distinct motion models motivated by real experiments where ground truth is known.

The first example has already been presented in Fig. A.3, wherein the data assumes 2D normal diffusion with a diffusion coefficient of $1\,\mu\text{m}^2\text{s}^{-1}$ (see Supplementary Data 9), similar to when the emitters are laterally diffusing on a membrane [6, 7]. Despite BNP-Track successfully tracking under the assumption of 3D normal diffusion, we briefly take note of BNP-Track's diffusion coefficient estimate under this scenario. In the context of isotropic diffusion, the discrepancy in motion dimensions implies that the actual mean squared displacement is two-thirds of its expected value. Consequently, the effective ground truth diffusion coefficient should be two-thirds of the actual ground truth diffusion coefficient, that is, $0.67\,\mu\text{m}^2\text{s}^{-1}$. This value falls right in the middle of the diffusion coefficient's 95% CIs in Figs. A.3h and A.3l, $0.54\,\mu\text{m}^2\text{s}^{-1}$ to $0.81\,\mu\text{m}^2\text{s}^{-1}$ and $0.54\,\mu\text{m}^2\text{s}^{-1}$ to $0.79\,\mu\text{m}^2\text{s}^{-1}$, respectively.

In addition to variations in motion dimensions, various motion models may be present when the diffusion coefficient varies across space [8], time [9], or different emitters [10]. For each of these cases, we generated synthetic data (Supplementary Data 27–29), and the results are depicted in Figs. A.7 to A.9, respectively. Once again, BNP-Track successfully tracks with a localization resolution of approximately 30 nm. Since all these datasets involve more than one diffusion coefficient and BNP-Track current assumes one constant diffusion coefficient, the best result it can produce would be an average of the ground truth values. This average is calculated as the weighted average of all diffusion coefficients, where the weights are proportional to the total time the emitters have spent in each state. Further extensions of BNP-Track, which incorporate changes in diffusion coefficient, despite being possible, are out of the scope of this paper.

In Figs. A.7e and A.8e, the average ground truth diffusion coefficients fall within their respective 95% CIs ($0.068\,\mu\text{m}^2\text{s}^{-1}$ versus $0.055\,\mu\text{m}^2\text{s}^{-1}$ to $0.084\,\mu\text{m}^2\text{s}^{-1}$ and $0.05\,\mu\text{m}^2\text{s}^{-1}$ versus $0.044\,\mu\text{m}^2\text{s}^{-1}$ to $0.071\,\mu\text{m}^2\text{s}^{-1}$). However, when emitters have a temporally dependent diffusion coefficient (Fig. A.9e), the average ground truth of $0.077\,\mu\text{m}^2\text{s}^{-1}$ falls outside the 95% CI is $0.083\,\mu\text{m}^2\text{s}^{-1}$ to $0.13\,\mu\text{m}^2\text{s}^{-1}$. Importantly, it should be noted that BNP-Track's estimation aligns with that derived from TrackMate combined with MSD, which is $0.10\,\mu\text{m}^2\text{s}^{-1}$.

D Supplementary Notes

D.1 Probabilistic modeling

Because most of the notions in this study are stochastic, we use probabilistic relations and statistical notations. The methodology we adopt facilitates the description of the variables in BNP-Track and the relations among them. Since the statistical conventions we follow are standard, we provide only a brief description here. For a complete presentation, we refer to [11–14]. Also, a comprehensive introduction to the computational schemes we implement can be found in [13, 15] and complete descriptions in [14, 16, 17].

D.1.1 Statistical notation

A statistical notation like $\theta \sim \text{P}$ indicates that θ is a random variable, and this random variable follows the probability distribution P. In our computations, the values of θ follow the probability density $p(\theta)$ associated with P. For example, $\theta \sim \text{Normal}\left(\mu, \sigma^2\right)$, which means that θ is a normal random variable with mean μ and variance σ^2, indicates that θ is distributed according to the density $p(\theta) = \frac{1}{\sqrt{2\pi\sigma^2}} \exp\left(-\frac{(\mu-\theta)^2}{2\sigma^2}\right)$.

Further, a notation like $w|\theta \sim \text{P}_\theta$ indicates that w is a random variable whose distribution depends on another random variable θ. For example, $w|\theta \sim \text{Normal}\left(\theta, s^2\right)$, indicates that w is distributed according to the density $p(w|\theta) = \frac{1}{\sqrt{2\pi s^2}} \exp\left(-\frac{(\theta-w)^2}{2s^2}\right)$.

Our random variables do not necessarily have to be univariate. As such, w or θ can encompass multiple individual random variables that describe various aspects of our image processing model. The densities $p(w)$ and $p(w|\theta)$ may also depend on other non-random parameters, which we do not explicitly include on the left-hand side of statistical equations.

We summarize the distributions we use in this study and their densities in table B.5. Because some distributions do not have a standardized parameterization, this table also clarifies our particular choices.

D.1.2 Statistical inference

The probability density of two random variables, w and θ, denoted by $p(w, \theta)$, can be expressed as the product of their density functions, i.e., $p(w, \theta) = p(w|\theta)p(\theta)$. This relationship is symmetric, as shown by the equality $p(w, \theta) = p(\theta, w) = p(w|\theta)p(\theta) = p(\theta|w)p(w)$. Therefore, the density function $p(\theta|w)$ is proportional to the product $p(w|\theta)p(\theta)$. In other words, the specification of the distributions of $\theta \sim \mathrm{P}$ and $w|\theta \sim \mathrm{P}_\theta$ is sufficient to derive the density function $p(\theta|w)$, and no other information is required.

Throughout this study, we use w to gather the image measurements and θ to gather the variables whose values we seek to estimate. In this setting, the distributions of θ and $\theta|w$, or their densities $p(\theta)$ and $p(\theta|w)$, are designated as prior and posterior, respectively. These two are linked via $w|\theta$, or its density $p(w|\theta)$, designated as likelihood. As we demonstrate in Results, our approach focuses on the posterior $p(\theta|w)$. In Methods, we describe this posterior by formulating each factor $p(w|\theta)$ and $p(\theta)$ separately. On appendices D.7 and D.8, we describe in detail how each of these factors is mathematically represented.

D.1.3 Statistical simulation

While the posterior distribution $p(\theta|w)$ is proportional to the product of $p(w|\theta)$ and $p(\theta)$, there is no mathematical formula that enables a direct evaluation of this distribution. However, this paper presents a method for indirectly computing the posterior through *sampling simulations*. Specifically, we generate a sequence of values $\theta^{(1)}, \theta^{(2)}, \theta^{(3)}, \ldots$ that have the same statistical properties as $p(\theta|w)$. We use this sequence to produce results such as histograms, scatter plots, mean values, and credible intervals without relying on an analytical formula for $p(\theta|w)$. In appendix D.10, we provide a detailed description of how to carry out posterior sampling in BNP-Track efficiently.

To facilitate the interpretation of our results, we present a simplified example in Fig. D.1 where we simulate from a *toy posterior* $p(\theta|w)$. We consider a bivariate random variable $\theta = (\theta_1, \theta_2)$ in this example. Therefore, we generate two simultaneous sequences of samples, $\theta_1^{(1)}, \theta_1^{(2)}, \theta_1^{(3)}, \ldots$ and $\theta_2^{(1)}, \theta_2^{(2)}, \theta_2^{(3)}, \ldots$, with the specific values shown in the left panels. Using these sequences, we construct histograms in the middle panels to visualize the distribution of each variable and a scatter plot in the right panel to illustrate the correlation between θ_1 and θ_2. It is important to note that if our posterior consisted of more than two random variables, such as the posterior described in Methods, we would require more than two simultaneous sequences of samples to generate more than two histograms and multiple scatter plots to visualize all correlations developed fully.

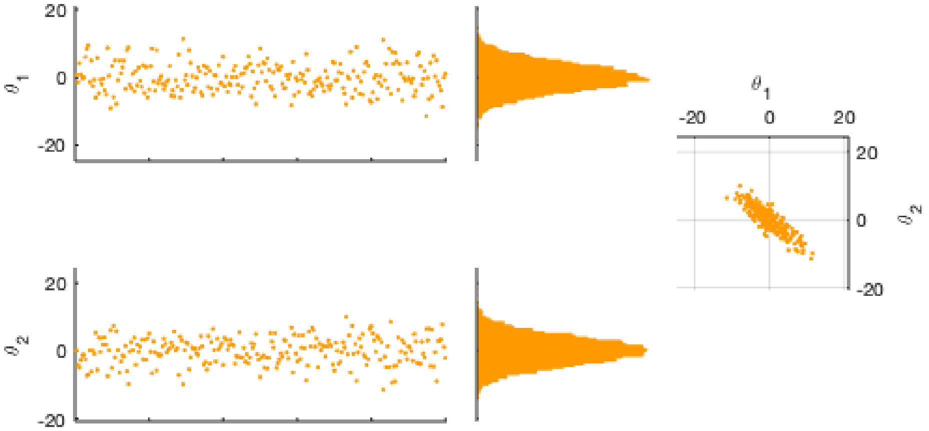

Figure D.1: Sampling from a toy posterior $p(\theta|w)$ with a bivariate variable $\theta = (\theta_1, \theta_2)$ fully characterizes the statistics of the involved variables as well as the correlations among them.

D.2 Frame of reference

In this study, we exclusively consider an object space frame of reference. Specifically, in the object space, we consider Cartesian coordinates x, y, z as illustrated on the left side of Fig. D.2. Consistent with common practice [18–21], our z axis is parallel to the microscope's optical axis and points away from the image plane. Specifically, on an inverted microscope, the z axis points upwards. Moreover, we assume that the microscope and the camera are aligned such that the image plane is perpendicular to the optical axis [18–21]. Lastly, we orient the x, y axes in such a way that they are parallel to the pixel edges of the camera. Overall, this coordinate system follows the right-hand rule.

Under these conventions, the image plane, physically lying on the camera, can be projected to the object plane, physically located on the object space. The object plane is the plane in the sample space yielding focused images, as shown on the right of Fig. D.2. Assuming that the origins in the object space and image plane are conjugated, image plane coordinates x', y' are related to object space coordinates x, y, z by

$$x = \frac{x'}{\mathcal{M}}, \qquad y = \frac{y'}{\mathcal{M}}, \qquad z = 0$$

where $\mathcal{M}$ is the combined magnification achieved through the microscope's objective and tube lenses. With this convention, a square pixel with the physical size of 16 μm, on a microscope imaging at 120x, is projected on a pixel of 133.33 nm.

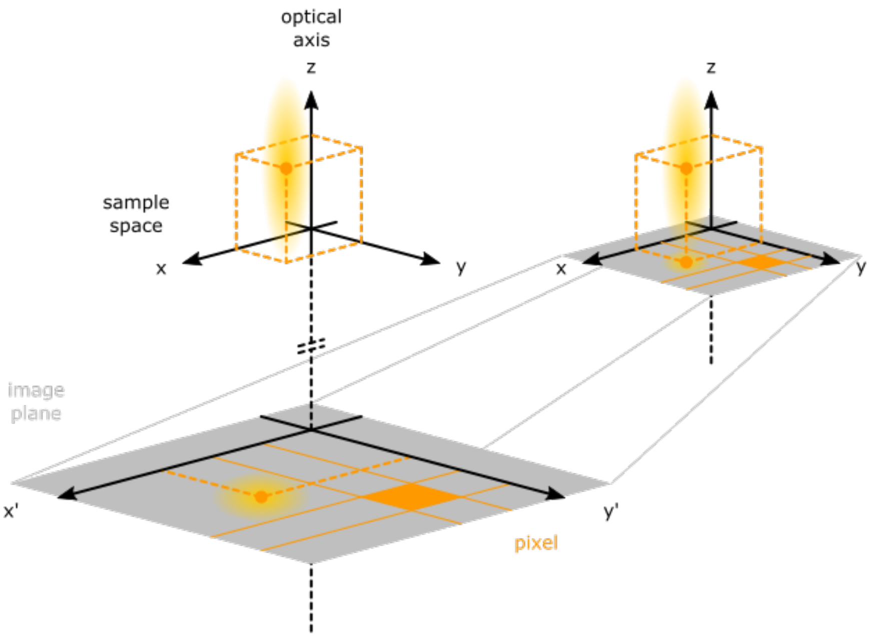

Figure D.2: Frames of reference. Upper left: object space in front of the microscope's lenses along the optical path. Lower left: image plane, located behind the microscope's lenses along the optical path. Right: object space with the image plane projected on the object plane. In this study, we use exclusively the latter configuration where the image plane is identified with the object plane, and position coordinates are expressed in sample space units.

D.3 Time schedule

In this study, we refer to time in relation to the acquisition schedule of the images, which is determined by the camera used. The acquisition of each image involves two phases: an integration period, during which the camera pixels are sensitive to incident photons, and a dead period, during which the camera pixels remain unresponsive to incident photons [22–24]. To improve the accuracy of dynamical approximations, as described in detail in appendix D.5, we model the dead time associated with image acquisition as occurring in two equal periods: one immediately before and one immediately after the corresponding integration period. Our convention is illustrated in Fig. D.3. Analytically, our temporal frame of reference is as follows.

We label the images sequentially with $n = 1, \dots, N$, where $n = 1$ marks the earliest acquired image in the experiment session and $n = N$ is the latest. As shown in Fig. D.3, we denote with t_n^{min} and t_n^{max} the start and end times of the integration period of the nth image. We sometimes report our estimates, which we denote with t_n and label with $n = 0, 1, \dots, N$, that separate successive image acquisitions. Specifically, we distribute the total dead time τ_n^{dead}, associated with the nth image, equally between the time intervals immediately preceding t_n^{min} and following t_n^{max}. With this choice, the acquisition of the nth image is separated by the preceding and following one at the respective times $t_{n-1} = t_n^{\text{min}} - \tau_n^{\text{dead}}/2$ and $t_n = t_n^{\text{max}} + \tau_n^{\text{dead}}/2$.

Provided the frame rate ν^{frame} and dead time τ^{dead} remain constant throughout the imaging course, as it is common in most experiments, our convention becomes

$$t_n = \frac{n}{\nu^{\text{frame}}}, \qquad t_n^{\text{min}} = t_{n-1} + \frac{\tau^{\text{dead}}}{2}, \qquad t_n^{\text{max}} = t_n - \frac{\tau^{\text{dead}}}{2}.$$

For example, with this convention, five images acquired at a frame rate of 10 Hz and a dead time of 10 ms correspond to

$$t_1 = 100 \text{ ms}, \qquad t_2 = 200 \text{ ms}, \qquad t_3 = 300 \text{ ms}, \qquad t_4 = 400 \text{ ms}, \qquad t_5 = 500 \text{ ms},$$

and the individual integration periods last between

$$\begin{aligned} t_1^{\text{min}} &= 5 \text{ ms}, & t_2^{\text{min}} &= 105 \text{ ms}, & t_3^{\text{min}} &= 205 \text{ ms}, & t_4^{\text{min}} &= 305 \text{ ms}, & t_5^{\text{min}} &= 405 \text{ ms}, \\ t_1^{\text{max}} &= 95 \text{ ms}, & t_2^{\text{max}} &= 195 \text{ ms}, & t_3^{\text{max}} &= 295 \text{ ms}, & t_4^{\text{max}} &= 395 \text{ ms}, & t_5^{\text{max}} &= 495 \text{ ms}. \end{aligned}$$

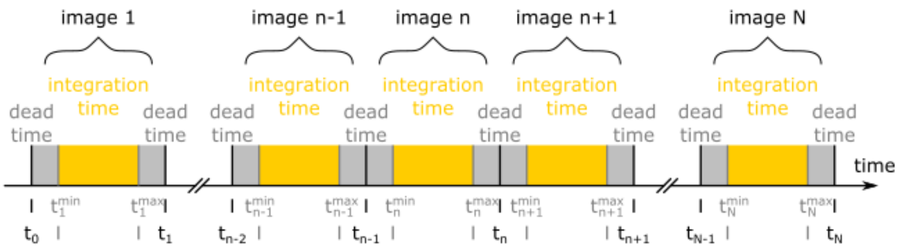

Figure D.3: Temporal frame of reference. In our convention, the acquisition of each image contains three separate phases: a dead time phase right before the integration time, an integration time phase, and a dead time phase right after the integration time. Specifically, for the nth image the three phases are distinguished by the times $t_{n-1}, t_n^{\text{min}}, t_n^{\text{max}}, t_n$.

D.4 Optics representation

Due to the incoherent imaging conditions encountered in fluorescence microscopy [18–21, 23], we describe the optical response of the imaging apparatus with an additive photon flux of the form

$$U(x,y,t) = U_{\text{back}}(x,y,t) + \sum_{m=1}^{M} b^m U_{\text{fluor}}^m(x,y,t)$$

that sums up background photon contributions $U_{\text{back}}(x,y,t)$ and emitter photon contributions $U_{\text{fluor}}^m(x,y,t)$. Our photon flux $U(x,y,t)$ models the irradiance [24–26] on the image plane, which according to appendix D.2 we project to the object plane in the sample space, as it changes over the time course of the imaging experiment t.

In turn, we model $U_{\text{back}}(x,y,t) = C(t)$ as a uniform-in-space flux, which we denote with $C(t)$, while we model fluorophore contributions by summing flux contributions of each emitter $U_{\text{fluor}}^m(x,y,t)$ which we label with $m = 1,\ldots,M$. We model the latter by the product

$$U_{\text{fluor}}^m(x,y,t) = h(t)\, G_g\left(x,y;X^m(t),Y^m(t),Z^m(t)\right).$$

This product consists of a point spread function $G_g(x,y;X,Y,Z)$, which models how the incident photons stemming from a light emitter at position X,Y,Z are spread over the image plane [18, 19, 21, 27–29]; and, a photon emission rate $h(t)$, which describes how often a single light emitter emits photons incident on the image plane.

From the two factors forming each $U_{\text{fluor}}^m(x,y,t)$, the point spread function $G_g(x,y;X,Y,Z)$ is measured in units of 1/area and the photon emission rate $h(t)$ is measured in units of photons/time. Together, these result in a flux with units of photons/area/time.

In superresolution microscopy, the point spread function is primarily affected by diffraction and microscope-specific aberrations [18, 19, 21, 27–30] and the photon emission rate is set by the labeling fluorophores and the illumination modality applied [23, 24]. For these reasons, we obtain $G_g(x,y;X,Y,Z)$ by fitting calibration measurements, as described in appendix D.11, and $h(t)$ by fitting during processing, as described in appendix D.10.

D.4.1 Point spread function

For all light emitters, we consider Gaussian point spread functions [18, 27, 31, 32]. These have the form

$$G_g(x,y;X,Y,Z) = \frac{1}{2\pi\sigma_g^2(Z)} \exp\left(-\frac{1}{2}\frac{(x-X)^2+(y-Y)^2}{\sigma_g^2(Z)}\right).$$

By convention, we normalize each point spread function to unit total volume, i.e.

$$\iint_{-\infty,-\infty}^{+\infty,+\infty} dxdy\, G_g(x,y;X,Y,Z) = 1,$$

so we can readily compare photon emissions from in-focus and out-of-focus emitters.

The extent of the point spread function, which is proportional to $\sigma_g(Z)$, depends upon the position Z of the emitter along the optical axis [21, 28–30]. Assuming the axial light propagation is similar to a Gaussian

beam [20, 31], we model $\sigma_g(Z)$ by

$$\sigma_g(Z) = \sigma_{\text{ref}} \sqrt{g_1 + g_2 \left(\frac{Z}{Z_{\text{ref}}}\right)^2},$$

where σ_{ref} and Z_{ref} are reference values for our point spread function with units of length and g_1, g_2 are unitless parameters that allow our point spread function to accommodate microscope specific aberrations in the width of the focused point spread function and the depth of focus. Namely, g_1 adjusts for deviations in the width of the point spread function of an in-focus emitter, $Z = 0$, while g_2 adjusts for deviations in the width of the point spread function of an out-of-focus emitter, $Z \neq 0$.

We obtain reference values $\sigma_{\text{ref}}, Z_{\text{ref}}$ considering diffraction-limited imaging under ideal conditions. Specifically, following [18, 21, 24, 26, 27, 32] we use

$$\sigma_{\text{ref}} = 0.21 \frac{\lambda}{n_{\text{NA}}} \qquad\qquad Z_{\text{ref}} = 4\pi n_{\text{RI}} \frac{\sigma_{\text{ref}}^2}{\lambda}$$

where n_{NA} denotes the microscope's numerical aperture, λ denotes the imaged wavelength (in vacuum), and n_{RI} denotes the index of refraction of the objective's immersion fluid. However, the values of g_1, g_2 must be adjusted to fit each particular microscope. This is achieved following the calibration protocol in appendix D.11.

D.4.2 Effective pixel function

Combining the flux $U(x, y, t)$ with the pixel response, which integrates photon contributions over space and time [23, 24, 33], the average number of incident photons on the pth pixel during the nth exposure takes the form

$$\begin{aligned} u_n^p &= \int_{t_n^{\min}}^{t_n^{\max}} dt \iint_{x_{\min}^p, y_{\min}^p}^{x_{\max}^p, y_{\max}^p} dxdy\, U(x, y, t) \\ &= \int_{t_n^{\min}}^{t_n^{\max}} dt \left(C(t) A^p + h(t) \sum_{m=1}^{M} b^m \iint_{x_{\min}^p, y_{\min}^p}^{x_{\max}^p, y_{\max}^p} dxdy\, G_g(x, y; X^m(t), Y^m(t), Z^m(t)) \right) \end{aligned}$$

where $A^p = (x_{\max}^p - x_{\min}^p)(y_{\max}^p - y_{\min}^p)$ is the area monitored by the pth pixel.

To simplify the notation from now on, for each pixel, we consider an effective function defined by

$$Q_g^p(X, Y, Z) = \iint_{x_{\min}^p, y_{\min}^p}^{x_{\max}^p, y_{\max}^p} dxdy\ G_g(x, y; X, Y, Z),$$

which combines the effects of diffraction and finite pixel size. Our effective functions are unitless. With this convention, the average number of photons incident on the pth pixel during the nth exposure becomes

$$u_n^p = \int_{t_n^{\min}}^{t_n^{\max}} dt \left(C(t) A^p + h(t) \sum_{m=1}^{M} b^m Q_g^p(X^m(t), Y^m(t), Z^m(t)) \right).$$

D.5 Dynamics representation

D.6 Temporal discretization

To proceed with image analysis, the average numbers of incident photons $u_{1:N}^{1:P}$ over all pixels and exposures need to be related to dynamical, temporally discretized variables. For this, we approximate the integral of

$$u_n^p = \int_{t_n^{\min}}^{t_n^{\max}} dt \left(C(t)A^p + h(t) \sum_{m=1}^{M} b^m Q_g^p(X^m(t), Y^m(t), Z^m(t)) \right).$$

Ideally, we seek to evaluate this integral analytically. However, due to the unknown forms of both terms in the integrand, namely (i) $C(t)$, which depends on the specific experimental system, and (ii) the stochastic spatial trajectory of the emitter between $t_n^{\min}$ and $t_n^{\max}$, the aforementioned integral must be approximated numerically.

Our approximations rely on the following numerical quadrature formulas

$$\int_{t_n^{\min}}^{t_n^{\max}} dt\, C(t) \approx \tau_n^{\text{exps}} C_n,$$

$$\int_{t_n^{\min}}^{t_n^{\max}} dt\, h(t) Q_g^p(X^m(t), Y^m(t), Z^m(t)) \approx \tau_n^{\text{exps}} h_n Q_g^p(X_n^m, Y_n^m, Z_n^m),$$

where $\tau_n^{\text{exps}} = t_n^{\max} - t_n^{\min}$ is the integration time of the nth exposure and the discretized variables $C_{1:N}$, $h_{1:N}$, $X_{1:N}^{1:M}$, $Y_{1:N}^{1:M}$, and $Z_{1:N}^{1:M}$ are related to their continuous counterparts $C(t)$, $h(t)$, $X^{1:M}(t)$, $Y^{1:M}(t)$, and $Z^{1:M}(t)$ according to

$$C_n = C(T_n), \qquad h_n = h(T_n), \qquad X_n^m = X^m(T_n), \qquad Y_n^m = Y^m(T_n), \qquad Z_n^m = Z^m(T_n).$$

Finally, the discretized time levels $T_{1:N}$ are given by

$$T_n = \frac{t_n^{\min} + t_n^{\max}}{2}.$$

In essence, we use the mid-point rules [34–37] to approximate the quadrature of $C(t)$, $h(t)$, $X^{1:M}(t)$, $Y^{1:M}(t)$, and $Z^{1:M}(t)$. As explained in appendix D.3, since the times $t_{0:N}$ are centered, both rules result in errors that decrease super-linearly concerning the frame rate when the frame rate remains constant over the imaging course.

Ignoring the errors introduced by the quadrature formulas, from now on, we consider average numbers of incident photons $u_{1:N}^{1:P}$ that we evaluate according to

$$u_n^p = \tau_n^{\text{exps}} \left(C_n A^p + h_n \sum_{m=1}^{M} b^m Q_g^p(X_n^m, Y_n^m, Z_n^m) \right).$$

Since in typical experiments, background arises from uncharacterized sources and the photon emission rate is affected by uncontrolled factors, in this study, we do not model the variables $C_{1:N}$ and $h_{1:N}$ any further. However, to link each emitter's discretized positions $X_{1:N}^m$, $Y_{1:N}^m$, and $Z_{1:N}^m$ across time, we invoke physically realistic dynamical models of motion that we describe below.

D.6.1 Simplification for time-independent emitter brightness and background photon flux

As mentioned in the main text, for the sake of easier comparison of BNP-Track to other single particle tracking tools, we remove the time-dependence in emitter brightness and background flux to decrease the number of inferred quantities BNP-Track needs to estimate (where background flux, for instance, would need to be provided to competing tools). This can easily be done by modifying the equation above to

$$u_n^p = \tau_n^{\text{exps}} \left(CA^p + h \sum_{m=1}^{M} b^m Q_g^p(X_n^m, Y_n^m, Z_n^m) \right).$$

Here, h and C are constant values for emitter brightness and background photon flux.

D.6.2 Emitter motion

To represent emitter motion, we consider dynamics consistent with free Brownian motion [35, 38–43]. Accordingly, successive positions X_n^m, Y_n^m, Z_n^m and $X_{n+1}^m, Y_{n+1}^m, Z_{n+1}^m$ or $X_{n-1}^m, Y_{n-1}^m, Z_{n-1}^m$ and X_n^m, Y_n^m, Z_n^m along an emitter's trajectory are linked via independent Normal increments of zero mean and variance equal to $2D(T_{n+1}-T_n)$ or $2D(T_n-T_{n-1})$, respectively. In our model, D indicated the emitters' diffusion coefficient.

For successive positions before a given time level, this leads to

$$\begin{aligned} X_n^m \big| X_{n+1}^m, D &\sim \text{Normal}\left(X_{n+1}^m, 2D(T_{n+1} - T_n)\right), \\ Y_n^m \big| Y_{n+1}^m, D &\sim \text{Normal}\left(Y_{n+1}^m, 2D(T_{n+1} - T_n)\right), \qquad n = 1, \dots, K^m - 1 \\ Z_n^m \big| Z_{n+1}^m, D &\sim \text{Normal}\left(Z_{n+1}^m, 2D(T_{n+1} - T_n)\right), \end{aligned}$$

while, for successive positions after a given time level, leads to

$$\begin{aligned} X_n^m \big| X_{n-1}^m, D &\sim \text{Normal}\left(X_{n-1}^m, 2D(T_n - T_{n-1})\right), \\ Y_n^m \big| Y_{n-1}^m, D &\sim \text{Normal}\left(Y_{n-1}^m, 2D(T_n - T_{n-1})\right), \qquad n = K^m + 1, \dots, N \\ Z_n^m \big| Z_{n-1}^m, D &\sim \text{Normal}\left(Z_{n-1}^m, 2D(T_n - T_{n-1})\right). \end{aligned}$$

Similar to all models representing Brownian motion, our formulation leaves one position per trajectory unspecified. Namely, in our notation, the unspecified position of the mth emitter's trajectory corresponds to time level $n = K^m$ and needs to be modeled separately.

To model experiments under conditions that resemble a stationary state, in which the statistics of the dynamical variables remain stable over the imaging course, we obtain the position left unspecified by

$$\begin{aligned} X_n^m &\sim \text{Uniform}_{[X_{\min}, X_{\max}]}, \\ Y_n^m &\sim \text{Uniform}_{[Y_{\min}, Y_{\max}]}, \qquad n = K^m \\ Z_n^m &\sim \text{Uniform}_{[Z_{\min}, Z_{\max}]} \end{aligned}$$

and the corresponding time level by

$$K^m \sim \text{Uniform}_{1:N}.$$

Overall, our representation of an emitter's motion is equivalent to the following Brownian probability density

$$\begin{aligned}
p\left(X_{1:N}^m, Y_{1:N}^m, Z_{1:N}^m, K^m\right) = {} & \text{Uniform}_{[X_{\min}, X_{\max}]}\left(X_{K^m}^m\right) \prod_{n=1}^{N-1} \text{Normal}\left(X_{n+1}^m - X_n^m; 0, 2D(T_{n+1} - T_n)\right) \cdots \\
& \times \text{Uniform}_{[Y_{\min}, Y_{\max}]}\left(Y_{K^m}^m\right) \prod_{n=1}^{N-1} \text{Normal}\left(Y_{n+1}^m - Y_n^m; 0, 2D(T_{n+1} - T_n)\right) \cdots \\
& \times \text{Uniform}_{[Z_{\min}, Z_{\max}]}\left(Z_{K^m}^m\right) \prod_{n=1}^{N-1} \text{Normal}\left(Z_{n+1}^m - Z_n^m; 0, 2D(T_{n+1} - T_n)\right) \cdots \\
& \times \text{Uniform}_{1:N}(K^m) p\left(X_1^m, Y_1^m, Z_1^m, K^m\right) \\
& \propto \prod_{n=1}^{N-1} \text{Normal}\left(X_{n+1}^m - X_n^m; 0, 2D(T_{n+1} - T_n)\right) \cdots \\
& \times \prod_{n=1}^{N-1} \text{Normal}\left(Y_{n+1}^m - Y_n^m; 0, 2D(T_{n+1} - T_n)\right) \cdots \\
& \times \prod_{n=1}^{N-1} \text{Normal}\left(Z_{n+1}^m - Z_n^m; 0, 2D(T_{n+1} - T_n)\right) \\
& \times p\left(X_1^m, Y_1^m, Z_1^m\right).
\end{aligned}$$

The proportionality above shows that our representation of the emitters' motion is consistent with studies characterizing single particle motion and diffusion coefficients using mean square displacements [35, 43, 44]. In addition, $p\left(X_1^m, Y_1^m, Z_1^m\right)$ is the prior placed on an emitter's initial position.

D.6.3 Extended BNP-Track for other motion models

Up to this point, we have discussed emitter motion through free Brownian motion in three dimensions. Nevertheless, BNP-Track can incorporate various motion models, provided their justification. In this context, we offer a concise demonstration of BNP-Track's adaptability to encompass motion models wherein the diffusion coefficient exhibits spatial, temporal, or emitter dependencies.

In all of these scenarios, we can introduce an additional variable, denoted as S_n^m, representing the diffusive state of emitter m at time point n. Subsequently, the probability density governing the motion of this emitter becomes:

$$\begin{aligned}
p\left(X_{1:N}^m, Y_{1:N}^m, Z_{1:N}^m, S_{1:N}^m, K^m\right) \propto {} & \prod_{n=1}^{N-1} \text{Normal}\left(X_{n+1}^m - X_n^m; 0, 2D_{S_n^m}(T_{n+1} - T_n)\right) \cdots \\
& \times \prod_{n=1}^{N-1} \text{Normal}\left(Y_{n+1}^m - Y_n^m; 0, 2D_{S_n^m}(T_{n+1} - T_n)\right) \cdots \\
& \times \prod_{n=1}^{N-1} \text{Normal}\left(Z_{n+1}^m - Z_n^m; 0, 2D_{S_n^m}(T_{n+1} - T_n)\right) \\
& \times p\left(X_1^m, Y_1^m, Z_1^m\right) p\left(S_{1:N}^m\right).
\end{aligned}$$

Here, $D_{S_n^m}$ represents the diffusion coefficient of state S_n^m, and $p\left(S_{1:N}^m\right)$ denotes the probability density of the diffusive state trajectory. If the diffusion coefficient is emitter-dependent, we set $S_n^m = m$ and remove $p\left(S_{1:N}^m\right)$ since it is now deterministic.

When an emitter's diffusive state exhibits spatial dependency exclusively, $p\left(S_{1:N}^m\right)$ should be revised as follows:

$$p\left(S_{1:N}^m | X_{1:N}^m, Y_{1:N}^m, Z_{1:N}^m\right) = \prod_{n=1}^{N} p\left(S_n^m | X_n^m, Y_n^m, Z_n^m\right)$$

where $p\left(S_n^m | X_n^m, Y_n^m, Z_n^m\right)$ depends on the specific system. For instance, if there is a boundary denoted by $x = x'$ separating two regions with distinct diffusion coefficients, we can formulate

$$p\left(S_n^m = 1 | X_n^m, Y_n^m, Z_n^m\right) \begin{cases} 1, & X_n^m \leq x', \\ 0, & X_n^m > x'; \end{cases}$$

and

$$p\left(S_n^m = 2 | X_n^m, Y_n^m, Z_n^m\right) \begin{cases} 0, & X_n^m \leq x', \\ 1, & X_n^m > x'. \end{cases}$$

Similarly, in scenarios where the diffusion coefficient is time-dependent and under the assumption of the Markov (memoryless) property, we modify $p\left(S_{1:N}^m\right)$ to

$$p\left(S_{1:N}^m | \Pi\right) = p\left(S_1^m\right) \prod_{n=1}^{N-1} p\left(S_{n+1}^m | S_n^m, \Pi\right)$$

where Π is the transition probability matrix for diffusive states.

It is crucial to emphasize that the extensions outlined in this section will augment the computational cost of BNP-Track. This is because the trajectories of the diffusive states, $S_{1:N}^{1:M}$, are frequently unknown, thereby introducing additional variables that require inference.

D.6.4 Extended BNP-Track to estimate multiple positions of one emitter within one frame

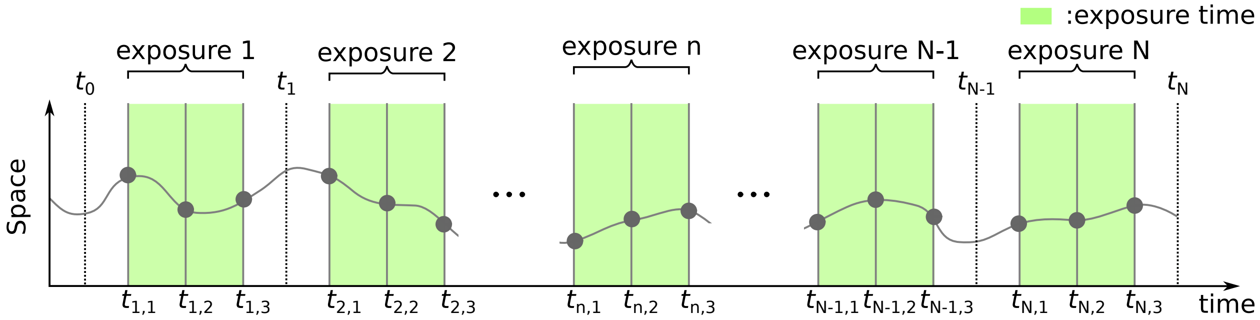

Figure D.4: How BNP-Track can be extended to consider intra-exposure emitter motion. This figure shows an example of estimating three positions for each emitter within each exposure.

As a general tracking framework, BNP-Track can be extended to estimate multiple positions of one emitter within one frame, see Fig. D.4. This is important when emitters move appreciably compared to a pixel dimension within one exposure, resulting in motion blur, also termed aliasing, which distorts the PSF [18]. To address this issue, we can better approximate the first equation in appendix D.5 using the trapezoidal rule [45, 46]. To be more specific, we can replace our simple quadrature rule

$$\int_{t_n^{\min}}^{t_n^{\max}} dt\, h(t) Q_g^p(X^m(t), Y^m(t), Z^m(t)) \approx \tau_n^{\text{exps}}\, h_n Q_g^p(X_n^m, Y_n^m, Z_n^m)$$

with a composite one as follows

$$\int_{t_n^{\min}}^{t_n^{\max}} dt\, h(t) Q_g^p(X^m(t), Y^m(t), Z^m(t))$$
$$\approx \frac{\tau_n^{\text{exps}}}{K-1} \sum_{k=1}^{K-1} \left[h_{n,k} Q_g^p(X_{n,k}^m, Y_{n,k}^m, Z_{n,k}^m) + h_{n,k+1} Q_g^p(X_{n,k+1}^m, Y_{n,k+1}^m, Z_{n,k+1}^m) \right].$$

Here, K is the number of discretized time points in an exposure, and we define $h_{n,k} = h(t_{n,k})$, and $X_{n,k}^m = X^m(t_{n,k})$. As K approaches infinity, the evaluation of the time integral becomes exact with the quadratic rate of convergence [47]. In practice, as illustrated in Fig. A.3, when confronted with a diffusion coefficient of $1\,\mu\text{m}^2\text{s}^{-1}$ and a camera exposure time of approximately 30 ms, the utilization of five subintervals for the composite trapezoidal rule proves to be adequate.

D.7 Model likelihood

In the preceding sections, we outlined our spatial and temporal frames of reference (appendices D.2 and D.3), within which we define the various quantities of interest, and our mathematical formulation (appendices D.4 and D.5), which establishes the relationships between those quantities. However, in an imaging experiment, most of the quantities of interest have unknown values. For example, in a typical superresolution experiment, the unknowns include

$$\theta = \left\{C_{1:N}, h_{1:N}, D, b^{1:M}, X^{1:M}_{1:N}, Y^{1:M}_{1:N}, Z^{1:M}_{1:N}, K^{1:M}\right\}.$$

As we explain in appendix D.1, we aim to infer these values through a posterior distribution that uses the images acquired.

In quantitative microscopy, image measurements are provided in the form of image values $w^{1:P}_{1:N}$ obtained from the camera's array of pixel photosensors, which we label with $p = 1, \dots, P$, and a sequence of exposures, which we label with $n = 1, \dots, N$. Following the conventions introduced in appendices D.2, D.3 and D.4.2, an image value w^p_n probes the average photons u^p_n incident on the region of space monitored by the pth pixel during the nth exposure. These correspond to

$$x^p_{\min} < x < x^p_{\max}, \qquad y^p_{\min} < y < y^p_{\max}, \qquad t^{\min}_n < t < t^{\max}_n.$$

Because of shot-noise and noisy camera read-out, the relation between w^p_n and u^p_n is stochastic and varies among different types of cameras. For scientific-grade cameras of the EMCCD type, which are commonly used in superresolution imaging [30, 48], the relationship is given by

$$w^p_n | u^p_n \sim \text{Normal}\left(\mu + \xi u^p_n, \upsilon + f\xi^2 u^p_n\right)$$

where μ, υ, ξ, and f are the EMCCD's read-out offset, variance, overall gain, and excess noise factor [48], respectively. These values are reported in units of ADU, ADU^2, ADU/photon and 1/photon, respectively. Because μ, υ, ξ, and f are camera characteristics and not specimen characteristics, we obtain these values separately following the calibration protocol of appendix D.11.

Since μ, υ, ξ, and f are specified via calibration before image processing, we use adjusted values

$$\bar{w}^p_n = \frac{w^p_n - \mu}{\xi}, \qquad \bar{\upsilon} = \frac{\upsilon}{\xi^2}$$

and cast our model's likelihood in its equivalent form

$$\bar{w}^p_n | u^p_n \sim \text{Normal}\left(u^p_n, \bar{\upsilon} + f u^p_n\right)$$

which allows for faster evaluation. With this convention, according to the definitions of table B.5, our likelihood's probability density takes the form

$$p(\bar{w}^p_n | u^p_n) = \frac{1}{\sqrt{2\pi(\bar{\upsilon} + f u^p_n)}} \exp\left(-\frac{1}{2}\frac{(\bar{w}^p_n - u^p_n)^2}{\bar{\upsilon} + f u^p_n}\right).$$

Finally, we may invoke a Laplace approximation [49, 50], according to

$$p(\bar{w}^p_n | u^p_n) \approx \frac{1}{\sqrt{2\pi(\bar{\upsilon} + f \bar{w}^p_n)}} \exp\left(-\frac{1}{2}\frac{(\bar{w}^p_n - u^p_n)^2}{\bar{\upsilon} + f u^p_n}\right) \propto \exp\left(-\frac{1}{2}\frac{(\bar{w}^p_n - u^p_n)^2}{\bar{\upsilon} + f u^p_n}\right)$$

to speed up the evaluation of our likelihood even further. Under the typical values encountered in a superresolution setting, such an approximation has a negligible effect on our results.

D.8 Model priors

D.8.1 Nonparametric prior distributions

Our method is nonparametric and relies on indicator parameters denoted by $b^{1:M}$, which play a crucial role in our analysis. As we describe in Methods, these parameters are model variables whose values are inferred concurrently with the other unknowns during processing.

To infer the values of $b^{1:M}$, we use independent Bernoulli priors with emitter-specific hyperparameters $q^{1:M}$, given by:

$$b^m | q^m \sim \text{Bernoulli}\left(q^m\right),$$

To avoid overfitting as the number of model emitters grows, we employ Beta hyper-priors of the form:

$$q^m \sim \text{Beta}\left(\frac{\gamma}{M}, 1 - \frac{\gamma}{M}\right)$$

on the Bernoulli weights $q^{1:M}$. With these priors and hyper-priors, our model remains well-defined at the limit $M \to \infty$, where it converges to a Beta-Bernoulli process [51–55]. The choice of M has no interpretational side effects and only affects computation speed. Provided M is sufficiently large, the precise value of M has an insignificant influence on the results.

Because the prior and hyper-prior on each b^m is independent of the others, we can combine them into a single distribution which takes the form

$$b^m \sim \text{Bernoulli}\left(\frac{\gamma}{M}\right)$$

and, because the total number of imaged emitters $B = \sum_{m=1}^{M} b^m$ is a derived variable, we can derive its prior statistics. In particular, as a sum of independent Bernoulli variables, the induced prior is

$$B \sim \text{Binomial}\left(M, \frac{\gamma}{M}\right)$$

which, at the limit $M \to \infty$, converges to a Poisson(γ) random variable. Accordingly, γ can be interpreted as the prior mean number of light emitters anticipated to contribute photons in the supplied images.

D.8.2 Parametric prior distributions

We have already described our choices for the nonparametric prior on the indicators $b^{1:M}$ as well as appropriate dynamical representations for the emitter positions $X_{1:N}^{1:M}$, $Y_{1:N}^{1:M}$, $Z_{1:N}^{1:M}$, and $K^{1:M}$. To complete our model, for the remaining unknowns $C_{1:N}$, $h_{1:N}$, and D we make the following choices.

On the background photon fluxes $C_{1:N}$ and emitter photon emission rates $h_{1:N}$ we place independent Gamma priors

$$C_n \sim \text{Gamma}\left(A_n^C, \frac{C_n^{\text{ref}}}{A_n^C}\right),$$
$$h_n \sim \text{Gamma}\left(A_n^h, \frac{h_n^{\text{ref}}}{A_n^h}\right),$$

These depend on reference values $C_{1:N}^{\text{ref}}$ and $h_{1:N}^{\text{ref}}$ that set prior scales and provide units, as well as on dimensionless parameters $A_{1:N}^C$ and $A_{1:N}^h$ that can be used to model prior confidence on the assigned reference

values $C_{1:N}^{\mathrm{ref}}$ and $h_{1:N}^{\mathrm{ref}}$ or lack thereof. For example, setting $A_n^C \gg 1$ restricts the background fluxes $C_{1:N}$ to attain values near C_n^{ref} and similarly for $h_{1:N}$.

Finally, on the diffusion coefficient D, we place an Inverse-Gamma prior

$$D \sim \mathrm{InvGamma}\left(\alpha_D, (\alpha_D - 1)D_{\mathrm{ref}}\right)$$

with, similarly, prior scale and units set by D_{ref} and prior confidence set by α_D.

D.9 Summary of model equations

The following equations summarize our entire Bayesian nonparametric imaging data processing method.

$$
\begin{aligned}
C_n &\sim \text{Gamma}\left(A_n^C, C_n^{\text{ref}}/A_n^C\right), && n=1,\dots,N \\
h_n &\sim \text{Gamma}\left(A_n^h, h_n^{\text{ref}}/A_n^h\right), && n=1,\dots,N \\
\\
D &\sim \text{InvGamma}\left(\alpha_D, (\alpha_D-1)D_{\text{ref}}\right), \\
\\
b^m &\sim \text{Bernoulli}\left(\gamma/M\right), && && m=1,\dots,M \\
\\
K^m &\sim \text{Uniform}_{1:N}, && && m=1,\dots,M \\
\\
X_n^m|K^m &\sim \text{Uniform}_{[X_{\min},X_{\max}]}, && n=K^m && m=1,\dots,M \\
X_n^m|X_{n+1}^m,K^m,D &\sim \text{Normal}\left(X_{n+1}^m, 2D(T_{n+1}-T_n)\right), && n=1,\dots,K^m-1 && m=1,\dots,M \\
X_n^m|X_{n-1}^m,K^m,D &\sim \text{Normal}\left(X_{n-1}^m, 2D(T_n-T_{n-1})\right), && n=K^m+1,\dots,N && m=1,\dots,M \\
\\
Y_n^m|K^m &\sim \text{Uniform}_{[Y_{\min},Y_{\max}]}, && n=K^m && m=1,\dots,M \\
Y_n^m|Y_{n+1}^m,K^m,D &\sim \text{Normal}\left(Y_{n+1}^m, 2D(T_{n+1}-T_n)\right), && n=1,\dots,K^m-1 && m=1,\dots,M \\
Y_n^m|Y_{n-1}^m,K^m,D &\sim \text{Normal}\left(Y_{n-1}^m, 2D(T_n-T_{n-1})\right), && n=K^m+1,\dots,N && m=1,\dots,M \\
\\
Z_n^m|K^m &\sim \text{Uniform}_{[Z_{\min},Z_{\max}]}, && n=K^m && m=1,\dots,M \\
Z_n^m|Z_{n+1}^m,K^m,D &\sim \text{Normal}\left(Z_{n+1}^m, 2D(T_{n+1}-T_n)\right), && n=1,\dots,K^m-1 && m=1,\dots,M \\
Z_n^m|Z_{n-1}^m,K^m,D &\sim \text{Normal}\left(Z_{n-1}^m, 2D(T_n-T_{n-1})\right), && n=K^m+1,\dots,N && m=1,\dots,M \\
\\
w_n^p|C_n,h_n,b^{1:M},X_n^{1:M},Y_n^{1:M},Z_n^{1:M} &\sim \text{Normal}\left(\mu+\xi u_n^p, \upsilon+f\xi^2 u_n^p\right), && n=1,\dots,N, && p=1,\dots,P
\end{aligned}
$$

The last equation may be substituted with its equivalent form,

$$\bar{w}_n^p|C_n,h_n,b^{1:M},X_n^{1:M},Y_n^{1:M},Z_n^{1:M} \sim \text{Normal}\left(u_n^p, \bar{\upsilon}+f u_n^p\right), \qquad n=1,\dots,N, \qquad p=1,\dots,P$$

or approximated as described in appendix D.7.

In all equations, the parameters C_n, h_n, $b^{1:M}$, $X_n^{1:M}$, $Y_n^{1:M}$, and $Z_n^{1:M}$ are related to the image measurements by

$$u_n^p = \tau_n^{\text{exps}}\left(C_n A^p + h_n \sum_{m=1}^{M} b^m Q_g^p(X_n^m, Y_n^m, Z_n^m)\right).$$

D.10 Evaluation and interpretation of the model posterior

Our image analysis method, BNP-Track, is entirely contained in the statistical model whose equations are listed in appendix D.9. We have adapted these equations to meet the particular imaging conditions of superresolution microscopy as well as to facilitate the estimation of the variables of typical interest in a biological, biochemical, or biophysical experiment [30] that we detailed in appendices D.2 to D.5.

As we explain in appendix D.1, we encode our model's equations and an experiment's measured images in a posterior probability distribution [11–14]. This distribution assimilates into our analysis the information supplied by the form of the model itself (e.g. information such as parameter ranges or relations among the parameters) and the information provided by the experimental data, which takes the form of image values reported by the camera devices used.

Our posterior distribution, in its complete form, is

$$p\Big(\underbrace{C_{1:N}, h_{1:N}, D, b^{1:M}, X_{1:N}^{1:M}, Y_{1:N}^{1:M}, Z_{1:N}^{1:M}, K^{1:M}}_{\text{unknowns}}\Big|\underbrace{w_{1:N}^{1:P}}_{\text{data}}\Big).$$

For convenience, we define a single variable θ that gathers all unknowns, including $C_{1:N}$, $h_{1:N}$, D, $b^{1:M}$, $X_{1:N}^{1:M}$, $Y_{1:N}^{1:M}$, $Z_{1:N}^{1:M}$, and $K^{1:M}$. We also use W to represent the image measurement, denoted as $w_{1:N}^{1:P}$. This notation can compactly express the posterior distribution as $p(\theta|W)$.

The posterior $p(\theta|W)$ quantifies, in an absolute scale, the consistency between any given assignment of specific values to the unknowns, e.g. an assignment such as $C_1 = 5\times10^4$ photons/s/μm^2, $h_1 = 6\times10^4$ photons/s, $D = 0.11$ μm^2/s and so on, with the measured images. In principle, with $p(\theta|W)$, we can precisely quantify how probable it is for the assigned values to have generated the recorded images; high posterior corresponds to highly probable value assignments, and, vice versa, low posterior corresponds to highly improbable assignments. Nevertheless, due to the nonparametric prior and the non-trivial relations among our unknowns, an exhaustive quantification of all possible value assignments via greedy computations is impossible. This is because, at the limit $M \to \infty$, our infinite number of unknowns $b^{1:M}$, $X_{1:N}^{1:M}$, $Y_{1:N}^{1:M}$, $Z_{1:N}^{1:M}$, $K^{1:M}$, and the non-linear dependencies underlying $u_{1:N}^{1:P}$ lead to intractable formulas.

Instead, we use the posterior $p(\theta|W)$ in a different but statistically equivalent manner. In particular, instead of seeking to quantify exhaustively every possible parameter value assignment, the vast majority of which is highly inconsistent with the measured images, we compute only those value assignments that are most consistent with the images [14]. This way, we quantify value assignment on a relative scale. Practically, as we illustrate in appendix D.1, we generate computationally random assignments so that most probable values occur with higher frequency than the others. As we demonstrate in Results and Fig. D.1, we can derive any statistic of interest by those assignments, which, to avoid confusion, we will call posterior samples.

Next, we describe the steps necessary to generate and handle posterior samples.

D.10.1 Evaluation

We denote the posterior samples we seek to generate with superscripts (i). Specifically, these are $\theta^{(0)}, \theta^{(1)}, \theta^{(2)}, \ldots, \theta^{(i)}, \theta^{(i+1)}, .$ To generate these samples, we develop a Markov chain Monte Carlo scheme [16, 17]. Namely, we use pseudo-random computer simulations to advance from one sample to the next. For this, we apply a custom Gibbs sampling scheme [14–17].

Specifically, we initialize $\theta^{(0)}$ values for all variables by sampling from their respective priors. Subsequently,

to advance from $\theta^{(i)}$ to $\theta^{(i+1)}$, our scheme proceeds in steps during which we update only a designated block of parameters. In detail, we update the parameters in blocks by interlacing, in random order, the following six steps:

1. We update the variables $C_{1:N}$. This step involves sampling from the conditional distribution
$$p\Big(C_{1:N}\Big|h_{1:N}, D, b^{1:M}, X_{1:N}^{1:M}, Y_{1:N}^{1:M}, Z_{1:N}^{1:M}, K^{1:M}, w_{1:N}^{1:P}\Big).$$
2. We update the variables $C_{1:N}$ and $h_{1:N}$. This step involves sampling from the conditional distribution
$$p\Big(C_{1:N}, h_{1:N}\Big|D, b^{1:M}, X_{1:N}^{1:M}, Y_{1:N}^{1:M}, Z_{1:N}^{1:M}, K^{1:M}, w_{1:N}^{1:P}\Big).$$
3. We update the variables $b^{1:M}, X_{1:N}^{1:M}, Y_{1:N}^{1:M}, Z_{1:N}^{1:M}, K^{1:M}$. This step involves sampling from the conditional distribution
$$p\Big(b^{1:M}, X_{1:N}^{1:M}, Y_{1:N}^{1:M}, Z_{1:N}^{1:M}, K^{1:M}\Big|C_{1:N}, h_{1:N}, D, w_{1:N}^{1:P}\Big).$$
4. We update the variables $C_{1:N}$ and $b^{1:M}, X_{1:N}^{1:M}, Y_{1:N}^{1:M}, Z_{1:N}^{1:M}, K^{1:M}$. This step involves sampling from the conditional distribution
$$p\Big(C_{1:N}, b^{1:M}, X_{1:N}^{1:M}, Y_{1:N}^{1:M}, Z_{1:N}^{1:M}, K^{1:M}\Big|h_{1:N}, D, w_{1:N}^{1:P}\Big).$$
5. We update the variables $C_{1:N}$ and $X_{1:N}^{1:M}, Y_{1:N}^{1:M}, Z_{1:N}^{1:M}, K^{1:M}$. This step involves sampling from the conditional distribution
$$p\Big(C_{1:N}, X_{1:N}^{1:M}, Y_{1:N}^{1:M}, Z_{1:N}^{1:M}, K^{1:M}\Big|h_{1:N}, b^{1:M}, D, w_{1:N}^{1:P}\Big).$$
6. We update the variables $X_{1:N}^{1:M}, Y_{1:N}^{1:M}, Z_{1:N}^{1:M}, K^{1:M}$. This step involves sampling from the conditional distribution
$$p\Big(X_{1:N}^{1:M}, Y_{1:N}^{1:M}, Z_{1:N}^{1:M}, K^{1:M}\Big|C_{1:N}, h_{1:N}, b^{1:M}, D, w_{1:N}^{1:P}\Big).$$

For all steps, we use customized Metropolis-Hastings samplers [14–17]. Our samplers are purpose-built and use techniques based on slice-sampling [56–58], or multiplicative random walks [59]. We evaluate our posterior with a fixed and finite total number of model emitters $M \gg 1$. As we explain in appendix D.8.1, this introduces an approximation in our analysis with an insignificant error [51, 60].

Although our computational scheme is mathematically valid, its performance can be improved considerably if it is combined with simulated annealing [61–64]. Annealing can speed up the convergence rate, reducing processing time, which may be slow for image data of typical sizes. In our sampler, we introduce an annealing factor $F^{(i)}$ and implement simulated annealing by replacing the EMCCD's excess noise factor f in our likelihood by the product $F^{(i)} f$. The annealing factor $F^{(i)} \geq 1$ is set to a large value at the beginning of our Gibbs iterations and gradually reduces to 1, for instance, such as in
$$F^{(i)} = 1 + (F^{(0)} - 1) \max\left(0, 1 - i/i_{\mathrm{ref}}\right)^2$$
where $F^{(0)}$ indicates the initial value, i_{ref} the reduction speed, and i is the iteration number.

D.10.2 Interpretation

Once a sequence of posterior samples $\theta^{(1)}, \theta^{(2)}, \dots$ is obtained by the successive repetition of the Gibbs sampling scheme, we may compute any statistic directly related to the sampled variables [13–17].

As with every Markov chain Monte Carlo method [13–17], the accuracy of the computed statistics is drastically improved if, from the entire chain of posterior samples, we first discard an initial burn-in portion. To identify this portion, we use batching [15]. Specifically, once simulated annealing terminates at iteration $i = i_{\text{ref}}$, we divide the remaining sequence into three batches, each covering 1/3 of successive samples in the chain. For each of the last two batches, we compute the statistics of interest and compare them. If these agree, we use either of them as our estimate; however, if they do not agree, we expand our initial chain by computing additional posterior samples and repeat until the statistics of the terminal two batches match.

Similarly, we may also compute statistics of interest of derived quantities that depend upon the sampled variables [15, 17]. One such quantity is the total number B of light emitters that contribute photons to the measured images. Since B depends upon the sampled indicators $b^{1:M}$, its posterior samples are readily obtained by the sum $B = \sum_{m=1}^{M} b^m$.

Unlike B, which is uniquely determined by the sampled variables, statistics that are sensitive to the labeling of the emitters, i.e. statistics that require distinctive labels m assigned to each model emitter, may not be determined uniquely by the sampled variables. This is a common characteristic shared by Bayesian nonparametric methods [65–67] and reflects the fact that both a priori and a posteriori, all emitters are equivalent in the sense that there is no preference for a particular labeling out of all $M!$ possible ones. For this reason, following our Markov chain Monte Carlo computations, we relabel the emitters in our posterior samples so that they maintain fixed labels. Essentially, from the $M!$ equivalent posterior modes that our posterior allows for, we chose methodologically only one to base our estimates upon with our relabeling approach. We explain our relabeling strategy below.

In any posterior sample θ, the variables requiring relabeling are $b^{1:M}, X_{1:N}^{1:M}, Y_{1:N}^{1:M}, Z_{1:N}^{1:M}, K^{1:M}$. For clarity, we denote their relabeled counterparts with $\dot{\theta}$ and $\dot{b}^{1:M}, \dot{X}_{1:N}^{1:M}, \dot{Y}_{1:N}^{1:M}, \dot{Z}_{1:N}^{1:M}, \dot{K}^{1:M}$, respectively. To obtain these, we first choose a pivot sample out of the computed ones $\theta^{(i)}$ that remain after burn-in removal. Our pivot is the posterior sample corresponding to the highest posterior $p(\theta|W)$. For clarity, we denote the pivot with $\tilde{\theta}$ and its variables with $\tilde{b}^{1:M}, \tilde{X}_{1:N}^{1:M}, \tilde{Y}_{1:N}^{1:M}, \tilde{Z}_{1:N}^{1:M}, \tilde{K}^{1:M}$. Subsequently, for each available $\theta^{(i)}$ we form all $M!$ possible samples $\ddot{\theta}_k^{(i)}$ through the permutations of the labels and for $\dot{\theta}^{(i)}$ we select the sample $\ddot{\theta}_k^{(i)}$ that is most similar to the pivot.

Our comparison with the pivot is based on the similarity metric

$$\mathcal{D}\left(\ddot{\theta}; \tilde{\theta}\right) = \sum_{m=1}^{M} \mathcal{R}_{\ddot{g}}\left(\ddot{b}^m, \ddot{X}_{1:N}^m, \ddot{Y}_{1:N}^m, \ddot{Z}_{1:N}^m; \tilde{b}^m, \tilde{X}_{1:N}^m, \tilde{Y}_{1:N}^m, \tilde{Z}_{1:N}^m\right)$$

which, in turn, depends additively on the metric

$$\mathcal{R}_{\ddot{g}}\left(\ddot{b}, \ddot{X}_{1:N}, \ddot{Y}_{1:N}, \ddot{Z}_{1:N}; \tilde{b}, \tilde{X}_{1:N}, \tilde{Y}_{1:N}, \tilde{Z}_{1:N}\right) = \sum_{p=1}^{P}\sum_{n=1}^{N} \tau_n^{\text{exps}} h_n \left| \ddot{b}\, Q_{\ddot{g}}^p(\ddot{X}_n, \ddot{Y}_n, \ddot{Z}_n) - \tilde{b}\, Q_{\ddot{g}}^p(\tilde{X}_n, \tilde{Y}_n, \tilde{Z}_n) \right|,$$

that compares $\ddot{\theta}$ and $\tilde{\theta}$ emitterwise based on the respective images. From these two, the former metric compares the entire population of emitters, while the latter metric compares individual emitters. Because the population metric, $\mathcal{D}\left(\ddot{\theta}; \tilde{\theta}\right)$ depends additively on the emitter metric

$$\mathcal{R}_{\ddot{g}}\left(\ddot{b}, \ddot{X}_{1:N}, \ddot{Y}_{1:N}, \ddot{Z}_{1:N}; \tilde{b}, \tilde{X}_{1:N}, \tilde{Y}_{1:N}, \tilde{Z}_{1:N}\right),$$

selecting the optimum $\dot{\theta}^{(i)}$ out of $\ddot{\theta}^{(i)}_{1:M!}$ reduces to a linear assignment problem that can be solved efficiently [68, 69], for instance through the Hungarian algorithm, without explicitly forming the permutations $\ddot{\theta}^{(i)}_{1:M!}$, which is impractical.

D.11 Calibration

D.12 Camera read-out

For cameras of the EMCCD type, agreement with the signal-to-noise specifications [48, 70, 71] requires a fixed excess noise factor $f = 2$/photon. However, the other camera parameters, e.g. μ, υ and ξ, are device and configuration dependent [71]. For this reason, their values must be calibrated separately for each camera and imaging configuration adopted. Below, we describe a standard calibration procedure.

The values of readout offset μ, and variance υ can be evaluated with dark images, i.e. images under no photon flux such as those obtained with the camera's shutter closed [70, 71]. We denote with $\omega_{1:N}^{0,1:P}$ such recorded images. Specifically, for these $u_n^{0,p} = 0$, and so our model reduces to

$$\omega_n^{0,p} \sim \text{Normal}\left(\mu, \upsilon\right).$$

For a sufficiently large number of exposures N, offset μ and variance υ can be recovered by the sample mean and variance, respectively

$$\mu = \frac{1}{NP}\sum_{n=1}^{N}\sum_{p=1}^{P}\omega_n^{0,p}, \qquad \upsilon = \frac{1}{NP-1}\sum_{n=1}^{N}\sum_{p=1}^{P}\left(\omega_n^{0,p} - \mu\right)^2.$$

The value of the gain ξ can be evaluated based on static images, i.e. images under constant photon flux such as those obtained when imaging an illuminated empty sample in plain buffer or other optically homogenous media. We denote with $\omega_{1:N}^{k,1:P}$ such recorded images, and use super-scripts $k = 1, \dots, K$ to denote different illumination levels. Specifically, for such images $u_n^{k,p} = C_{\text{static}}^{p,k} A^p \tau_n^{\text{exps}}$ where $C_{\text{static}}^{p,k}$ is the photon flux achieved at the kth illumination level, and so our model reduces to

$$\omega_n^{k,p} \sim \text{Normal}\left(\mu + \xi u_n^{k,p}, \upsilon + f\xi^2 u_n^{k,p}\right)$$

Accordingly, for each illumination level, each pixel's recordings mean and variance across exposures are given by $\mu^{k,p} = u_n^{k,p}\xi + \mu$ and $\upsilon^{k,p} = u_n^{k,p} f\xi^2 + \upsilon$, respectively. Consequently, they are related to each other by $\upsilon^{k,p} - \upsilon = f\xi\left(\mu^{k,p} - \mu\right)$. This relation can be used to obtain the value of ξ through least squares estimation [14, 72]. The result is

$$\xi = \frac{1}{f}\frac{\sum_{k=1}^{K}\sum_{n=1}^{N}\left(\upsilon^{k,p} - \upsilon\right)\left(\mu^{k,p} - \mu\right)}{\sum_{k=1}^{K}\sum_{n=1}^{N}\left(\mu^{k,p} - \mu\right)^2},$$

where the mean and variance of each pixel's recordings are recovered by the sample mean and variance, respectively

$$\mu^{k,p} = \frac{1}{N}\sum_{n=1}^{N}\omega_n^{k,p}, \qquad \upsilon^{k,p} = \frac{1}{N-1}\sum_{n=1}^{N}\left(\omega_n^{k,p} - \mu\right)^2.$$

D.12.1 Point spread function and illumination profile

For the calibration of the point spread function, suspended fiducial markers [73] can be imaged at multiple stages as graphically illustrated in Fig. D.5. For clarity, we denote with $\omega_{1:N}^{*,1:P}$ the images obtained with corresponding stage displacement $d_{1:N}^{\text{stg}}$ and, to proceed, we denote with $\bar{X}^m, \bar{Y}^m, \bar{Z}^m$ the emitters' positions in a laboratory's frame of reference. Further, we assume that the latter is oriented such that when the stage

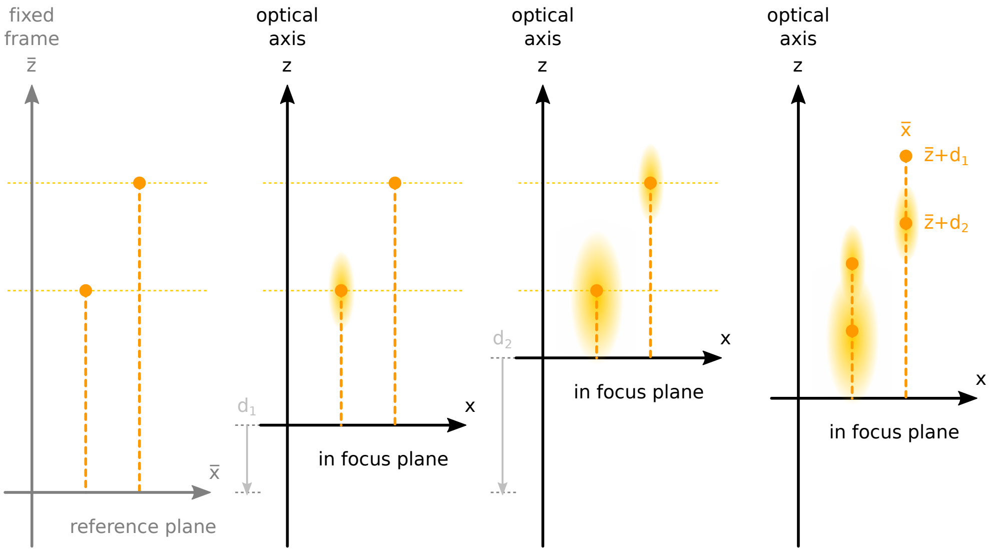

Figure D.5: Configuration of sample space and stage displacement used to calibrate the point spread function. We depict only two of the three spatial coordinates to increase clarity here. Suspended fiducial markers, immobile concerning the laboratory's frame of reference (left panel), are imaged at different stages (middle panels). Image analysis allows characterizing and reconstructing the point spread function along all spatial dimensions (right panel).

is displaced by d, the emitters are imaged at $X^m = \bar{X}^m, Y^m = \bar{Y}^m, Z^m = \bar{Z}^m + d$ concerning the object plane which is our common frame of reference. Therefore, in the nth image, the emitters are located at $X_n^m = \bar{X}^m, Y_n^m = \bar{Y}^m, Z_n^m = \bar{Z}^m + d_n^{\text{stg}}$. We illustrate this convention in Fig. D.5.

Assuming no emitter motion, the average number of incident photons in our calibration model reduces to

$$u_n^p = \tau_n^{\text{exps}} \left(C_n A^p + h \sum_{m=1}^{M} b^m \iint_{x_{\min}^p, y_{\min}^p}^{x_{\max}^p, y_{\max}^p} dxdy \; G_g(x, y; X_n^m, Y_n^m, Z_n^m) \right).$$

Under these assumptions, image analysis for the calibration of the point spread function can be performed with the model

$$\begin{aligned} g_j &\sim \text{InvGamma}\left(\alpha_g, \alpha_g - 1\right), & j &= 1, 2 \\ C_n &\sim \text{Gamma}\left(\alpha_C, C_{\text{ref}}/\alpha_C\right), & n &= 1, \dots, N \\ h &\sim \text{Gamma}\left(\alpha_h, h_{\text{ref}}/\alpha_h\right) & & \\ b^m &\sim \text{Bernoulli}\left(\gamma/M\right), & m &= 1, \dots, M \\ \bar{X}^m &\sim \text{Uniform}_{[X_{\min}, X_{\max}]}, & m &= 1, \dots, M \\ \bar{Y}^m &\sim \text{Uniform}_{[Y_{\min}, Y_{\max}]}, & m &= 1, \dots, M \\ \bar{Z}^m &\sim \text{Uniform}_{[Z_{\min}, Z_{\max}]}, & m &= 1, \dots, M \\ \omega_n^{*,p} | g_{1:3}, C_n, h, b^{1:M}, \bar{X}^{1:M}, \bar{Y}^{1:M}, \bar{Z}^{1:M} &\sim \text{Normal}\left(\mu + \xi u_n^p, \upsilon + f \xi^2 u_n^p\right), & n &= 1, \dots, N, \quad p = 1, \dots, P \end{aligned}$$

which yields estimates for g_1 and g_2. The estimation relies on the marginal posterior $p(g_1, g_2 | \omega_{1:N}^{*,1:P})$ which can be evaluated through Markov chain Monte Carlo sampling similar to the scheme we described in appendix D.10.

References

1. De Chaumont, F. *et al.* Icy: an open bioimage informatics platform for extended reproducible research. *Nat. Methods* **9,** 690 (2012).
2. Chenouard, N. *et al.* Objective comparison of particle tracking methods. *Nat. Methods* **11,** 281 (2014).
3. Abramowitz, M. & Stegun, I. A. *Handbook of mathematical functions with formulas, graphs, and mathematical tables* (US Government printing office, 1948).
4. Jaqaman, K. *et al.* Robust single-particle tracking in live-cell time-lapse sequences. *Nat. Methods* **5,** 695. ISSN: 1548-7105. `https://doi.org/10.1038/nmeth.1237` (2008).
5. Kilic, Z., Sgouralis, I. & Pressé, S. Generalizing HMMs to Continuous Time for Fast Kinetics: Hidden Markov Jump Processes. *Biophys. J.* **120,** 409. ISSN: 0006-3495. `https://www.sciencedirect.com/science/article/pii/S0006349521000060` (2021).
6. Heermann, T., Steiert, F., Ramm, B., Hundt, N. & Schwille, P. Mass-sensitive particle tracking to elucidate the membrane-associated MinDE reaction cycle. *Nat. Methods* **18,** 1239 (2021).
7. Foley, E. D., Kushwah, M. S., Young, G. & Kukura, P. Mass photometry enables label-free tracking and mass measurement of single proteins on lipid bilayers. *Nat. Methods* **18,** 1247 (2021).
8. Masson, J.-B. *et al.* Mapping the Energy and Diffusion Landscapes of Membrane Proteins at the Cell Surface Using High-Density Single-Molecule Imaging and Bayesian Inference: Application to the Multiscale Dynamics of Glycine Receptors in the Neuronal Membrane. *Biophys. J.* **106,** 74. ISSN: 0006-3495. `https://www.sciencedirect.com/science/article/pii/S0006349513011946` (2014).
9. Borgia, A. *et al.* Extreme disorder in an ultrahigh-affinity protein complex. *Nature* **555,** 61 (2018).
10. Ronceray, N. *et al.* Liquid-activated quantum emission from pristine hexagonal boron nitride for nanofluidic sensing. *Nat. Mater.* **22,** 1236 (2023).
11. Bishop, C. M. *Pattern recognition and machine learning* (Springer, 2006).
12. Sivia, D. & Skilling, J. *Data analysis: a Bayesian tutorial* (OUP Oxford, 2006).
13. Von Toussaint, U. Bayesian inference in physics. *Rev. Mod. Phys.* **83,** 943. `https://link.aps.org/doi/10.1103/RevModPhys.83.943` (3 2011).
14. Gelman, A. *et al. Bayesian data analysis* 3rd (CRC press, 2013).
15. Robert, C. P., Casella, G. & Casella, G. *Introducing Monte Carlo methods with R* (Springer, 2010).
16. Robert, C. & Casella, G. *Monte Carlo statistical methods* (Springer Science & Business Media, 2013).
17. Liu, J. S. *Monte Carlo strategies in scientific computing* (Springer Science & Business Media, 2008).
18. Goodman, J. W. *Introduction to Fourier optics* (Roberts and Company Publishers, 2005).
19. Fowles, G. R. *Introduction to modern optics* (Courier Corporation, 1989).
20. Siegman, A. E. *Lasers* (University Science Books, 1986).
21. Born, M. & Wolf, E. *Principles of optics: electromagnetic theory of propagation, interference and diffraction of light* (Elsevier, 2013).
22. Kilic, Z. *et al.* Extraction of rapid kinetics from smFRET measurements using integrative detectors. *Cell Rep. Phys. Sci.* **2,** 100409. ISSN: 2666-3864. `https://www.sciencedirect.com/science/article/pii/S2666386421000990` (2021).
23. Lakowicz, J. R. *Principles of fluorescence spectroscopy* (Springer science & business media, 2013).
24. Saleh, B. E. & Teich, M. C. *Fundamentals of photonics* (john Wiley & sons, 2019).
25. Wartak, M. S. *Computational photonics: an introduction with MATLAB* (Cambridge University Press, 2013).

26. Voelz, D. G. *Computational Fourier optics: a MATLAB tutorial* (SPIE press Bellingham, Washington, 2011).

27. Aguet, F. *Super-Resolution Fluorescence Microscopy Based on Physical Models* EPFL Thesis no. 4418 (2009), 209 p. (Swiss Federal Institute of Technology Lausanne (EPFL), 2009).

28. Gibson, S. F. & Lanni, F. Experimental test of an analytical model of aberration in an oil-immersion objective lens used in three-dimensional light microscopy. *JOSA A* **8,** 1601. https://doi.org/10.1364/JOSAA.9.000154 (1991).

29. Tao, L. & Nicholson, C. The three-dimensional point spread functions of a microscope objective in image and object space. *J. Microsc.* **178,** 267. https://onlinelibrary.wiley.com/doi/abs/10.1111/j.1365-2818.1995.tb03604.x (1995).

30. Lee, A., Tsekouras, K., Calderon, C., Bustamante, C. & Pressé, S. Unraveling the Thousand Word Picture: An Introduction to Super-Resolution Data Analysis. *Chem. Rev.* **117,** 7276. https://doi.org/10.1021/acs.chemrev.6b00729 (2017).

31. Deschout, H., Neyts, K. & Braeckmans, K. The influence of movement on the localization precision of sub-resolution particles in fluorescence microscopy. *J. Biophotonics* **5,** 97 (2012).

32. Zhang, B., Zerubia, J. & Olivo-Marin, J.-C. Gaussian approximations of fluorescence microscope point-spread function models. *Appl. Opt.* **46,** 1819. http://ao.osa.org/abstract.cfm?URI=ao-46-10-1819 (2007).

33. Ball, D. W. *Field guide to spectroscopy* (SPIE Press Bellingham, Washington, 2006).

34. Hairer, E., Lubich, C. & Wanner, G. *Geometric numerical integration: structure-preserving algorithms for ordinary differential equations* (Springer Science & Business Media, 2006).

35. Schlick, T. *Molecular modeling and simulation: an interdisciplinary guide* (Springer Science & Business Media, 2010).

36. Atkinson, K. & Han, W. *Theoretical numerical analysis* (Springer, 2005).

37. Quarteroni, A., Sacco, R. & Saleri, F. *Numerical mathematics* (Springer Science & Business Media, 2010).

38. Pavliotis, G. A. *Stochastic processes and applications: diffusion processes, the Fokker-Planck and Langevin equations* (Springer, 2014).

39. Zwanzig, R. *Nonequilibrium statistical mechanics* (Oxford University Press, 2001).

40. Tavakoli, M. *et al.* Pitching Single-Focus Confocal Data Analysis One Photon at a Time with Bayesian Nonparametrics. *Phys. Rev. X* **10,** 011021. https://link.aps.org/doi/10.1103/PhysRevX.10.011021 (1 2020).

41. Jazani, S. *et al.* An alternative framework for fluorescence correlation spectroscopy. *Nat. Commun.* **10,** 3662. https://doi.org/10.1038/s41467-019-11574-2 (2019).

42. Jazani, S., Sgouralis, I. & Pressé, S. A method for single molecule tracking using a conventional single-focus confocal setup. *J. Chem. Phys.* **150,** 114108. https://doi.org/10.1063/1.5083869 (2019).

43. Berg, H. C. in *Random Walks in Biology* (Princeton University Press, 2018).

44. Qian, H., Sheetz, M. P. & Elson, E. L. Single particle tracking. Analysis of diffusion and flow in two-dimensional systems. *Biophys. J.* **60,** 910 (1991).

45. Hildebrand, F. B. *Introduction to numerical analysis* (Courier Corporation, 1987).

46. Press, W. H. *Numerical recipes 3rd edition: The art of scientific computing* (Cambridge university press, 2007).

47. Atkinson, K. *An introduction to numerical analysis* (John wiley & sons, 1991).

48. Hirsch, M., Wareham, R. J., Martin-Fernandez, M. L., Hobson, M. P. & Rolfe, D. J. A Stochastic Model for Electron Multiplication Charge-Coupled Devices – From Theory to Practice. *PLoS One* **8,** 1. https://doi.org/10.1371/journal.pone.0053671 (2013).

49. Bender, C. M., Orszag, S. & Orszag, S. A. *Advanced mathematical methods for scientists and engineers I: Asymptotic methods and perturbation theory* (Springer Science & Business Media, 1999).

50. Hinch, E. J. *Perturbation Methods* (Cambridge University Press, 1991).

51. Al Labadi, L. & Zarepour, M. On approximations of the beta process in latent feature models: Point processes approach. *Sankhya A* **80,** 59 (2018).

52. Cheng, Y., Li, D. & Jiang, W. The Exact Inference of Beta Process and Beta Bernoulli Process From Finite Observations. *Comput. Model. Eng. Sci.* **121,** 49 (2019).

53. Paisley, J., Blei, D. & Jordan, M. *Stick-breaking beta processes and the Poisson process* in *Artificial Intelligence and Statistics* (2012), 850.

54. Paisley, J. W., Zaas, A. K., Woods, C. W., Ginsburg, G. S. & Carin, L. *A stick-breaking construction of the beta process* in *ICML* (2010).

55. Paisley, J., Carin, L. & Blei, D. *Variational inference for stick-breaking beta process priors* in *Proceedings of the 28th international conference on machine learning* (2011).

56. Neal, R. M. Slice sampling. *Ann. Stat.* **31,** 705 (2003).

57. Nishihara, R., Murray, I. & Adams, R. P. Parallel MCMC with generalized elliptical slice sampling. *J. Mach. Learn. Res.* **15,** 2087 (2014).

58. Murray, I., Adams, R. & MacKay, D. *Elliptical slice sampling* in *Proceedings of the thirteenth international conference on artificial intelligence and statistics* (2010), 541.

59. Dutta, S. Multiplicative random walk Metropolis-Hastings on the real line. *Sankhya B* **74,** 315. ISSN: 0976-8394. https://doi.org/10.1007/s13571-012-0040-5 (2012).

60. Paisley, J. & Carin, L. *Nonparametric factor analysis with beta process priors* in *Proceedings of the 26th annual international conference on machine learning* (2009), 777.

61. Henderson, D., Jacobson, S. H. & Johnson, A. W. in *Handbook of metaheuristics* 287 (Springer, 2003).

62. Van Laarhoven, P. J. & Aarts, E. H. in *Simulated annealing: Theory and applications* 7 (Springer, 1987).

63. Bertsimas, D. & Tsitsiklis, J. Simulated annealing. *Statistical science* **8,** 10 (1993).

64. Hajek, B. Cooling schedules for optimal annealing. *Math. Oper. Res.* **13,** 311 (1988).

65. Puolamäki, K. & Kaski, S. *Bayesian solutions to the label switching problem* in *International Symposium on Intelligent Data Analysis* (2009), 381.

66. Rodríguez, C. E. & Walker, S. G. Label switching in Bayesian mixture models: Deterministic relabeling strategies. *Journal of Computational and Graphical Statistics* **23,** 25 (2014).

67. Stephens, M. Dealing with label switching in mixture models. *J. R. Stat. Soc., B: Stat. Methodol.* **62,** 795 (2000).

68. Duff, I. S. & Koster, J. On Algorithms For Permuting Large Entries to the Diagonal of a Sparse Matrix. *SIAM J. Matrix Anal. Appl.* **22,** 973. eprint: https://doi.org/10.1137/S0895479899358443. https://doi.org/10.1137/S0895479899358443 (2001).

69. Burkard, R., Dell'Amico, M. & Martello, S. *Assignment Problems* eprint: https://epubs.siam.org/doi/pdf/10.1137/1.9781611972238. https://epubs.siam.org/doi/abs/10.1137/1.9781611972238 (Society for Industrial and Applied Mathematics, 2012).

70. Huang, F. *et al.* Video-rate nanoscopy using sCMOS camera–specific single-molecule localization algorithms. *Nat. Methods* **10,** 653. ISSN: 1548-7105. https://doi.org/10.1038/nmeth.2488 (2013).

71. Harpsøe, K. B., Andersen, M. I. & Kjægaard, P. Bayesian photon counting with electron-multiplying charge coupled devices (EMCCDs). *Astron. Astrophys.* **537,** A50. `https://doi.org/10.1051/0004-6361/201117089` (2012).

72. Hastie, T., Tibshirani, R., Friedman, J. H. & Friedman, J. H. *The elements of statistical learning: data mining, inference, and prediction* (Springer, 2009).

73. Gell, C., Berndt, M., Enderlein, J. & Diez, S. TIRF microscopy evanescent field calibration using tilted fluorescent microtubules. *J. Microsc.* **234,** 38 (2009).
