# Supplementary material for: BNP-Track: a framework for superresolved tracking
Source: Nat Methods. 2024 Jul 22;21(9):1716–24. doi: 10.1038/s41592-024-02349-9 (PMC11399105; doi:10.1038/s41592-024-02349-9)
Supplement: Supplementary file 4 — Supplementary Data [file 41592_2024_2349_MOESM4_ESM.zip › Supplementary_Data/Supplementary_Data_1.pdf]

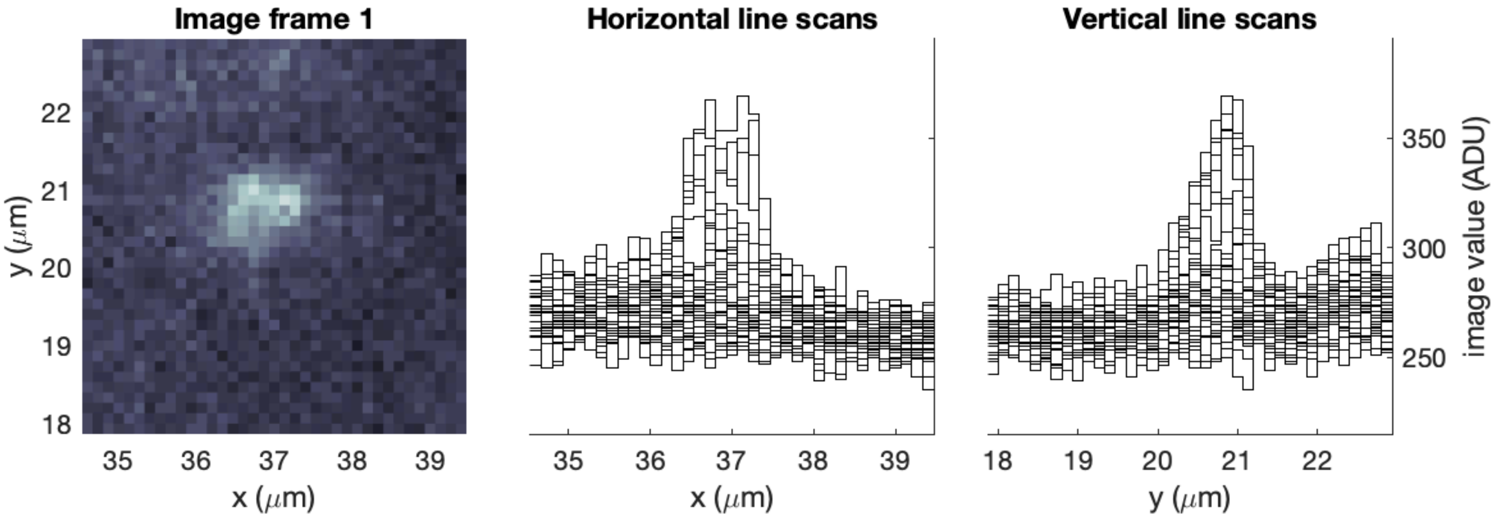

Image frame 1
Horizontal line scans
Vertical line scans
y (μm)
x (μm)
x (μm)
y (μm)
image value (ADU)
22
21
20
19
18
35
36
37
38
39
35
36
37
38
39
18
19
20
21
22
350
300
250

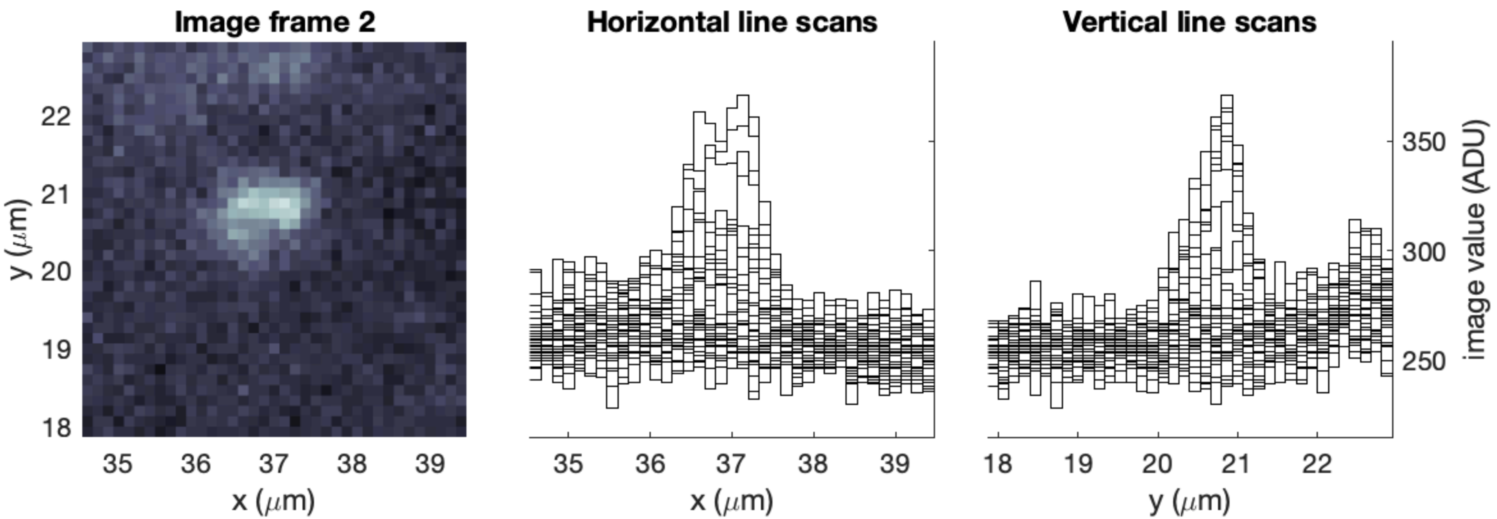

Image frame 2
Horizontal line scans
Vertical line scans
y (μm)
x (μm)
x (μm)
y (μm)
image value (ADU)
22
21
20
19
18
35
36
37
38
39
250
300
350

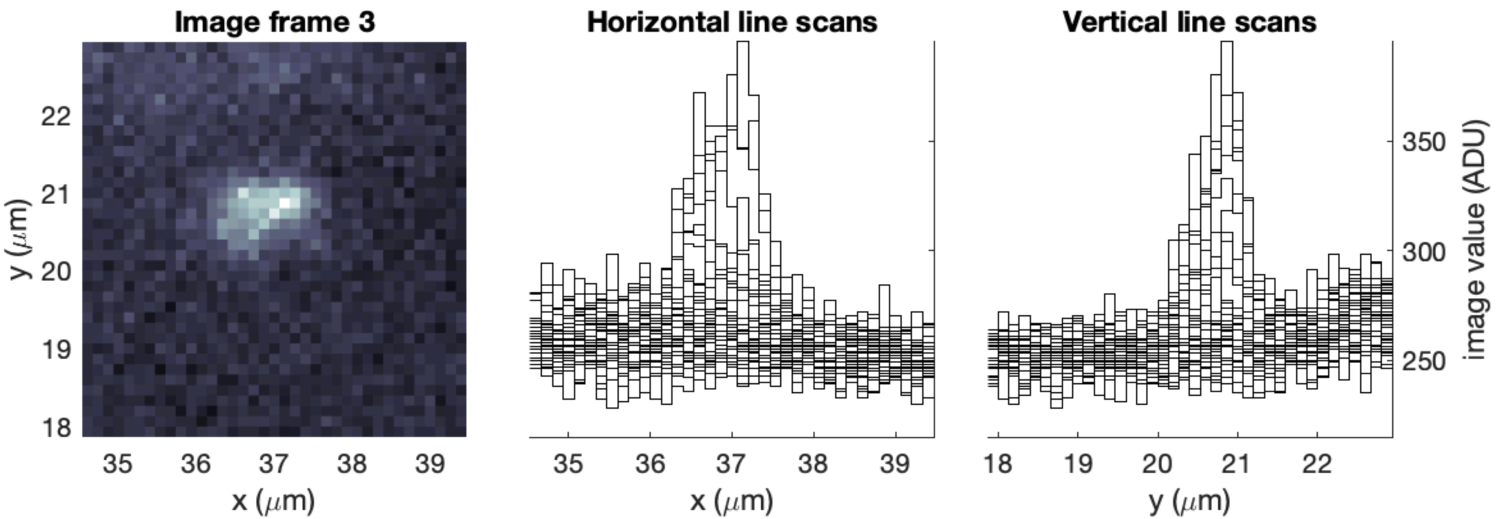

Image frame 3
Horizontal line scans
Vertical line scans
y (μm)
x (μm)
x (μm)
y (μm)
image value (ADU)
22
21
20
19
18
35
36
37
38
39
35
36
37
38
39
18
19
20
21
22
350
300
250

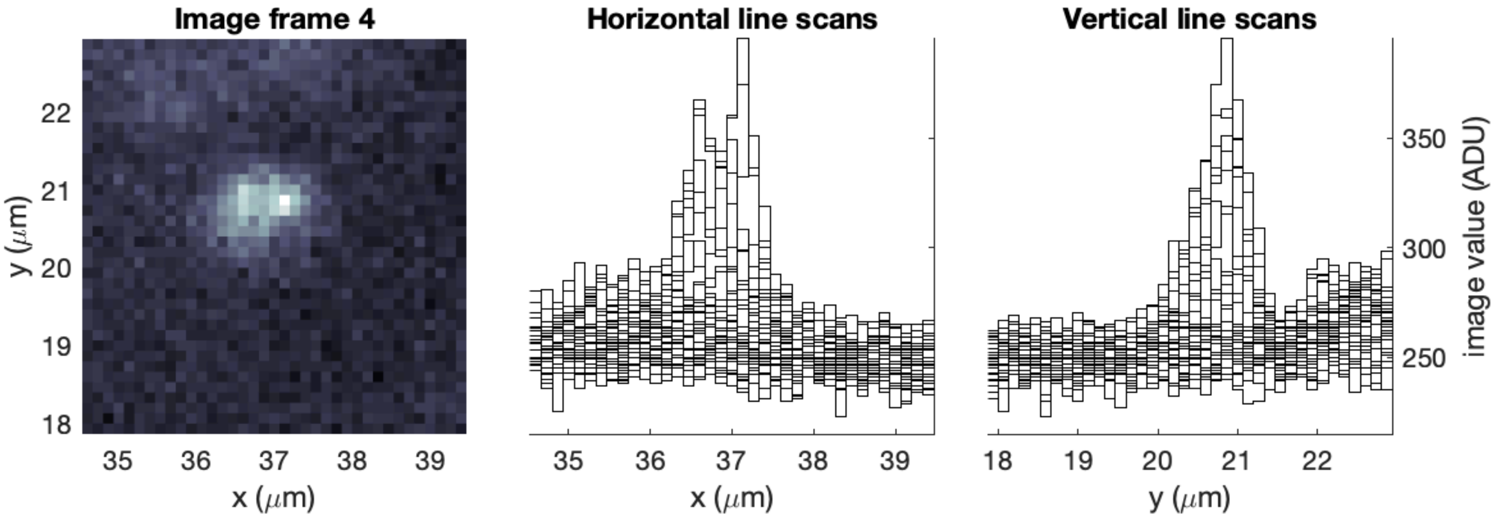

Image frame 4
Horizontal line scans
Vertical line scans
y (μm)
x (μm)
x (μm)
y (μm)
image value (ADU)
22
21
20
19
18
35
36
37
38
39
35
36
37
38
39
18
19
20
21
22
350
300
250

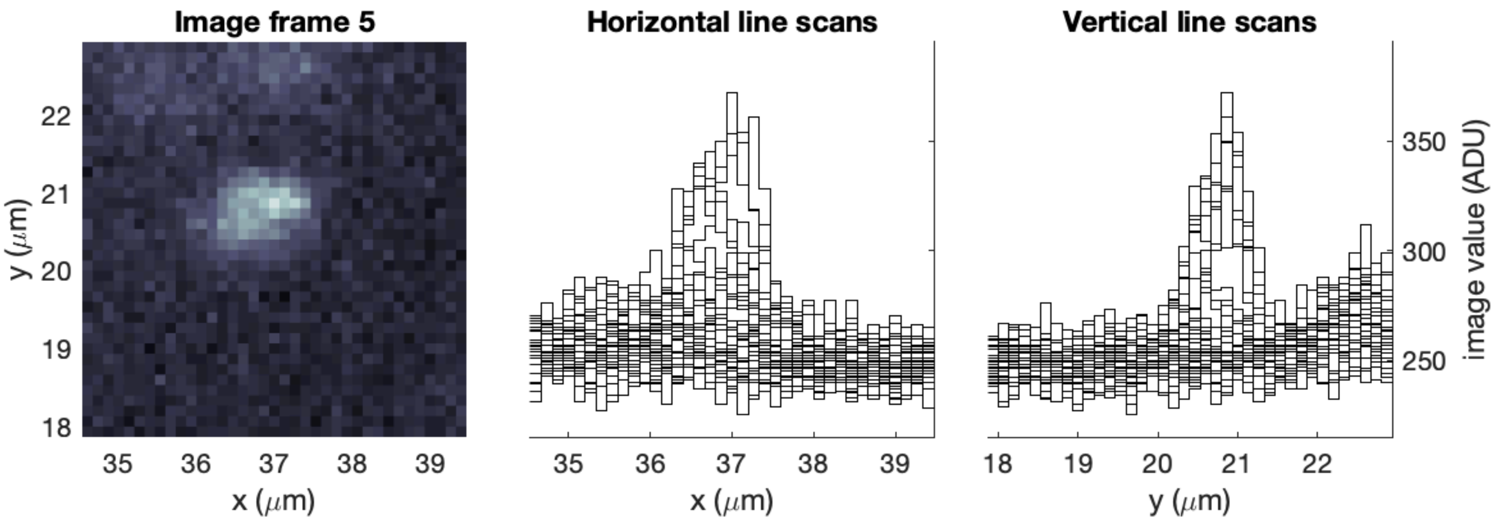

Image frame 5
Horizontal line scans
Vertical line scans
y (μm)
x (μm)
x (μm)
y (μm)
image value (ADU)
22
21
20
19
18
35
36
37
38
39
35
36
37
38
39
18
19
20
21
22
350
300
250

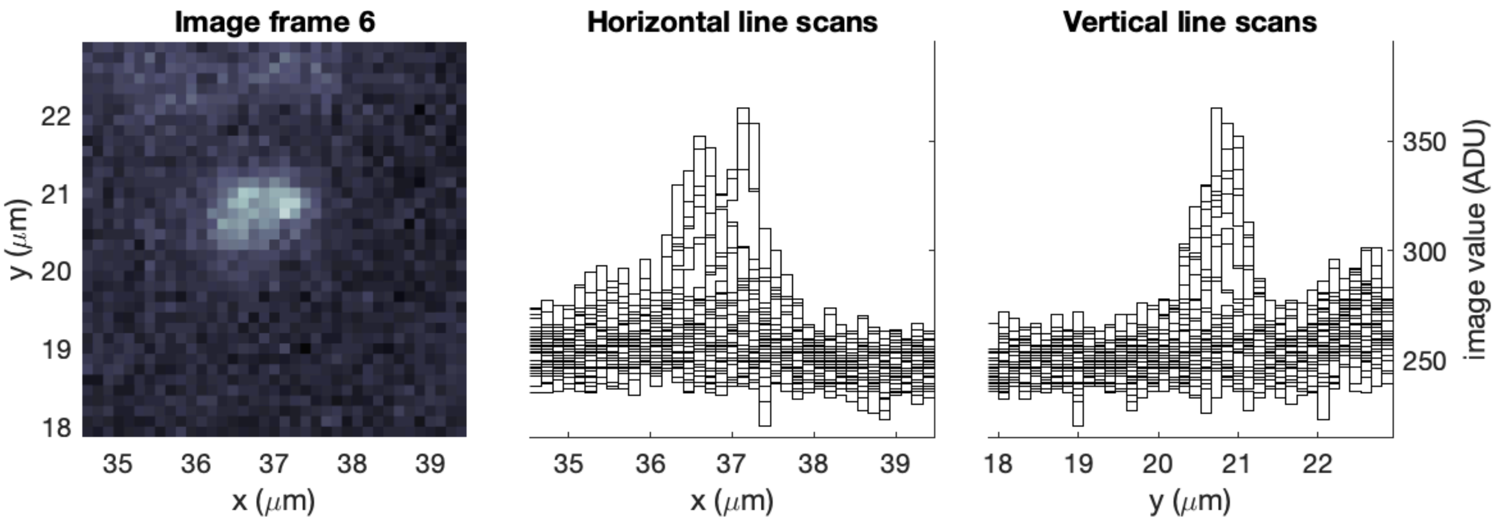
Image frame 6
Horizontal line scans
Vertical line scans
y (μm)
x (μm)
x (μm)
y (μm)
image value (ADU)
22
21
20
19
18
35
36
37
38
39
35
36
37
38
39
18
19
20
21
22
350
300
250

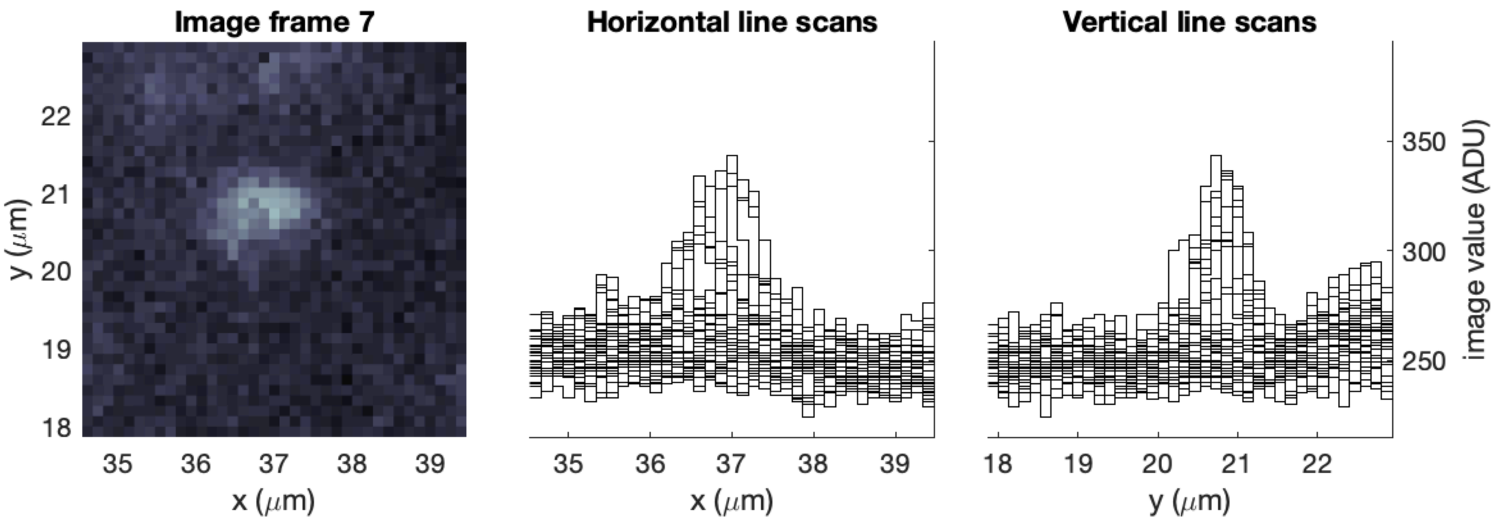
Image frame 7
Horizontal line scans
Vertical line scans
22
21
20
19
18
y (μm)
35
36
37
38
39
x (μm)
350
300
250
image value (ADU)
y (μm)

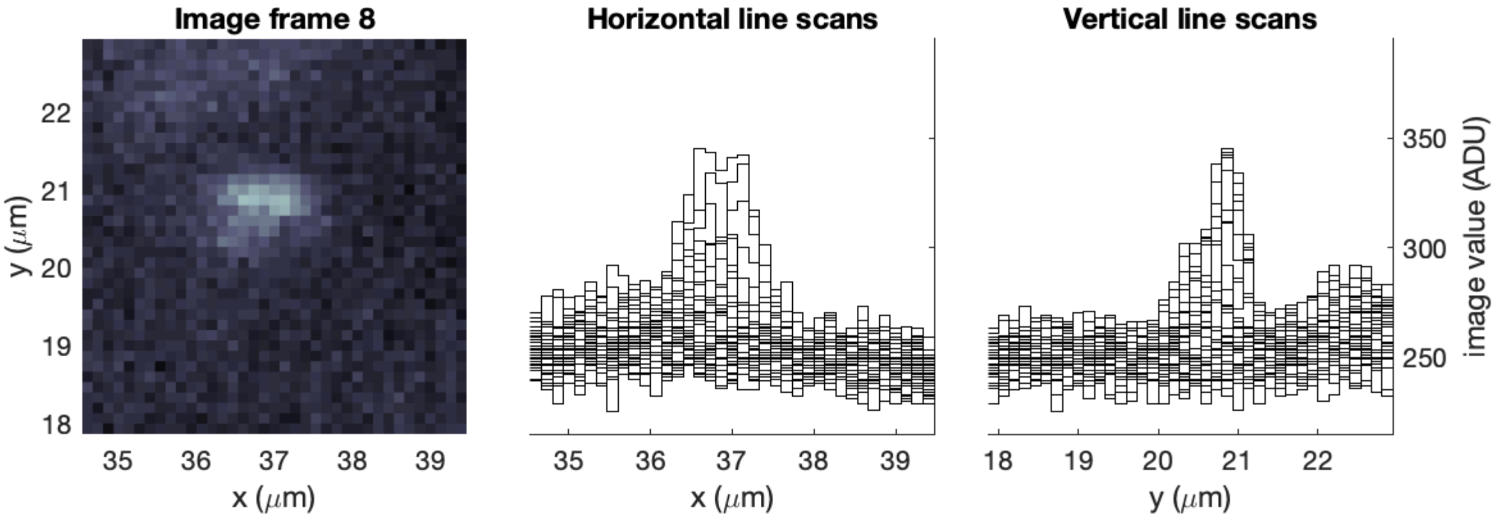

Image frame 8
Horizontal line scans
Vertical line scans
y (μm)
x (μm)
y (μm)
image value (ADU)
22
21
20
19
18
35
36
37
38
39
350
300
250

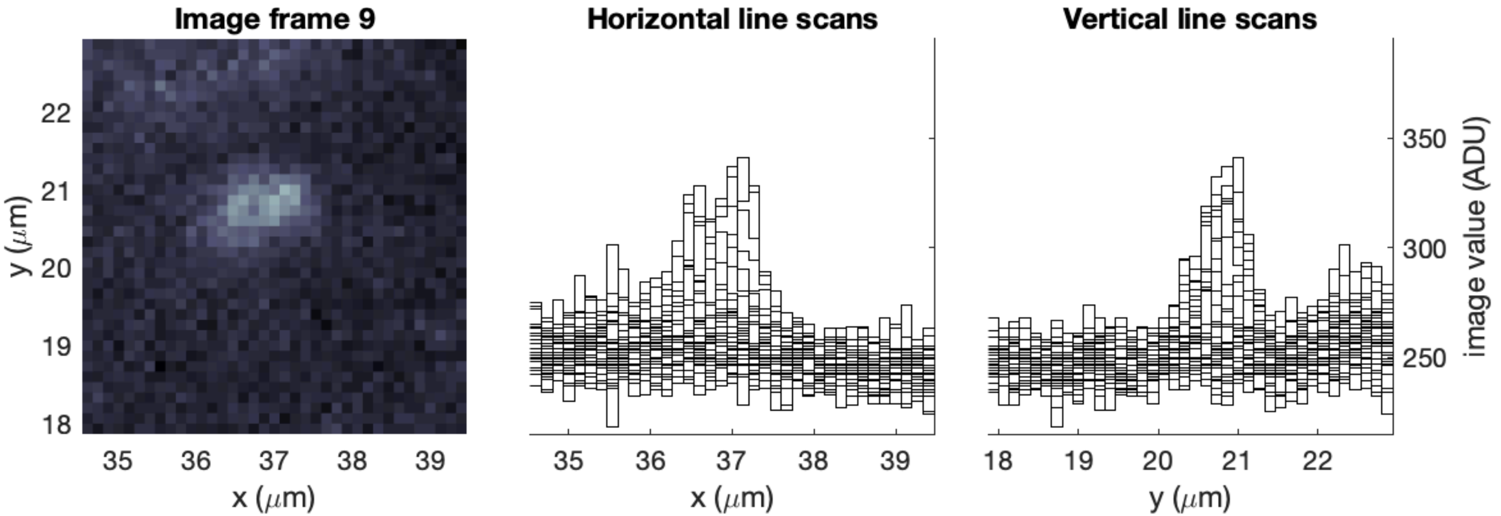

Image frame 9
Horizontal line scans
Vertical line scans
y (μm)
x (μm)
x (μm)
y (μm)
image value (ADU)
22
21
20
19
18
35
36
37
38
39
350
300
250

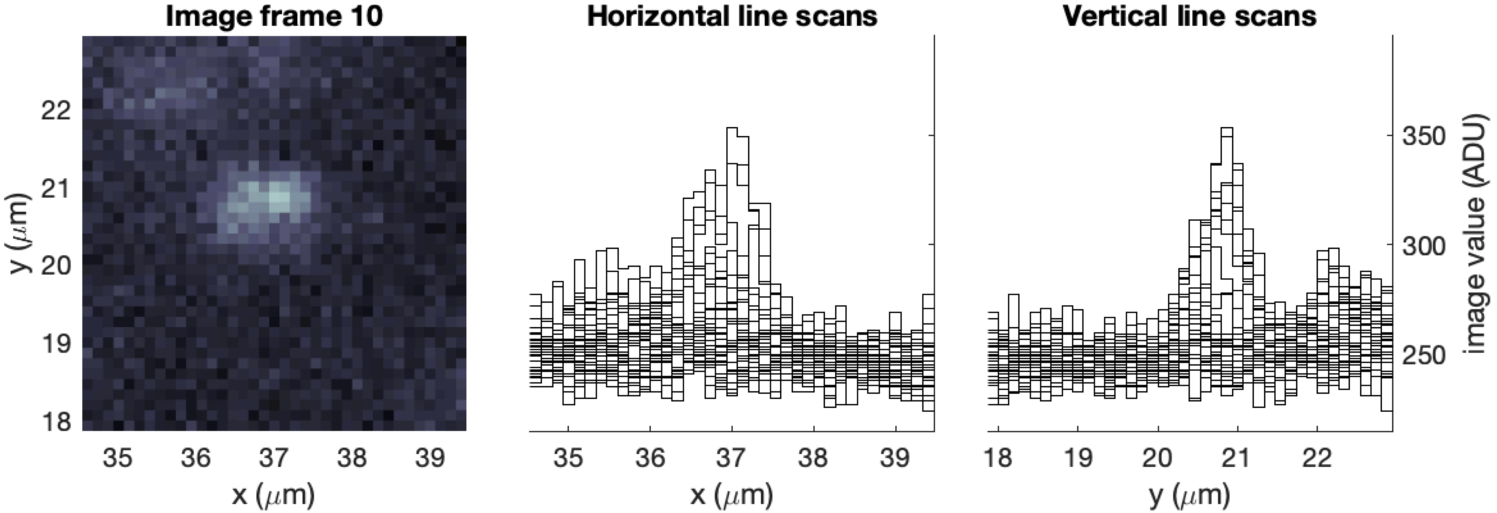
Image frame 10
Horizontal line scans
Vertical line scans
y (μm)
x (μm)
x (μm)
y (μm)
image value (ADU)
350
300
250
22
21
20
19
18
35
36
37
38
39
35
36
37
38
39
18
19
20
21
22

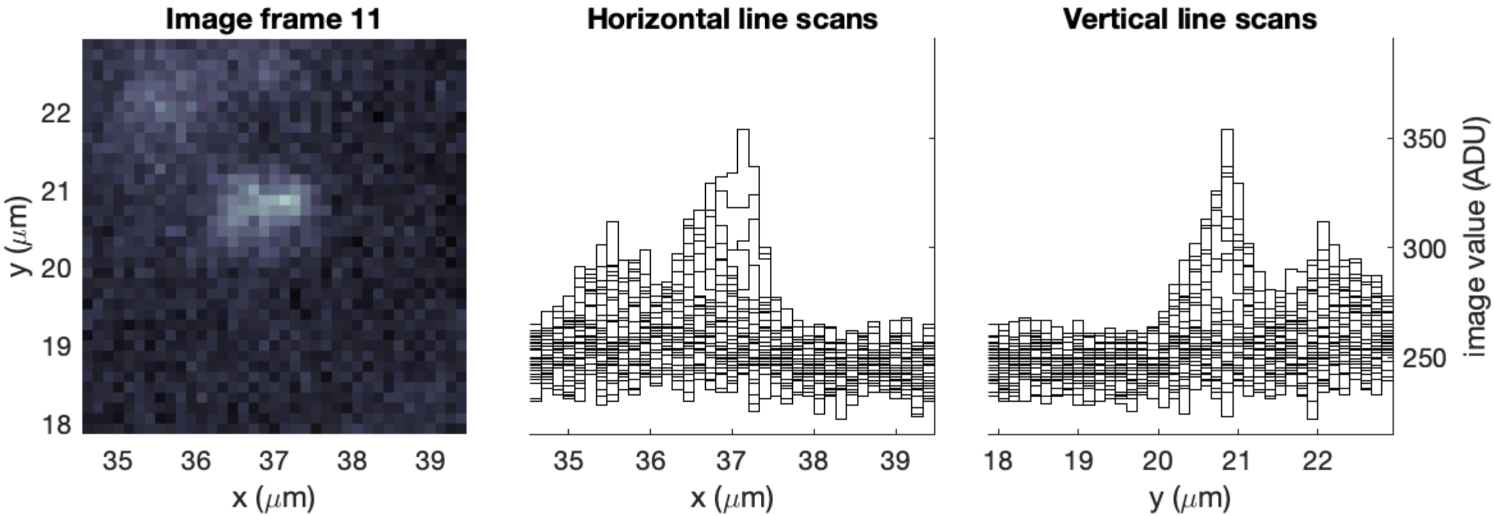
Image frame 11
Horizontal line scans
Vertical line scans
y (μm)
x (μm)
x (μm)
y (μm)
image value (ADU)
22
21
20
19
18
35
36
37
38
39
350
300
250

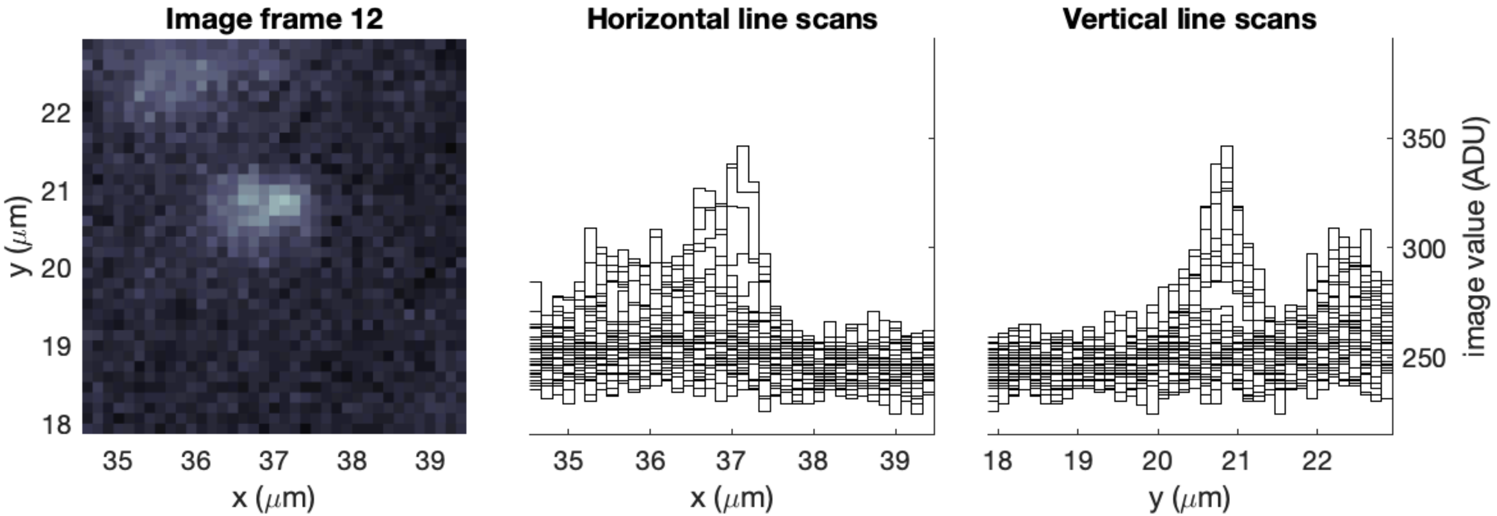

Image frame 12
Horizontal line scans
Vertical line scans
y (μm)
x (μm)
y (μm)
image value (ADU)
22
21
20
19
18
35
36
37
38
39
350
300
250

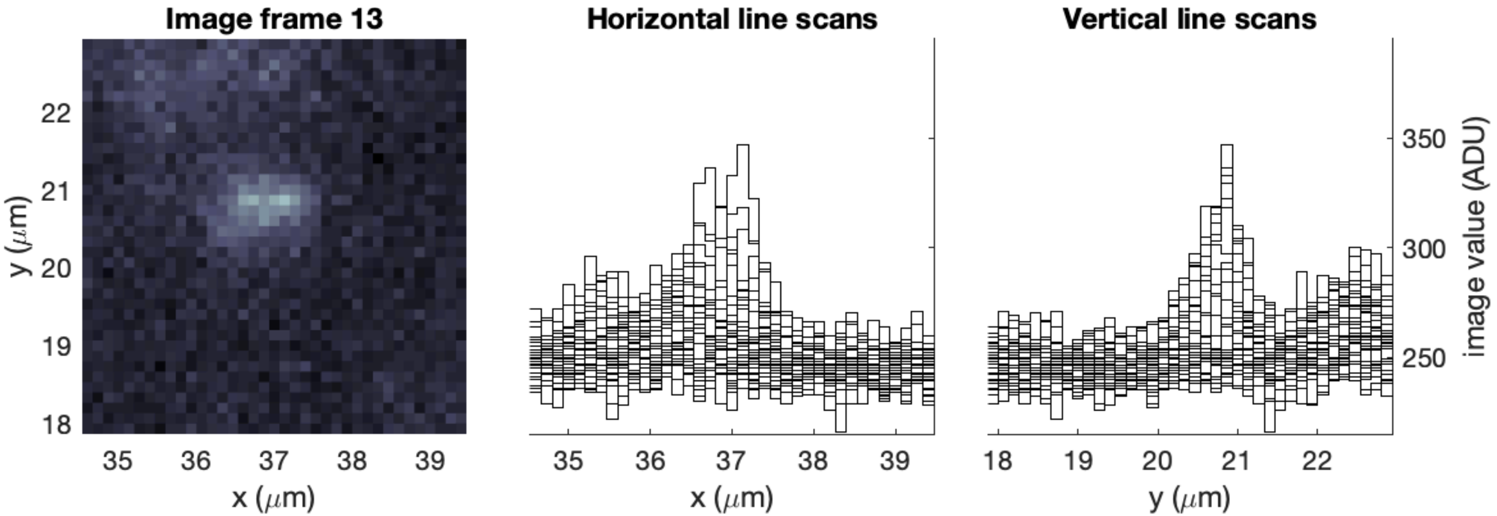

Image frame 13
Horizontal line scans
Vertical line scans
y (μm)
x (μm)
x (μm)
y (μm)
image value (ADU)
22
21
20
19
18
35
36
37
38
39
18
19
20
21
22
350
300
250

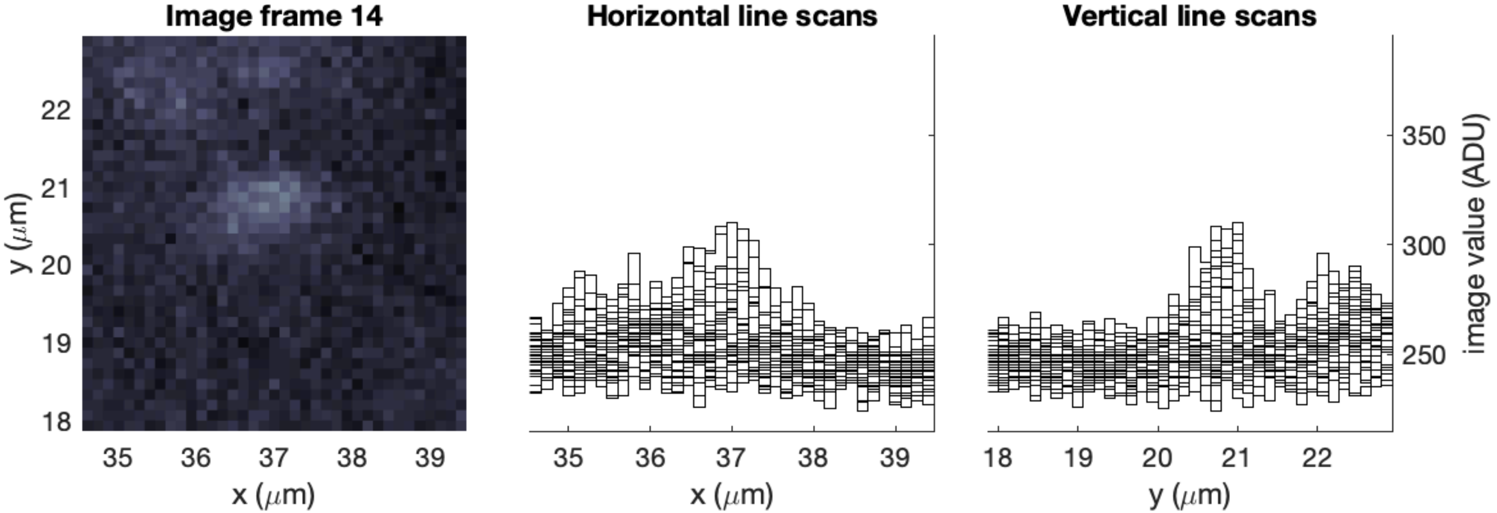

Image frame 14
Horizontal line scans
Vertical line scans
y (μm)
x (μm)
y (μm)
image value (ADU)

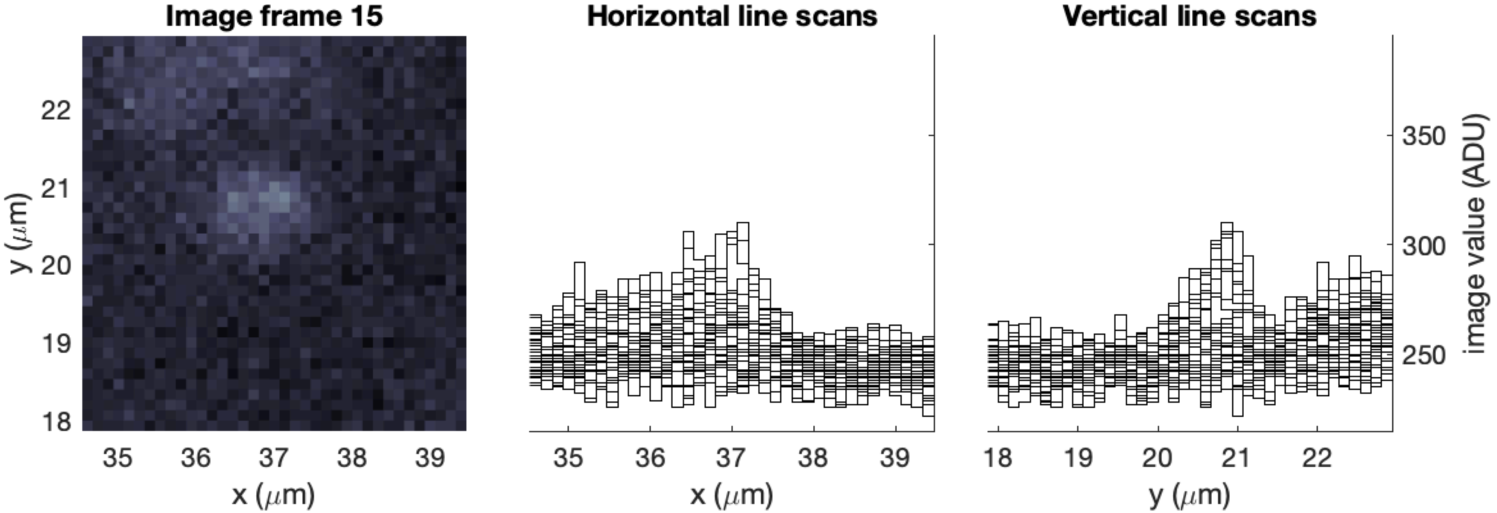
Image frame 15
Horizontal line scans
Vertical line scans
y (μm)
x (μm)
x (μm)
y (μm)
image value (ADU)
22
21
20
19
18
35
36
37
38
39
35
36
37
38
39
18
19
20
21
22
350
300
250

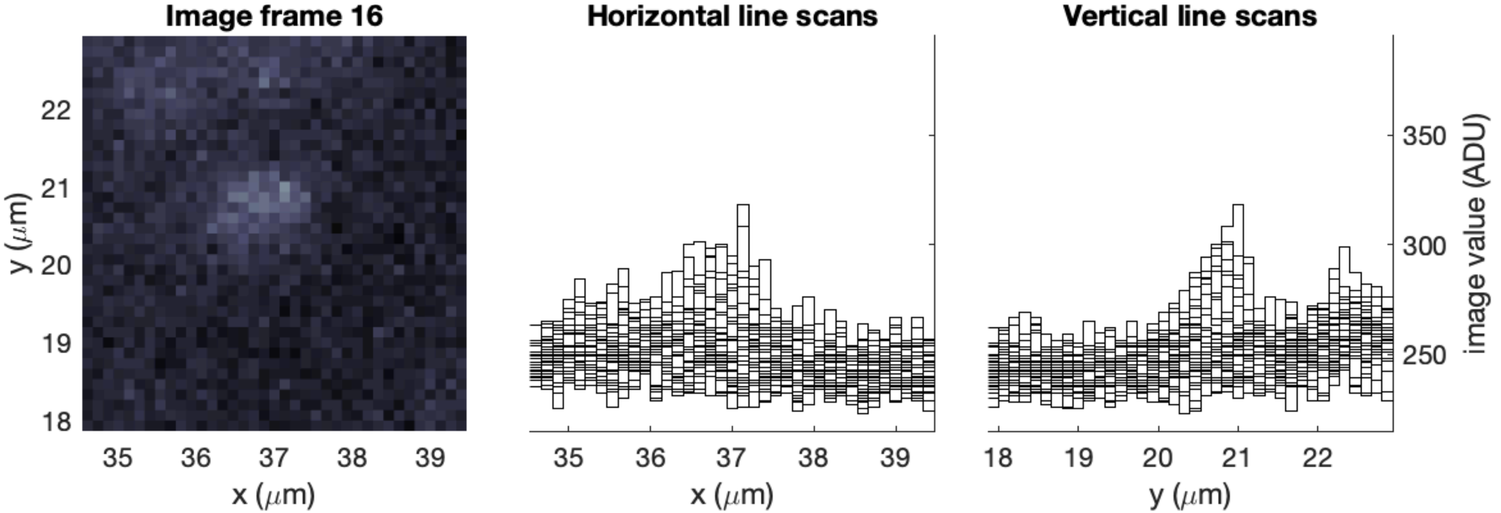

Image frame 16
Horizontal line scans
Vertical line scans
y (μm)
x (μm)
y (μm)
image value (ADU)
22
21
20
19
18
35
36
37
38
39
350
300
250

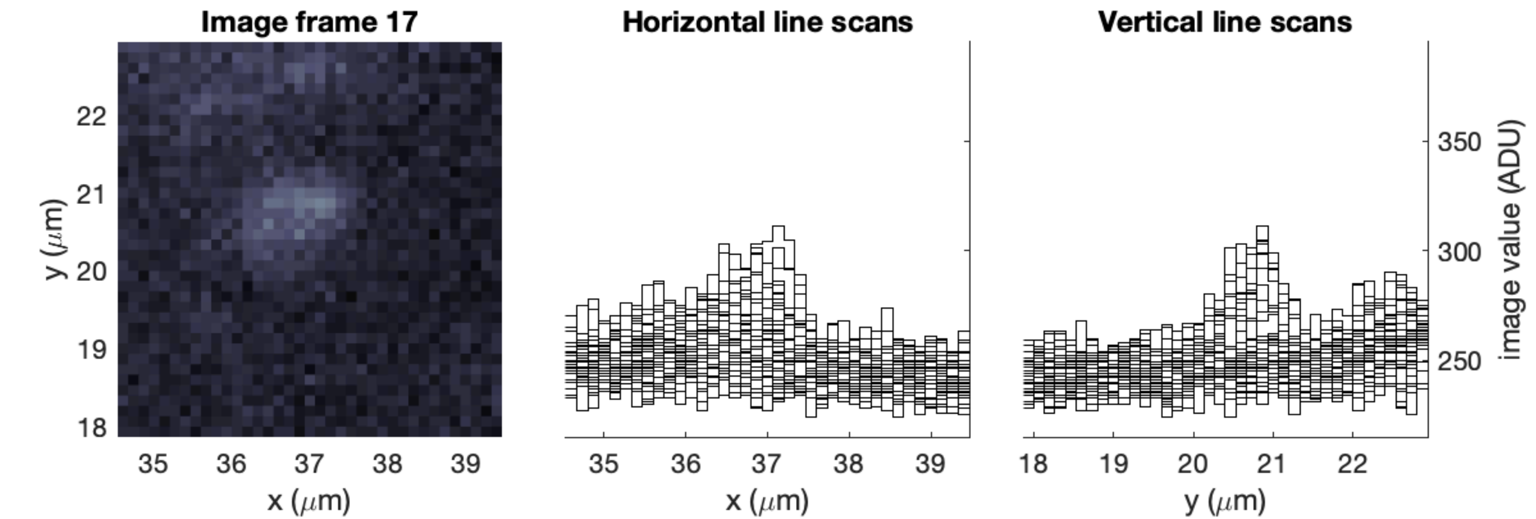

Image frame 17
Horizontal line scans
Vertical line scans
y (μm)
x (μm)
x (μm)
y (μm)
image value (ADU)
22
21
20
19
18
35
36
37
38
39
350
300
250

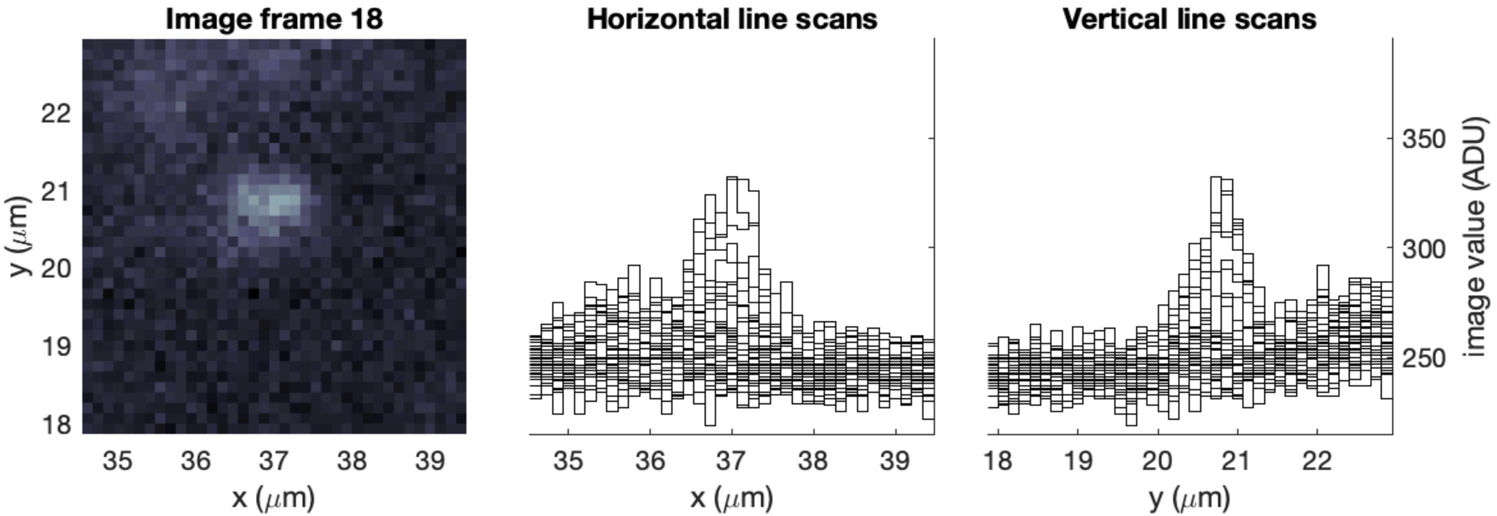

Image frame 18
Horizontal line scans
Vertical line scans
y (μm)
x (μm)
x (μm)
y (μm)
image value (ADU)
22
21
20
19
18
35
36
37
38
39
350
300
250

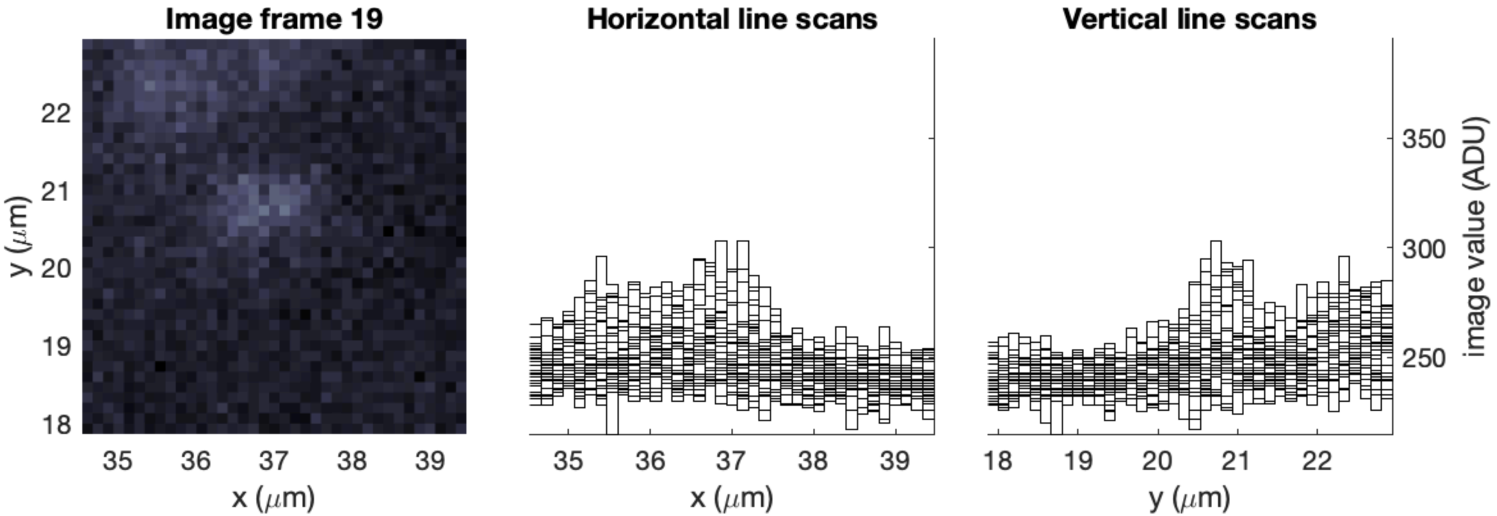

Image frame 19
Horizontal line scans
Vertical line scans
y (μm)
x (μm)
x (μm)
y (μm)
image value (ADU)
22
21
20
19
18
35
36
37
38
39
35
36
37
38
39
18
19
20
21
22
350
300
250

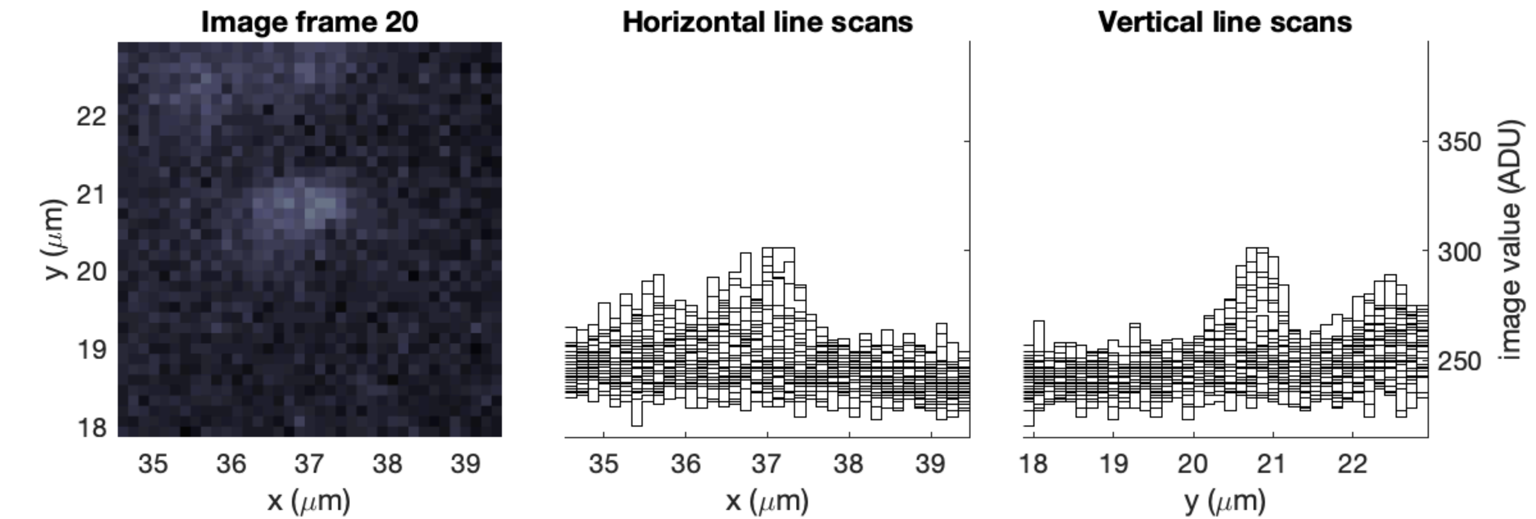

Image frame 20
Horizontal line scans
Vertical line scans
y (μm)
x (μm)
x (μm)
y (μm)
image value (ADU)
22
21
20
19
18
35
36
37
38
39
350
300
250

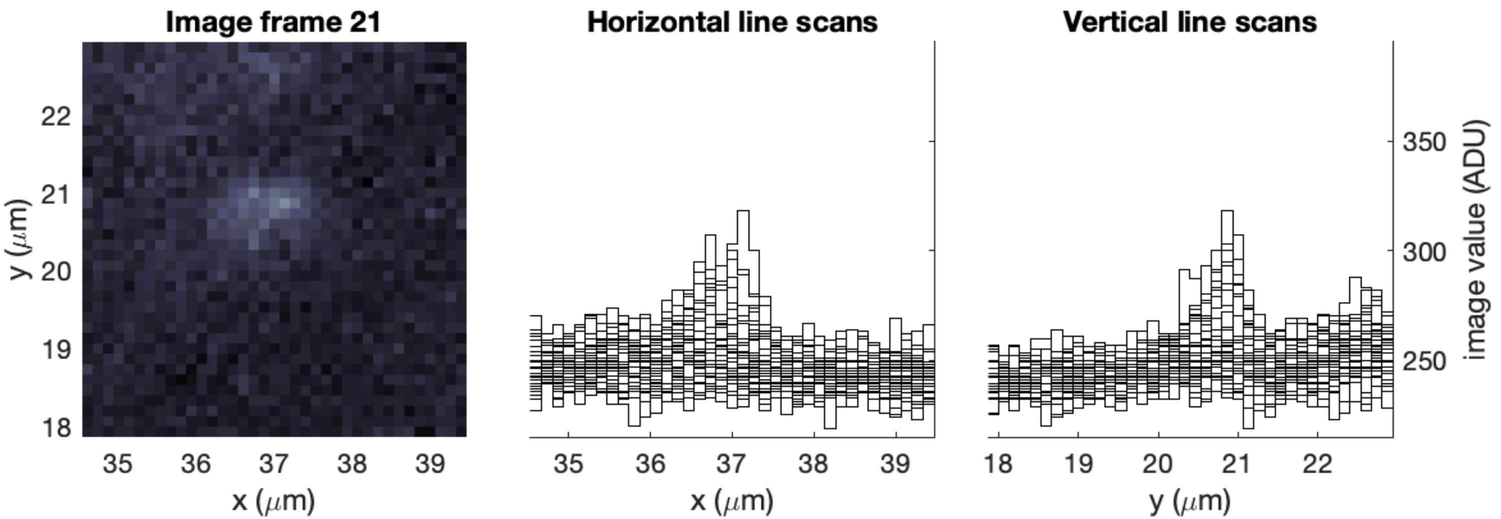
Image frame 21
Horizontal line scans
Vertical line scans
y (μm)
22
21
20
19
18
x (μm)
35
36
37
38
39
y (μm)
image value (ADU)
350
300
250

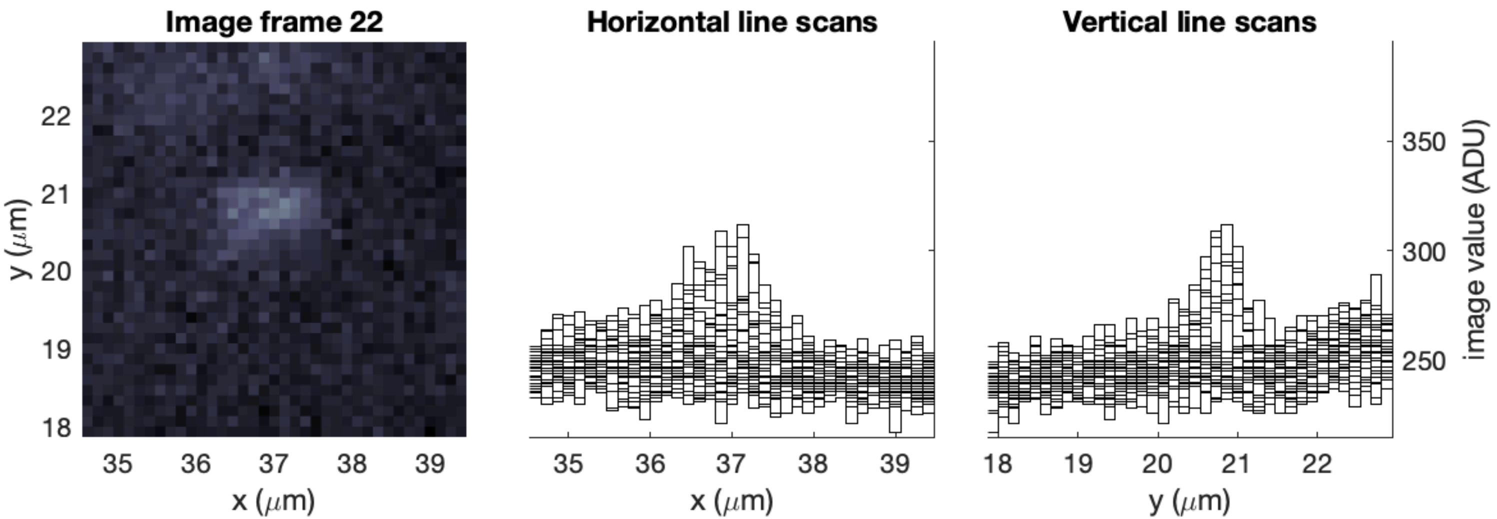
Image frame 22
Horizontal line scans
Vertical line scans
y (μm)
x (μm)
x (μm)
y (μm)
image value (ADU)
22
21
20
19
18
35
36
37
38
39
350
300
250
