# Supplementary material for: BNP-Track: a framework for superresolved tracking
Source: Nat Methods. 2024 Jul 22;21(9):1716–24. doi: 10.1038/s41592-024-02349-9 (PMC11399105; doi:10.1038/s41592-024-02349-9)
Supplement: Supplementary file 4 — Supplementary Data [file 41592_2024_2349_MOESM4_ESM.zip › Supplementary_Data/Supplementary_Data_3.pdf]

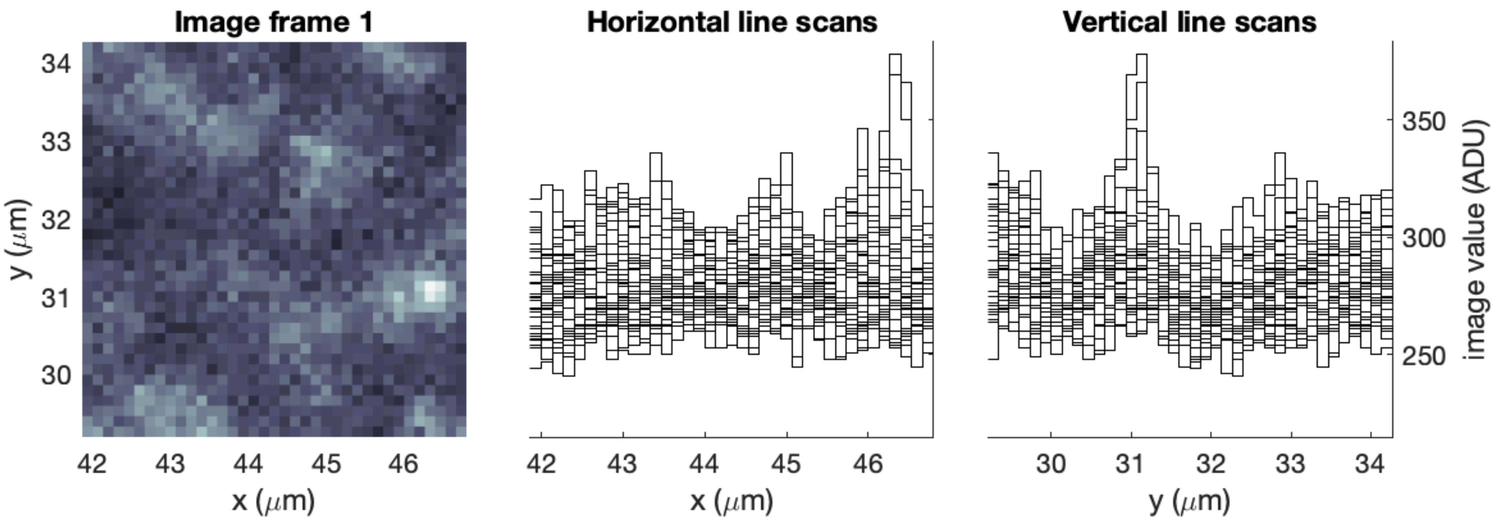

Image frame 1
Horizontal line scans
Vertical line scans
y (μm)
x (μm)
y (μm)
image value (ADU)
34
33
32
31
30
42
43
44
45
46
350
300
250

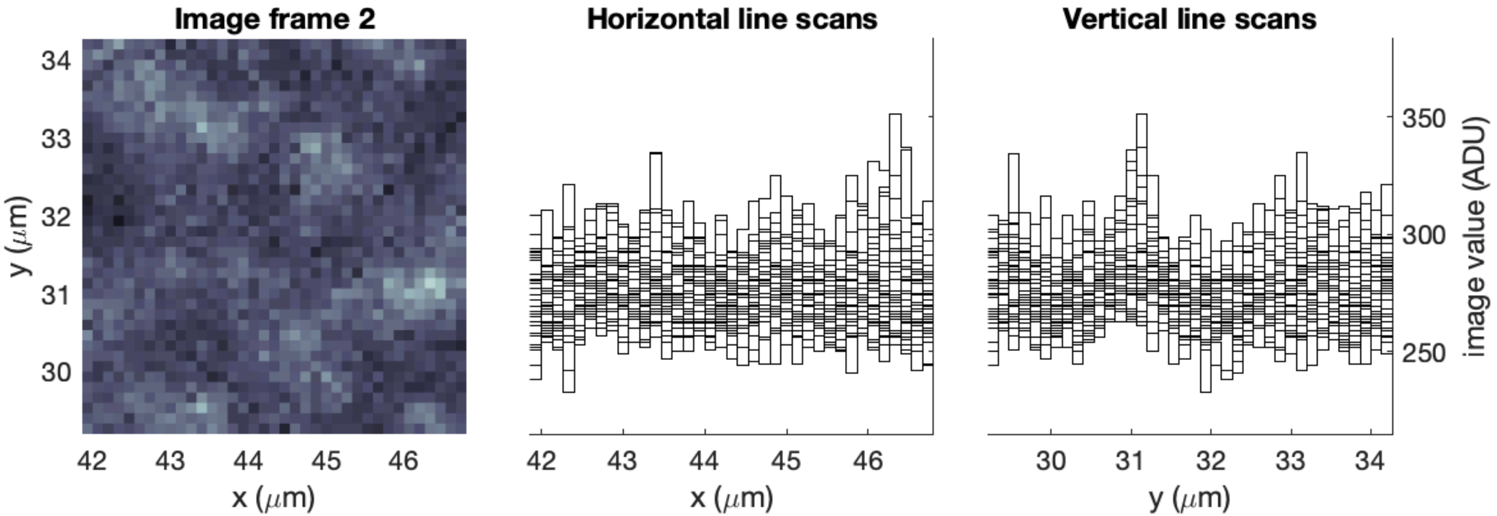

Image frame 2
Horizontal line scans
Vertical line scans
y (μm)
x (μm)
x (μm)
y (μm)
image value (ADU)
34
33
32
31
30
42
43
44
45
46
29
30
31
32
33
34
350
300
250

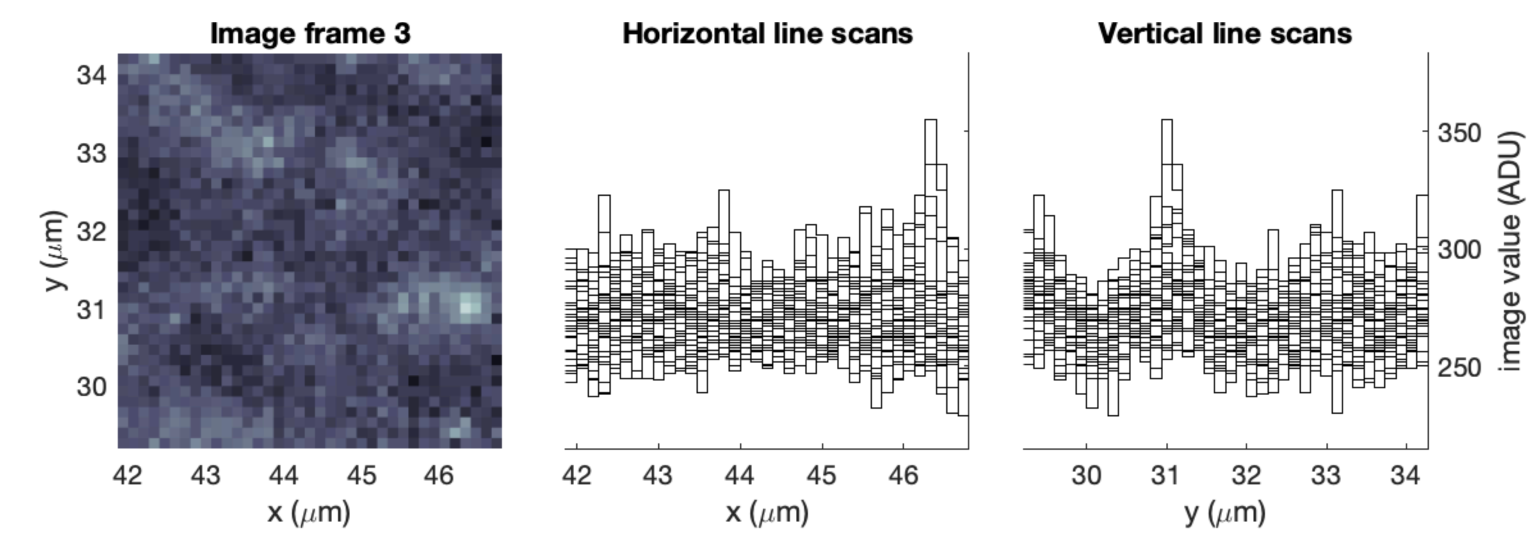

Image frame 3
Horizontal line scans
Vertical line scans
y (μm)
x (μm)
x (μm)
y (μm)
image value (ADU)
34
33
32
31
30
42
43
44
45
46
350
300
250

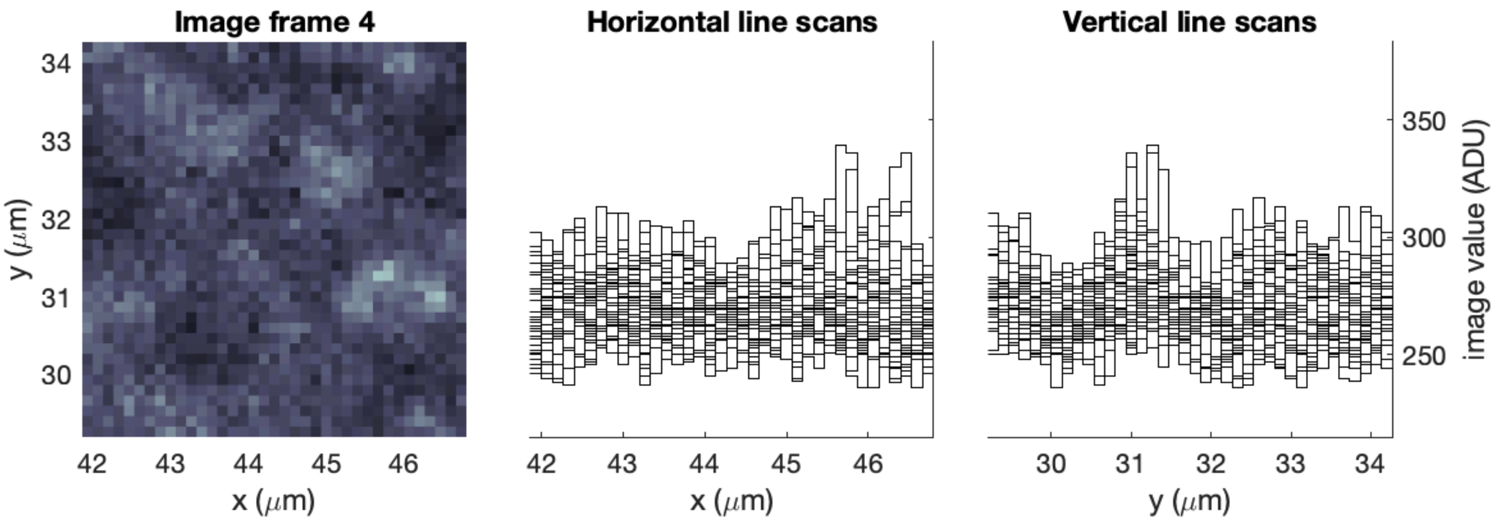
Image frame 4
Horizontal line scans
Vertical line scans
34
33
32
31
30
y (μm)
42
43
44
45
46
x (μm)
42
43
44
45
46
x (μm)
30
31
32
33
34
y (μm)
350
300
250
image value (ADU)

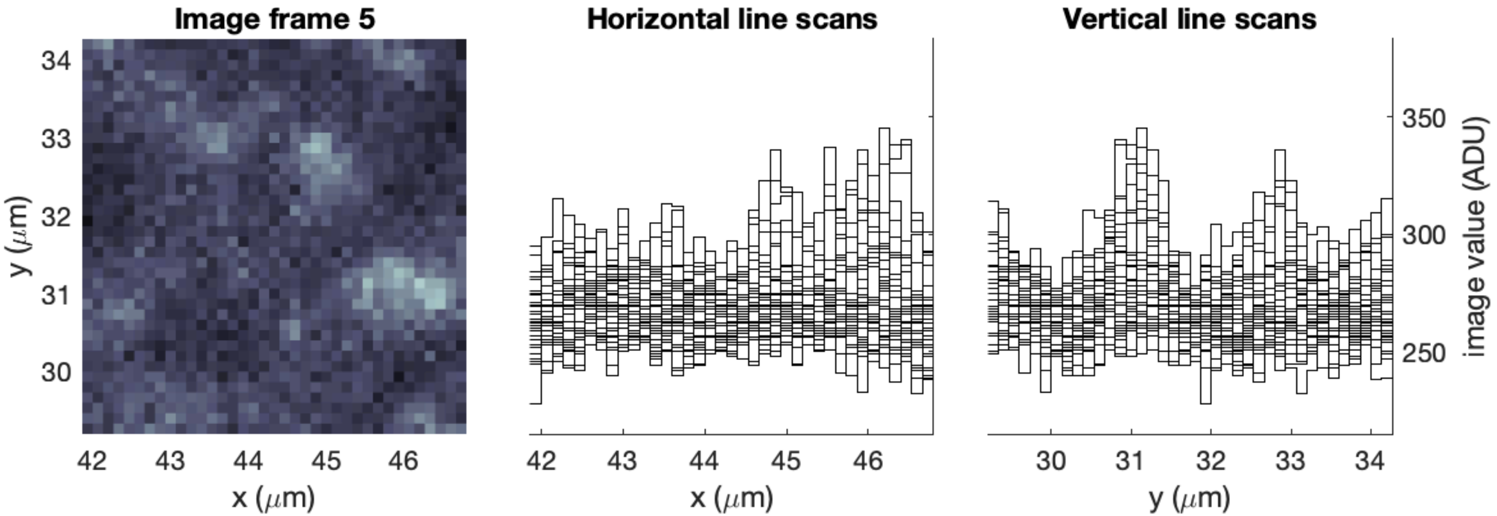

Image frame 5
Horizontal line scans
Vertical line scans
y (μm)
x (μm)
x (μm)
y (μm)
image value (ADU)
34
33
32
31
30
42
43
44
45
46
350
300
250

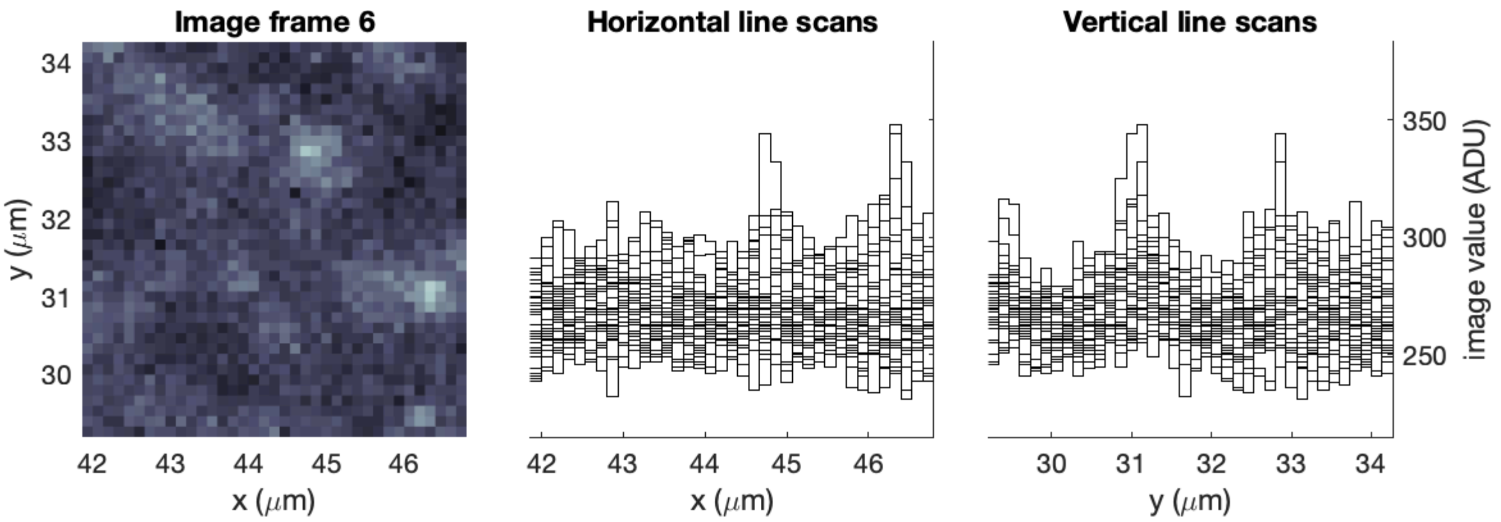

Image frame 6
Horizontal line scans
Vertical line scans
y (μm)
34
33
32
31
30
42
43
44
45
46
x (μm)
42
43
44
45
46
x (μm)
30
31
32
33
34
y (μm)
350
300
250
image value (ADU)

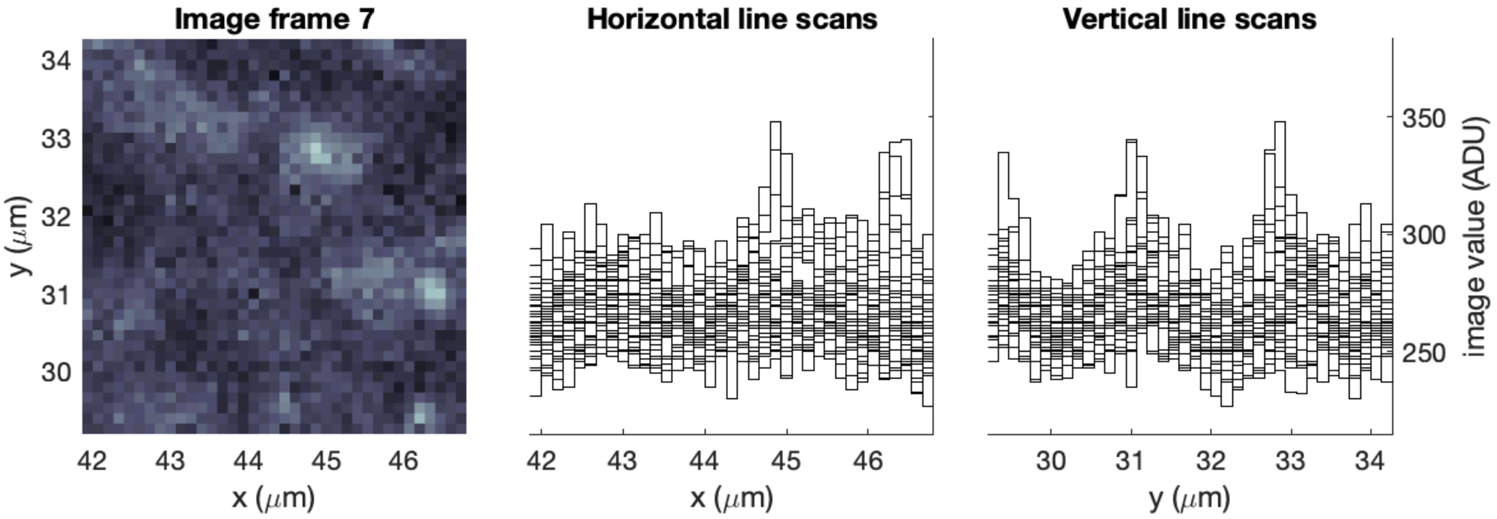
Image frame 7
Horizontal line scans
Vertical line scans
y (μm)
x (μm)
x (μm)
y (μm)
image value (ADU)
34
33
32
31
30
42
43
44
45
46
42
43
44
45
46
30
31
32
33
34
350
300
250

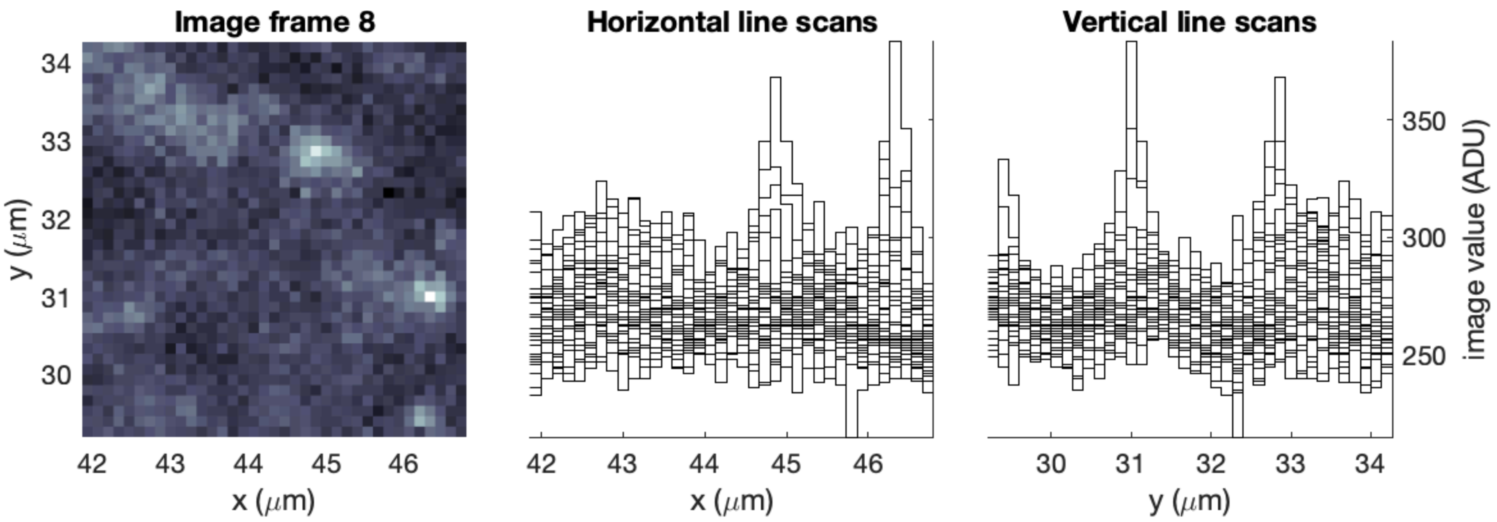
Image frame 8
Horizontal line scans
Vertical line scans
y (μm)
x (μm)
x (μm)
y (μm)
image value (ADU)
34
33
32
31
30
42
43
44
45
46
350
300
250

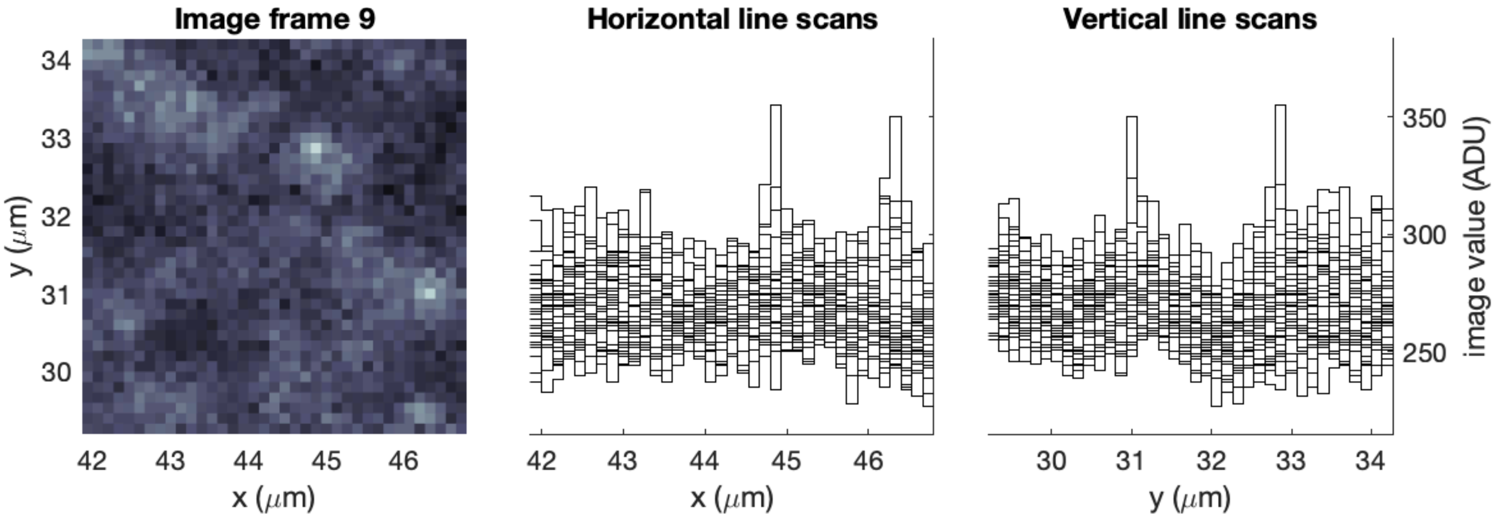

Image frame 9
Horizontal line scans
Vertical line scans
y (μm)
x (μm)
x (μm)
y (μm)
image value (ADU)
34
33
32
31
30
42
43
44
45
46
42
43
44
45
46
30
31
32
33
34
350
300
250

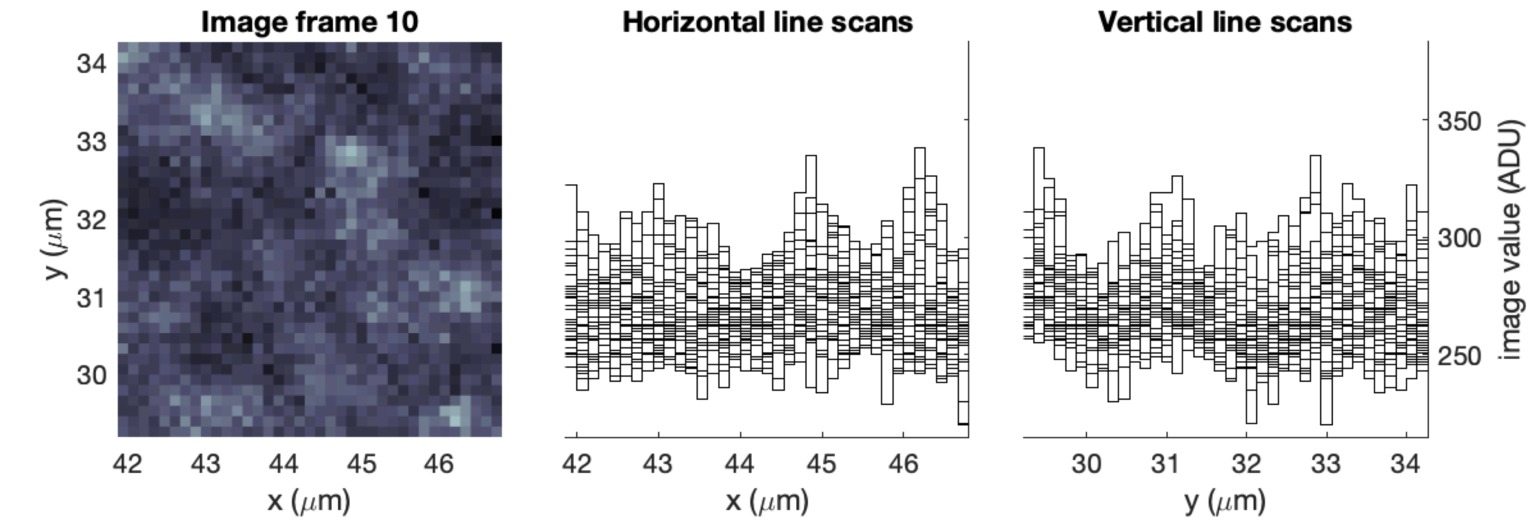

Image frame 10
Horizontal line scans
Vertical line scans
y (μm)
x (μm)
y (μm)
image value (ADU)

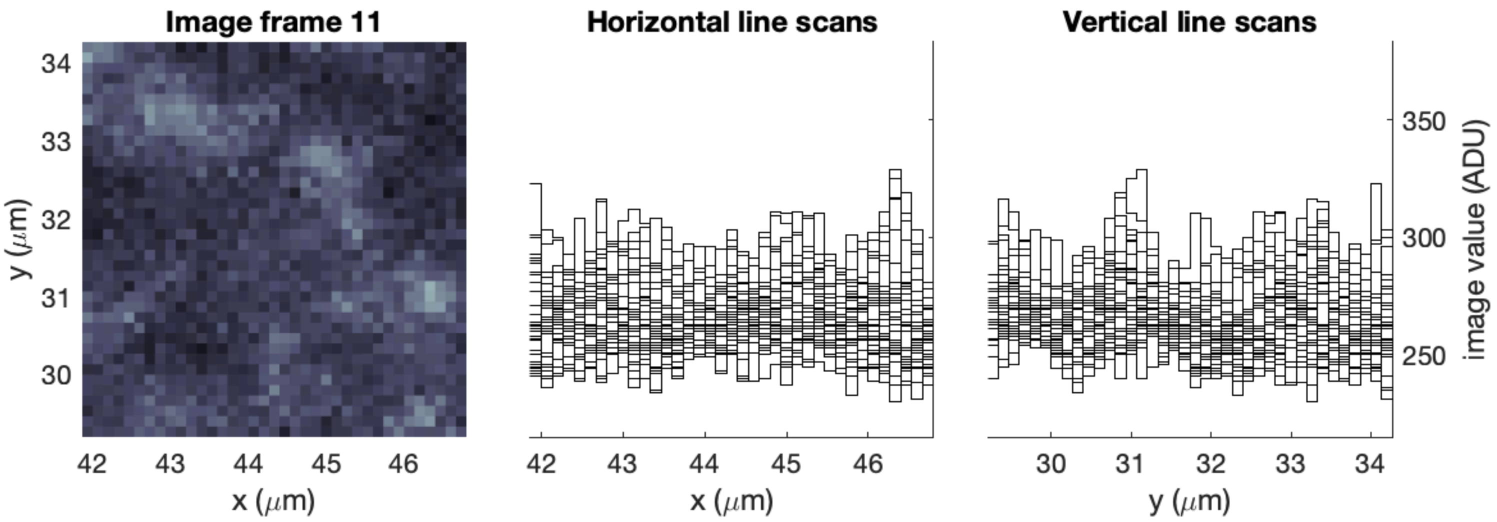

Image frame 11
Horizontal line scans
Vertical line scans
y (μm)
x (μm)
x (μm)
y (μm)
image value (ADU)
34
33
32
31
30
42
43
44
45
46
350
300
250

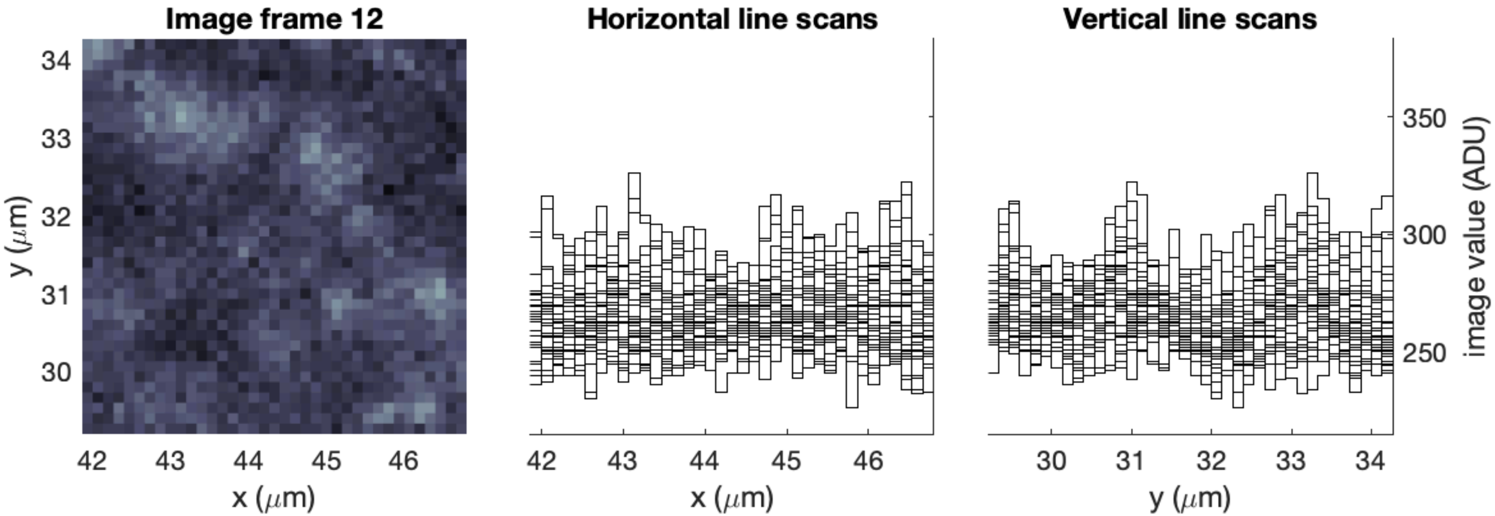

Image frame 12
Horizontal line scans
Vertical line scans
y (μm)
x (μm)
x (μm)
y (μm)
image value (ADU)
34
33
32
31
30
42
43
44
45
46
350
300
250

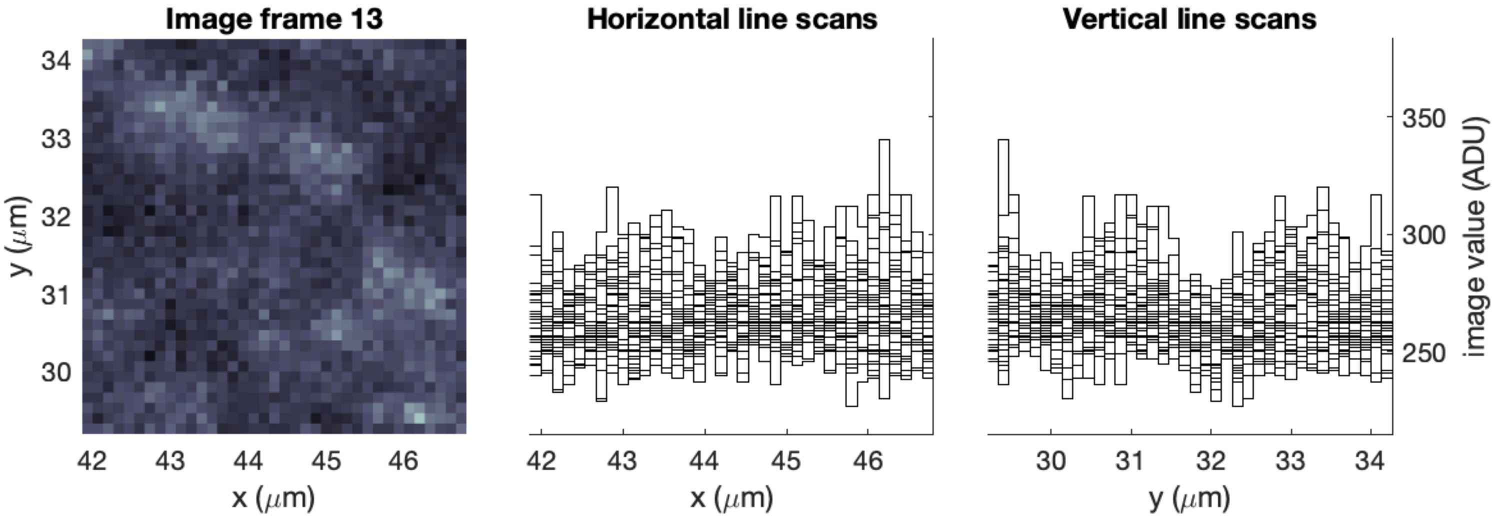
Image frame 13
Horizontal line scans
Vertical line scans
y (μm)
x (μm)
x (μm)
y (μm)
image value (ADU)
34
33
32
31
30
42
43
44
45
46
350
300
250

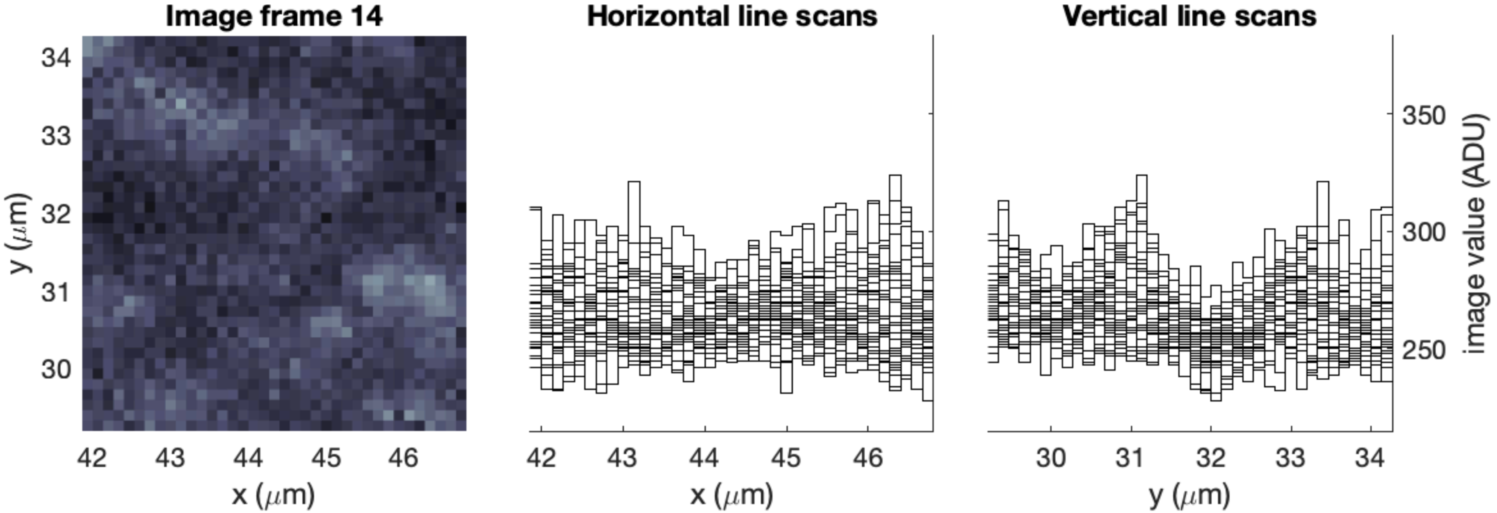
Image frame 14
Horizontal line scans
Vertical line scans
34
33
32
31
30
y (μm)
42
43
44
45
46
x (μm)
42
43
44
45
46
x (μm)
30
31
32
33
34
y (μm)
350
300
250
image value (ADU)

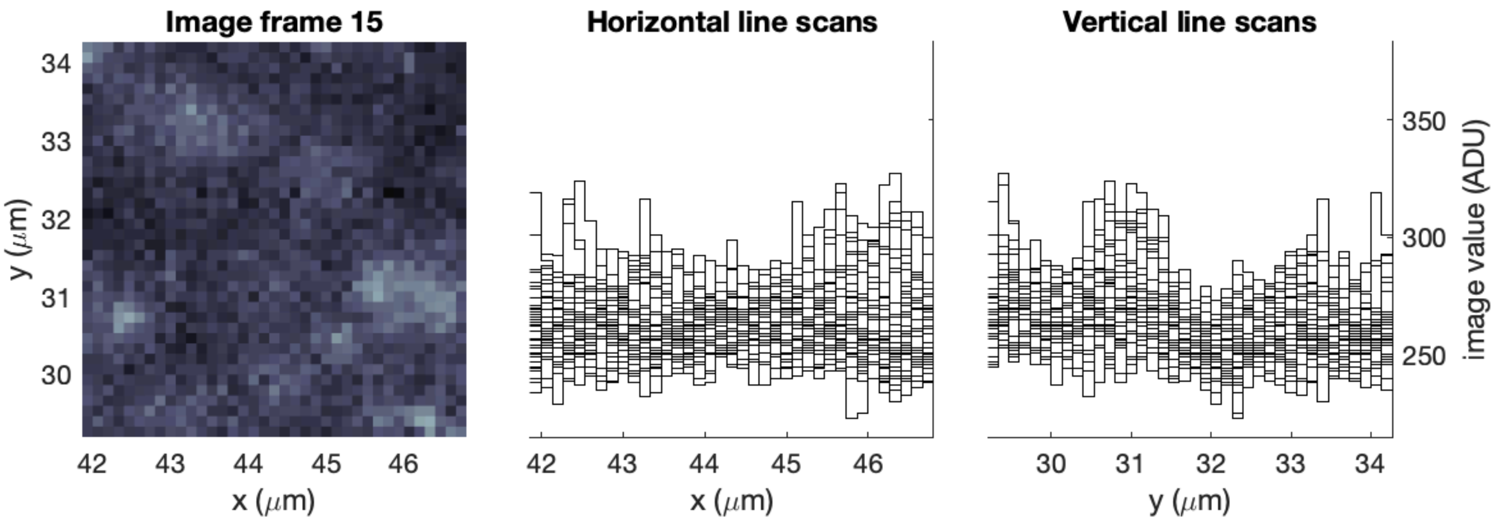

Image frame 15
Horizontal line scans
Vertical line scans
y (μm)
x (μm)
y (μm)
image value (ADU)
34
33
32
31
30
42
43
44
45
46
350
300
250

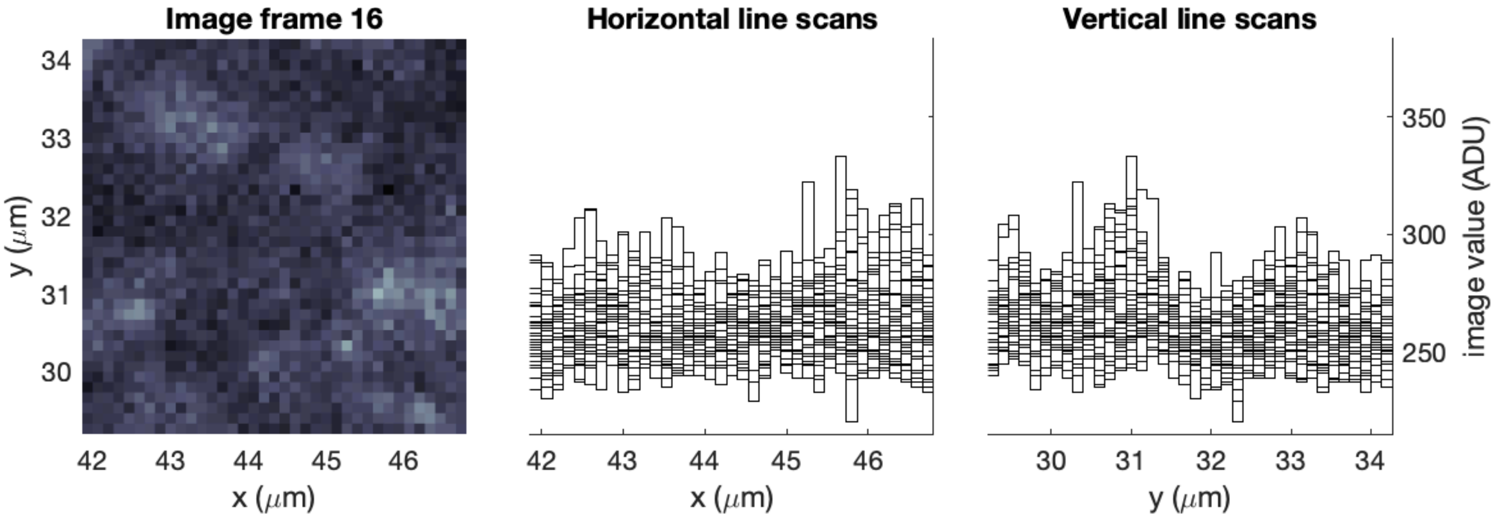
Image frame 16
Horizontal line scans
Vertical line scans
y (μm)
x (μm)
x (μm)
y (μm)
image value (ADU)
34
33
32
31
30
42
43
44
45
46
350
300
250

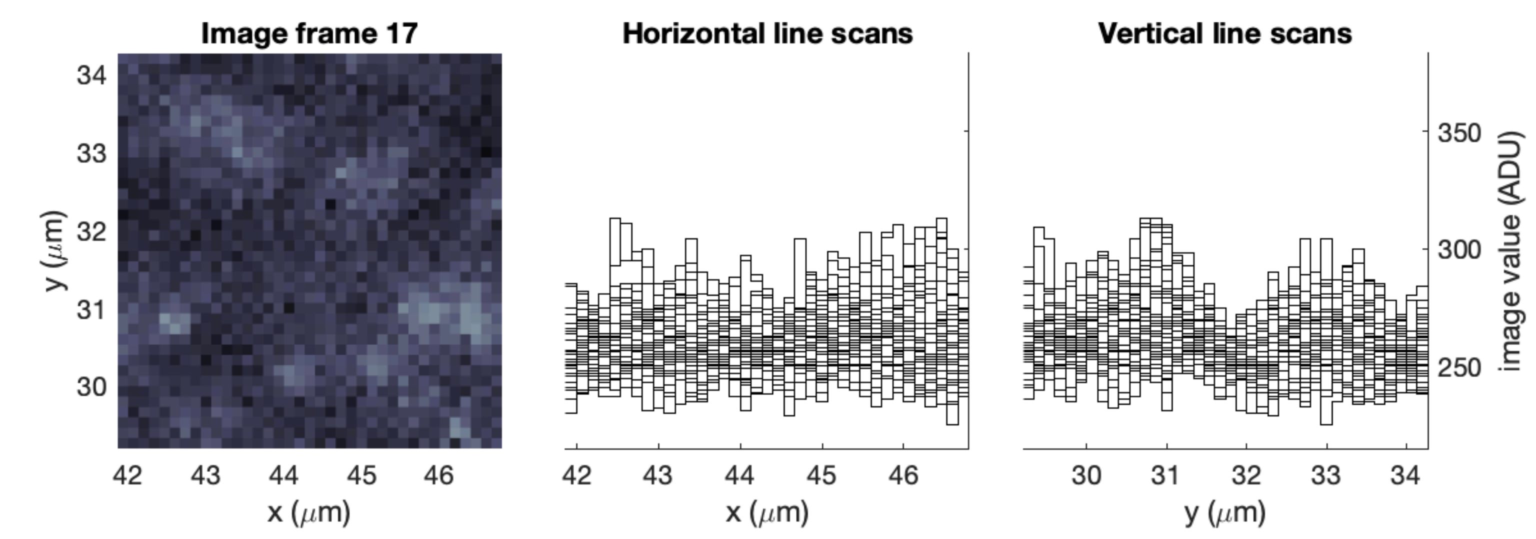

Image frame 17
Horizontal line scans
Vertical line scans
34
33
32
31
30
y (μm)
42
43
44
45
46
x (μm)
42
43
44
45
46
x (μm)
30
31
32
33
34
y (μm)
350
300
250
image value (ADU)

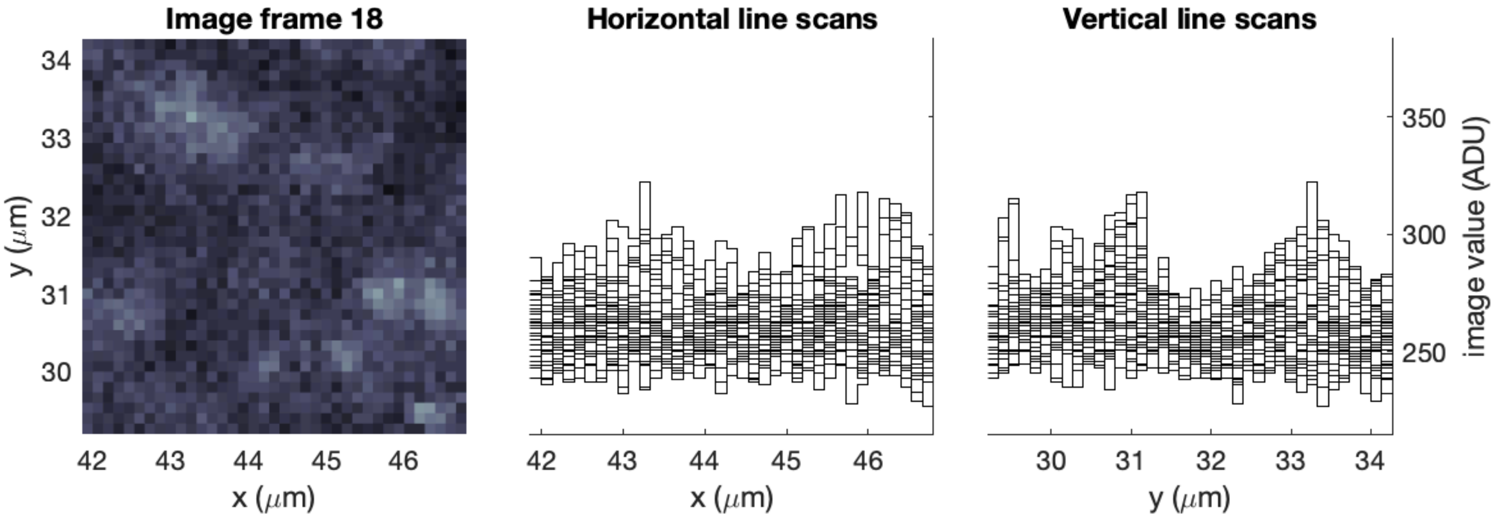
Image frame 18
Horizontal line scans
Vertical line scans
y (μm)
x (μm)
image value (ADU)
34
33
32
31
30
42
43
44
45
46
350
300
250

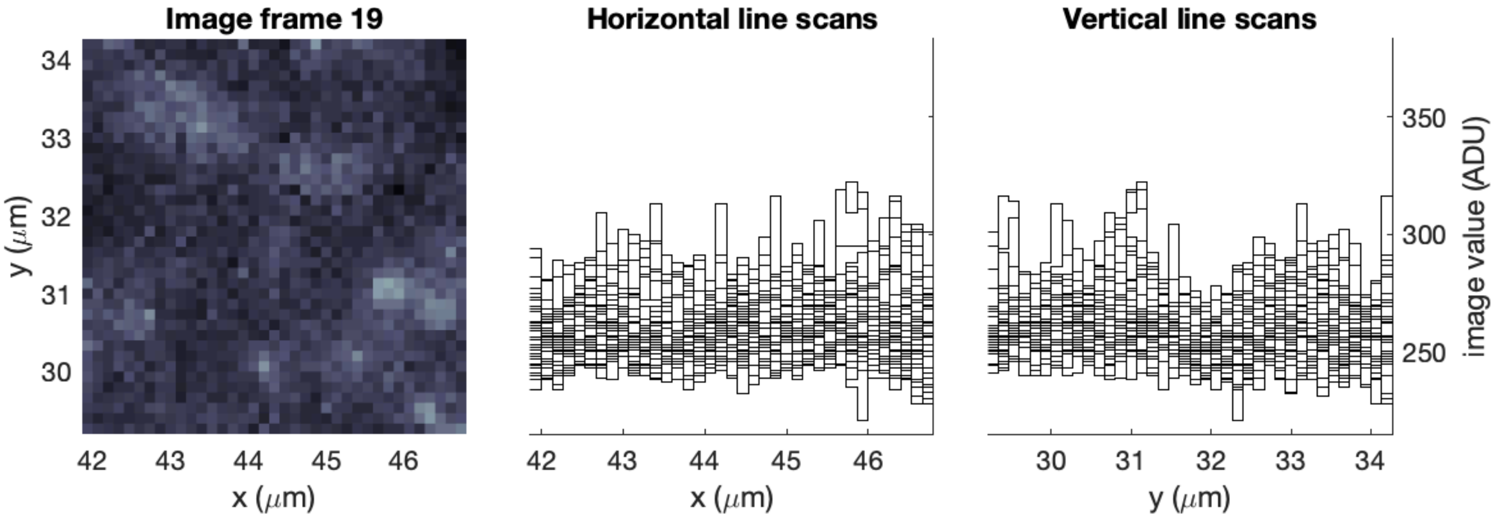

Image frame 19
Horizontal line scans
Vertical line scans
y (μm)
x (μm)
x (μm)
y (μm)
image value (ADU)
34
33
32
31
30
42
43
44
45
46
30
31
32
33
34
350
300
250

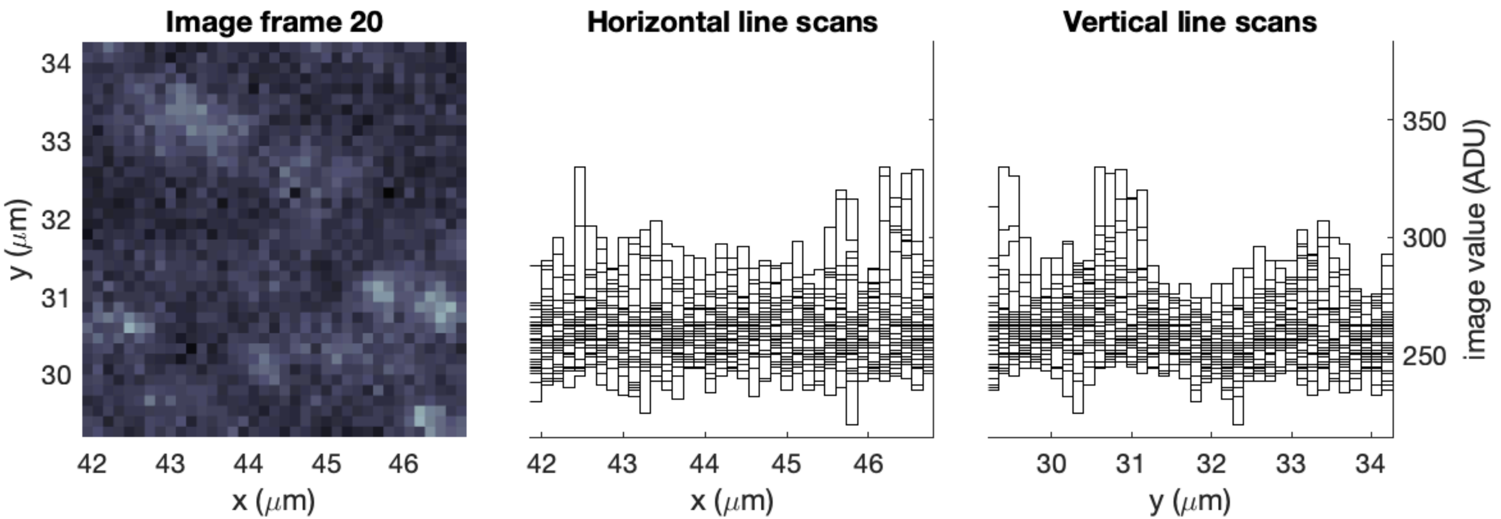

Image frame 20
y (μm)
34
33
32
31
30
42
43
44
45
46
x (μm)
Horizontal line scans
42
43
44
45
46
x (μm)
Vertical line scans
30
31
32
33
34
y (μm)
350
300
250
image value (ADU)

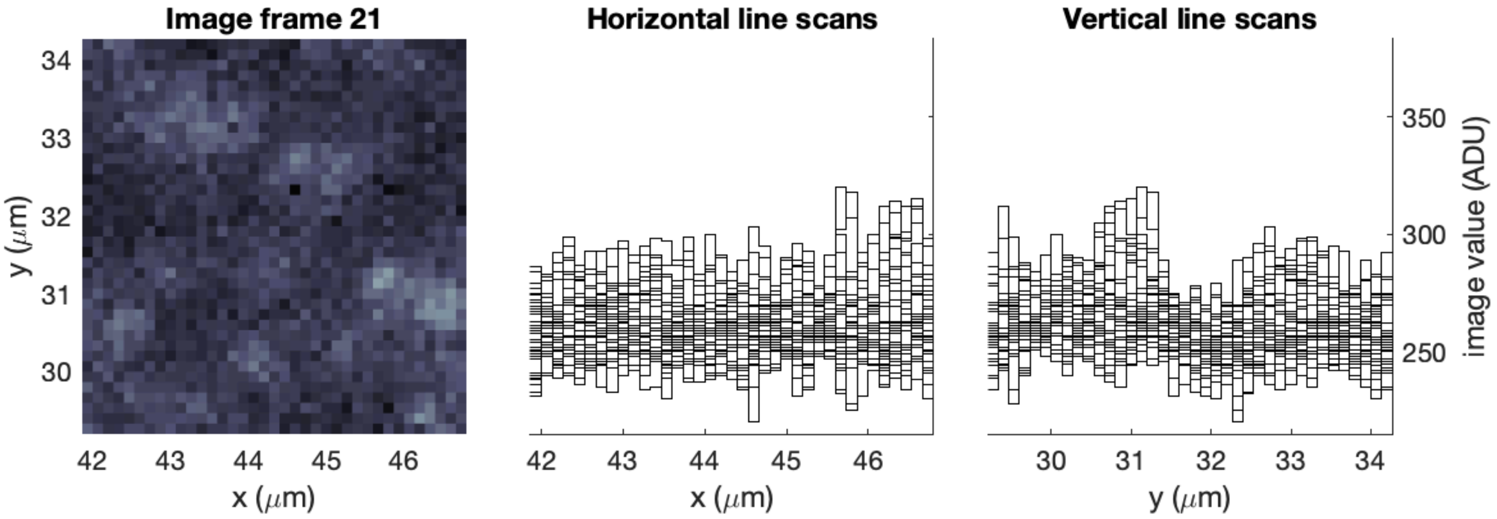

Image frame 21
Horizontal line scans
Vertical line scans
y (μm)
34
33
32
31
30
42
43
44
45
46
x (μm)
42
43
44
45
46
x (μm)
30
31
32
33
34
y (μm)
350
300
250
image value (ADU)

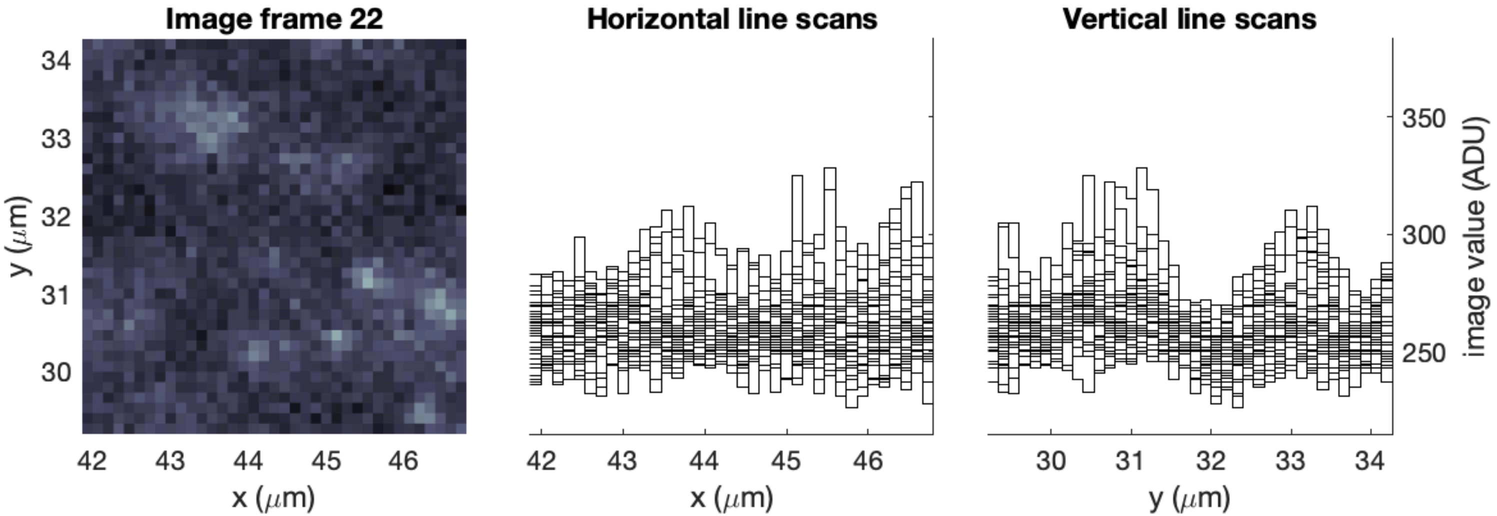

Image frame 22
Horizontal line scans
Vertical line scans
y (μm)
x (μm)
x (μm)
y (μm)
image value (ADU)
34
33
32
31
30
42
43
44
45
46
350
300
250
