# Supplementary material for: BNP-Track: a framework for superresolved tracking
Source: Nat Methods. 2024 Jul 22;21(9):1716–24. doi: 10.1038/s41592-024-02349-9 (PMC11399105; doi:10.1038/s41592-024-02349-9)
Supplement: Supplementary file 4 — Supplementary Data [file 41592_2024_2349_MOESM4_ESM.zip › Supplementary_Data/Supplementary_Data_18.pdf]

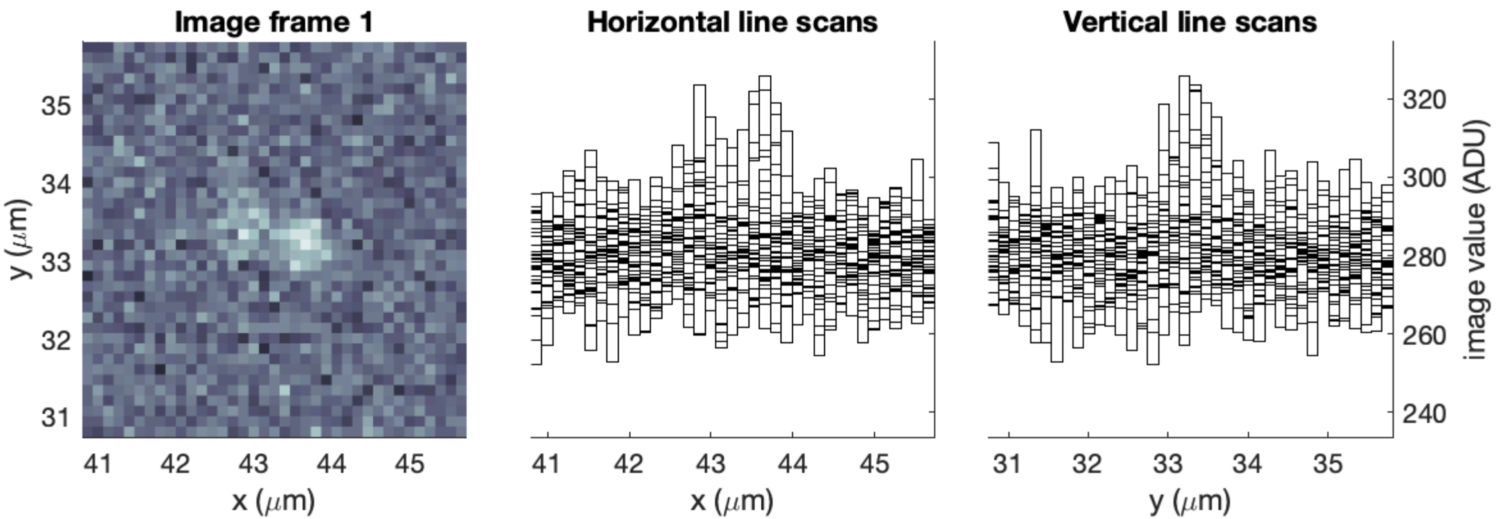

Image frame 1
Horizontal line scans
Vertical line scans
y (μm)
x (μm)
x (μm)
y (μm)
image value (ADU)
35
34
33
32
31
41
42
43
44
45
41
42
43
44
45
31
32
33
34
35
320
300
280
260
240

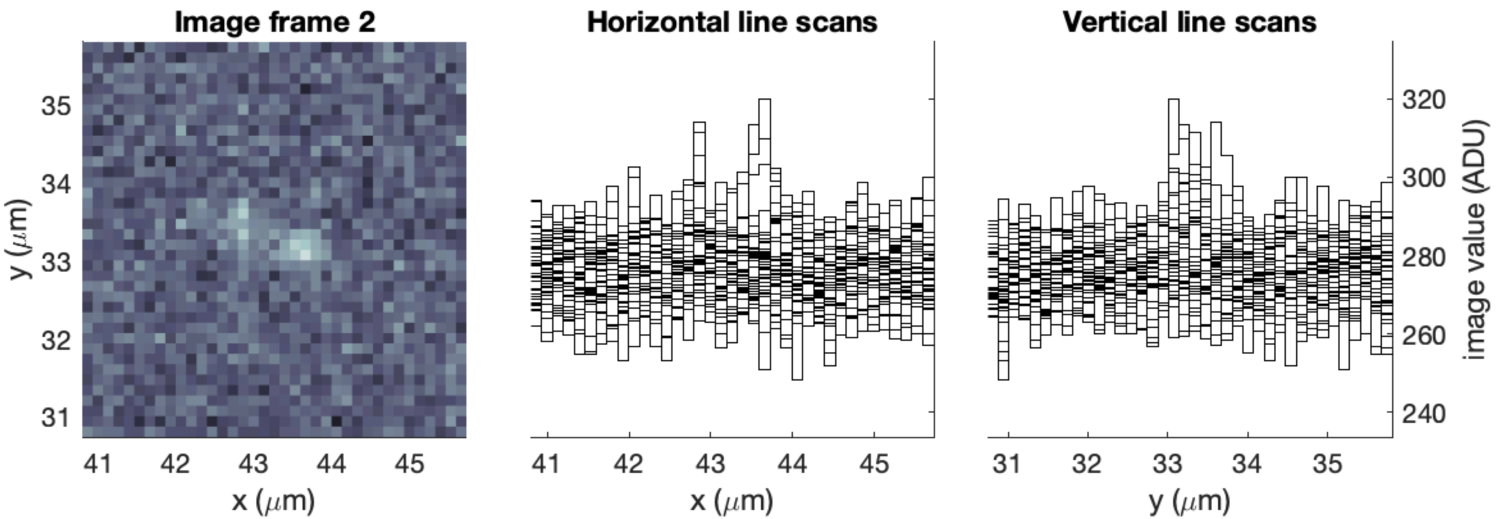

Image frame 2
Horizontal line scans
Vertical line scans
y (μm)
x (μm)
x (μm)
y (μm)
image value (ADU)
35
34
33
32
31
41
42
43
44
45
31
32
33
34
35
320
300
280
260
240

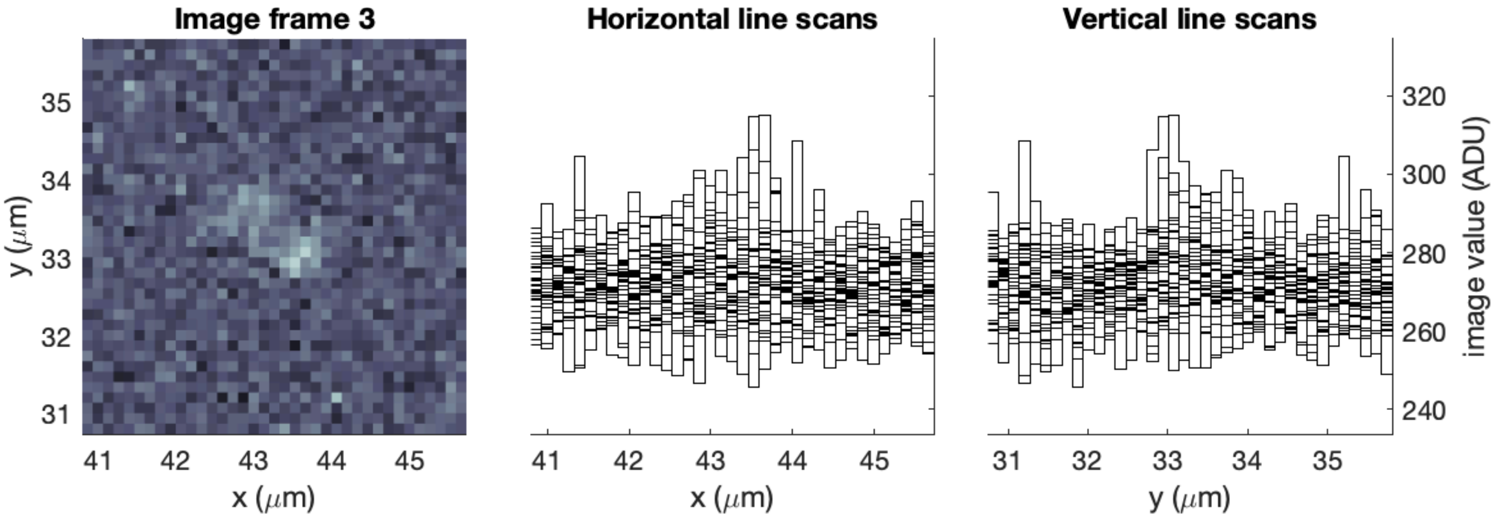

Image frame 3
Horizontal line scans
Vertical line scans
y (μm)
x (μm)
x (μm)
y (μm)
image value (ADU)
35
34
33
32
31
41
42
43
44
45
31
32
33
34
35
320
300
280
260
240

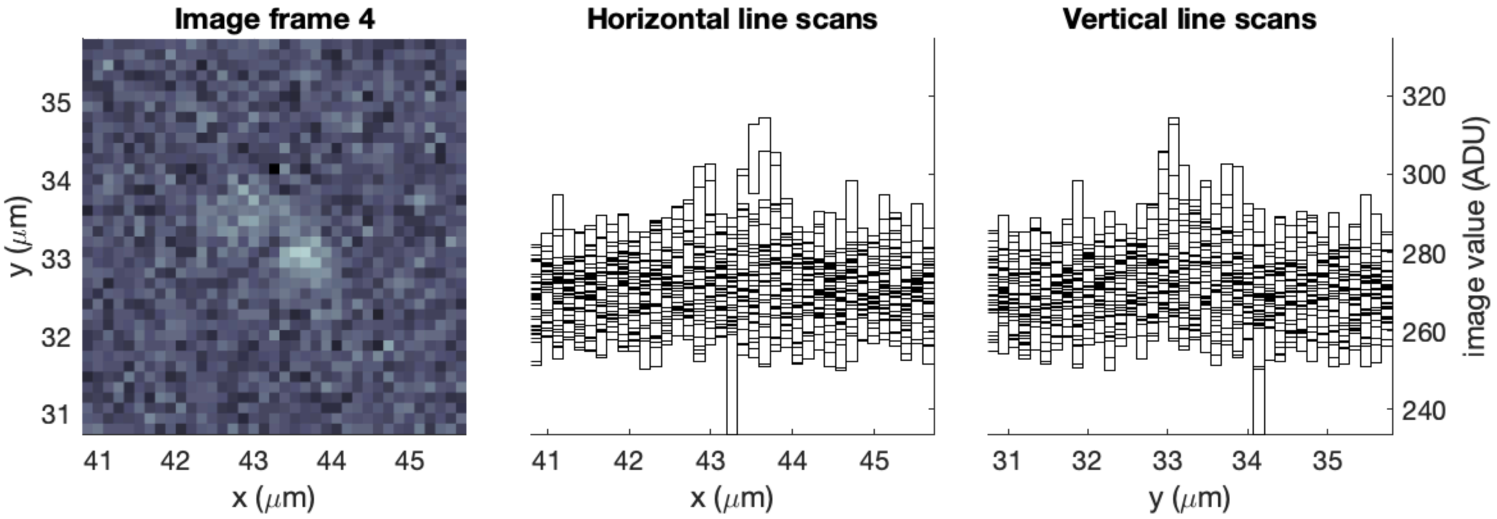

Image frame 4
Horizontal line scans
Vertical line scans
y (μm)
x (μm)
x (μm)
y (μm)
image value (ADU)
35
34
33
32
31
41
42
43
44
45
41
42
43
44
45
31
32
33
34
35
320
300
280
260
240

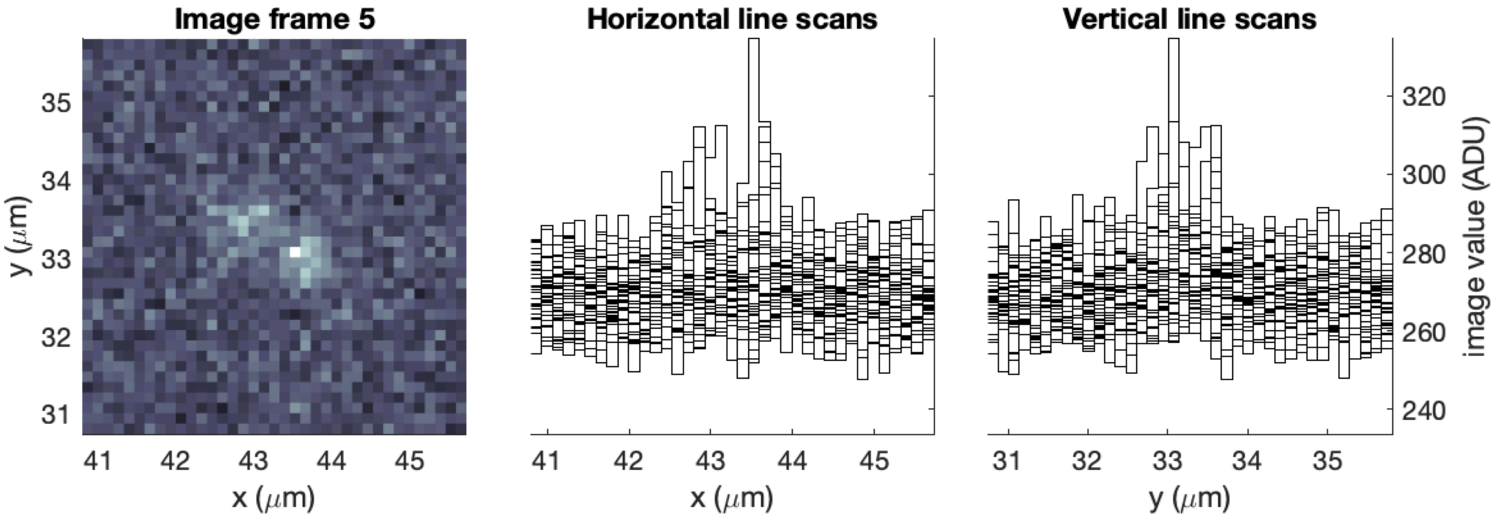

Image frame 5
Horizontal line scans
Vertical line scans
y (μm)
35
34
33
32
31
41
42
43
44
45
x (μm)
x (μm)
y (μm)
320
300
280
260
240
image value (ADU)

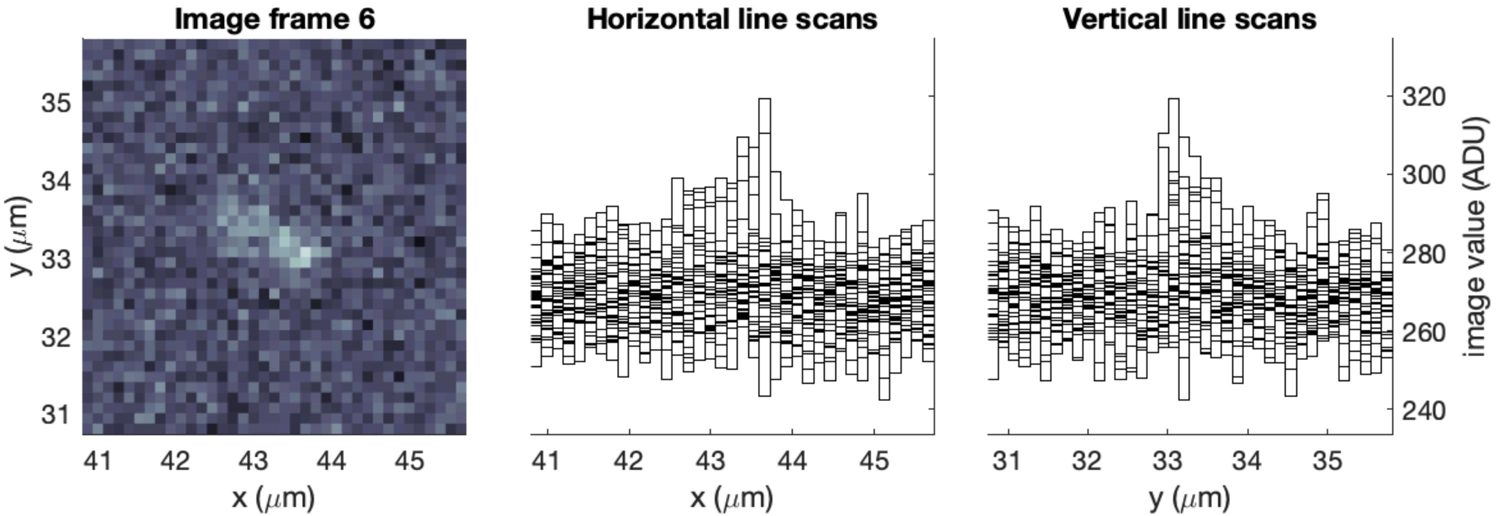
Image frame 6
Horizontal line scans
Vertical line scans
y (μm)
x (μm)
x (μm)
y (μm)
image value (ADU)
35
34
33
32
31
41
42
43
44
45
320
300
280
260
240

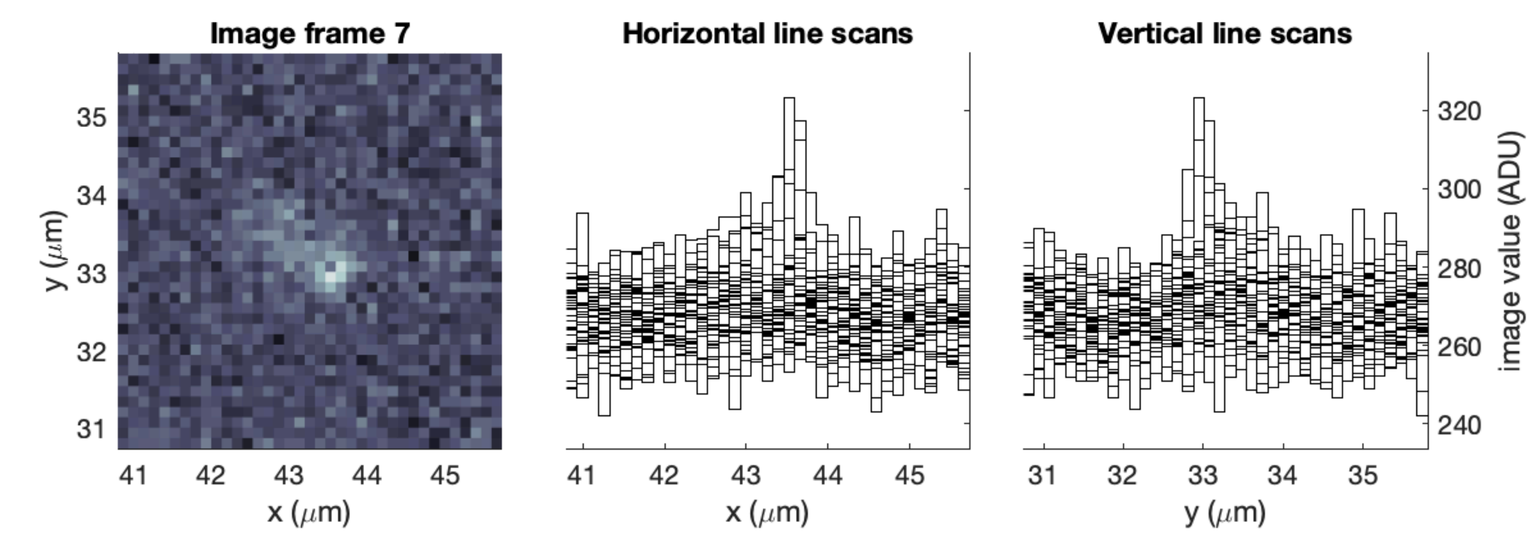

Image frame 7
Horizontal line scans
Vertical line scans
y (μm)
x (μm)
x (μm)
y (μm)
image value (ADU)
35
34
33
32
31
41
42
43
44
45
41
42
43
44
45
31
32
33
34
35
320
300
280
260
240

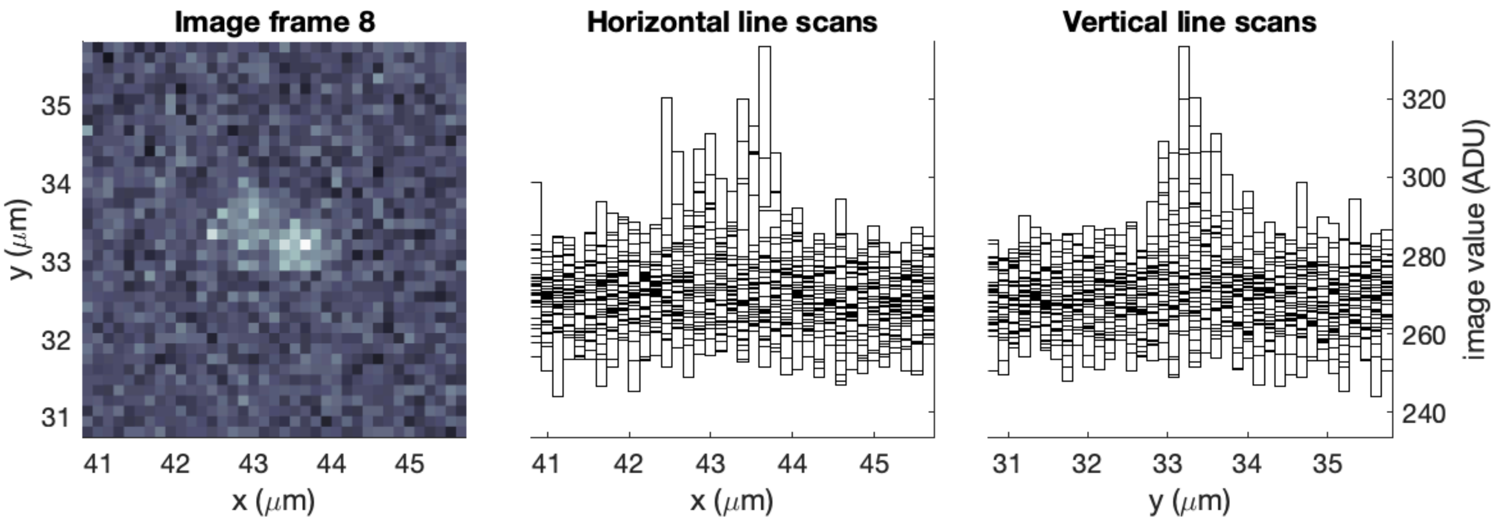

Image frame 8
Horizontal line scans
Vertical line scans
y (μm)
x (μm)
x (μm)
y (μm)
image value (ADU)
35
34
33
32
31
41
42
43
44
45
31
32
33
34
35
320
300
280
260
240

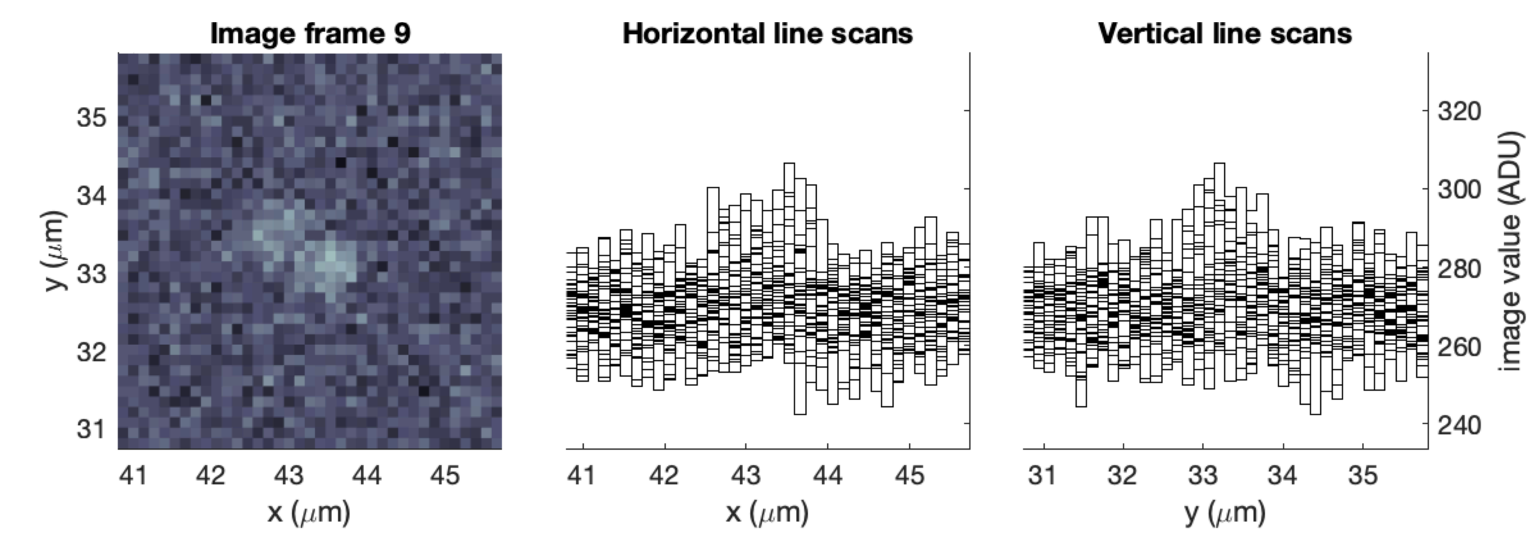

Image frame 9
Horizontal line scans
Vertical line scans
y (μm)
x (μm)
x (μm)
y (μm)
image value (ADU)
35
34
33
32
31
41
42
43
44
45
41
42
43
44
45
31
32
33
34
35
320
300
280
260
240

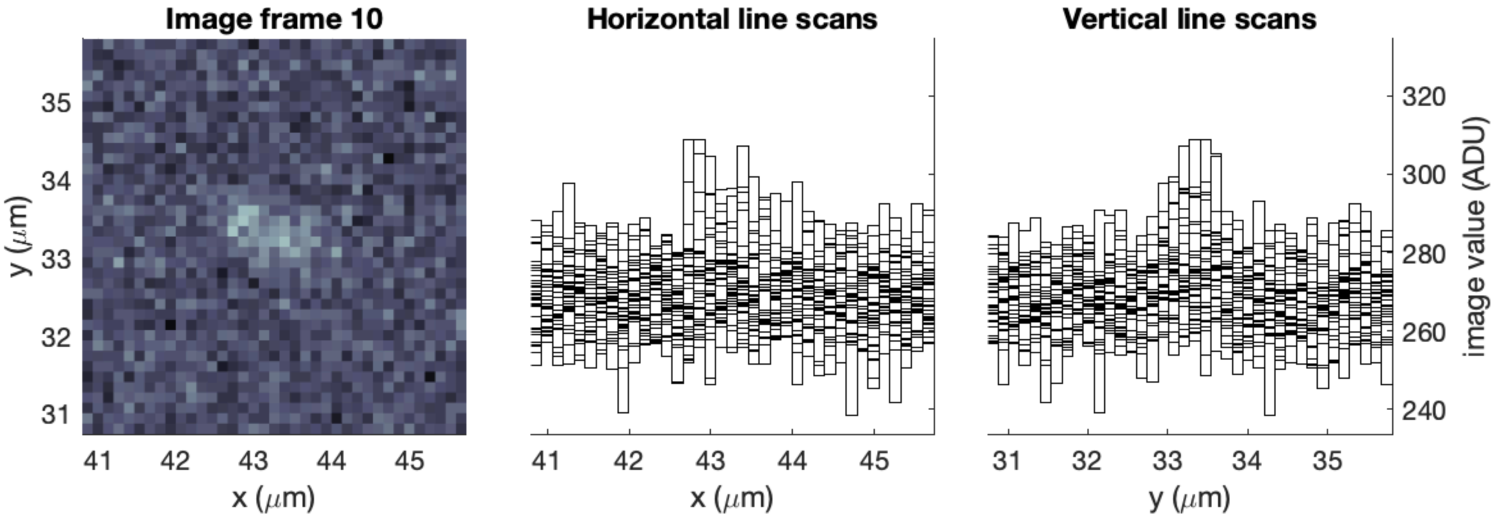

Image frame 10
Horizontal line scans
Vertical line scans
y (μm)
35
34
33
32
31
41
42
43
44
45
x (μm)
41
42
43
44
45
x (μm)
31
32
33
34
35
y (μm)
320
300
280
260
240
image value (ADU)

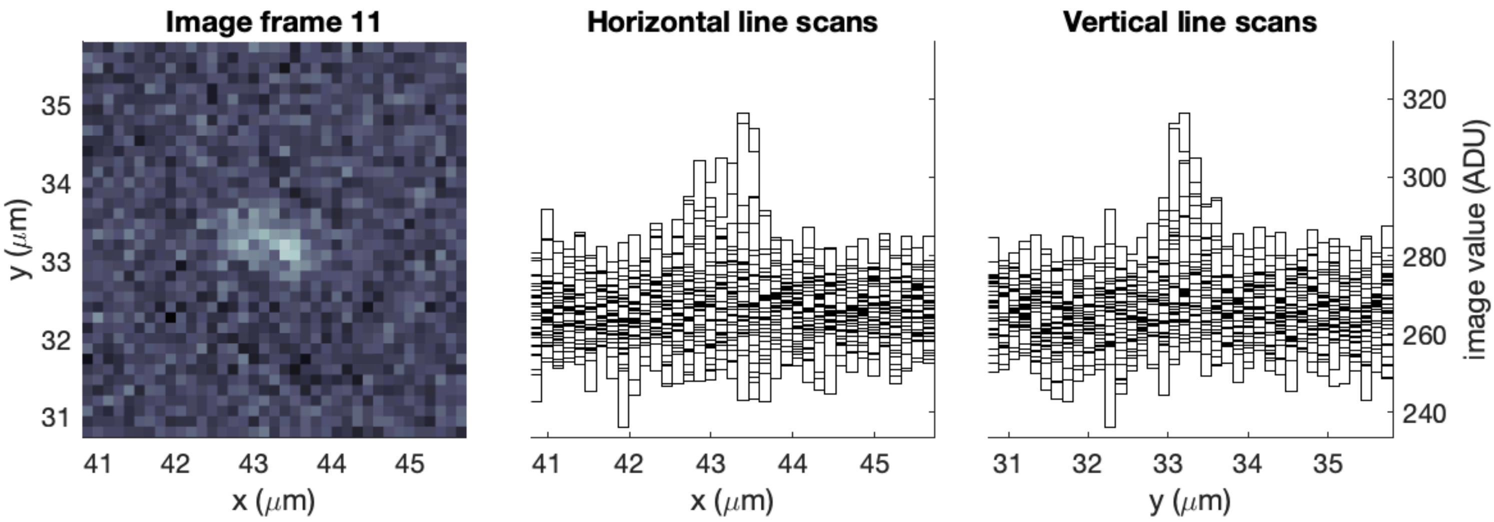

Image frame 11
Horizontal line scans
Vertical line scans
y (μm)
35
34
33
32
31
41
42
43
44
45
x (μm)
x (μm)
y (μm)
320
300
280
260
240
image value (ADU)

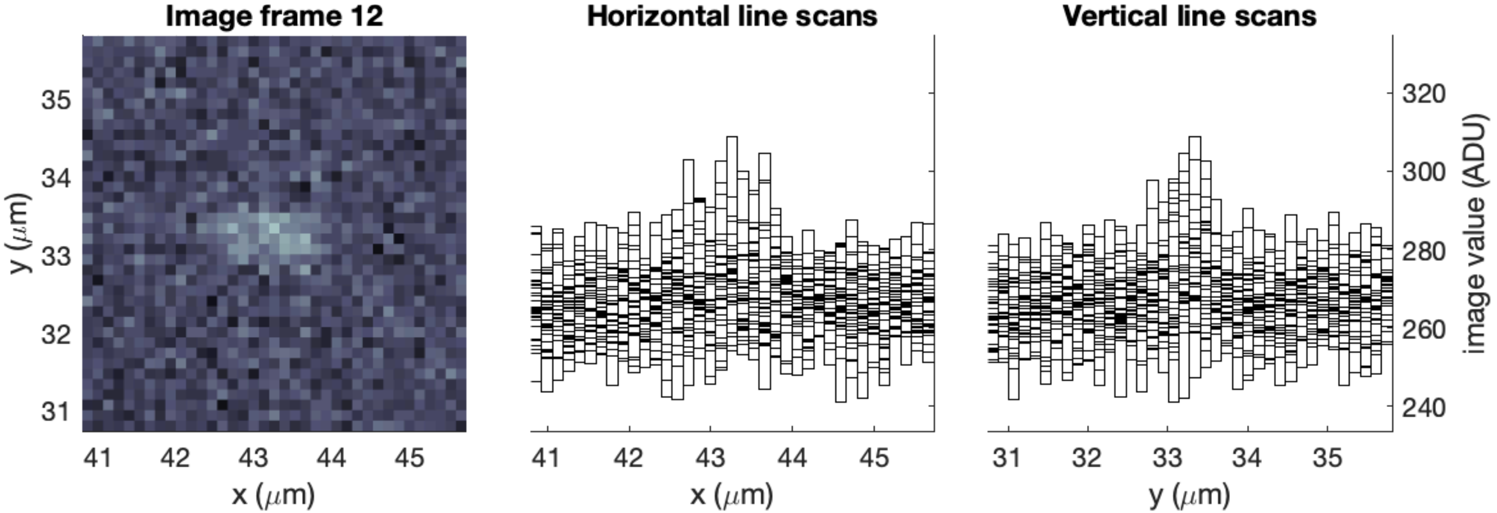

Image frame 12
Horizontal line scans
Vertical line scans
35
34
33
32
31
y (μm)
41
42
43
44
45
x (μm)
41
42
43
44
45
x (μm)
31
32
33
34
35
y (μm)
320
300
280
260
240
image value (ADU)

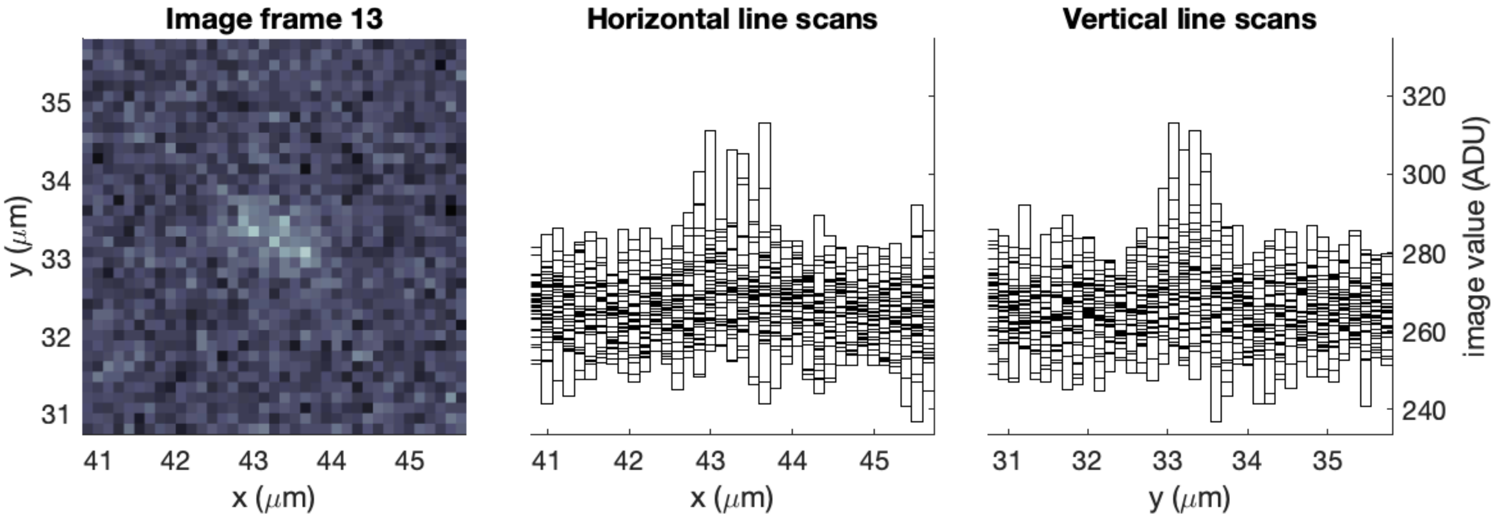

Image frame 13
Horizontal line scans
Vertical line scans
35
34
33
32
31
y (μm)
41
42
43
44
45
x (μm)
41
42
43
44
45
x (μm)
31
32
33
34
35
y (μm)
320
300
280
260
240
image value (ADU)

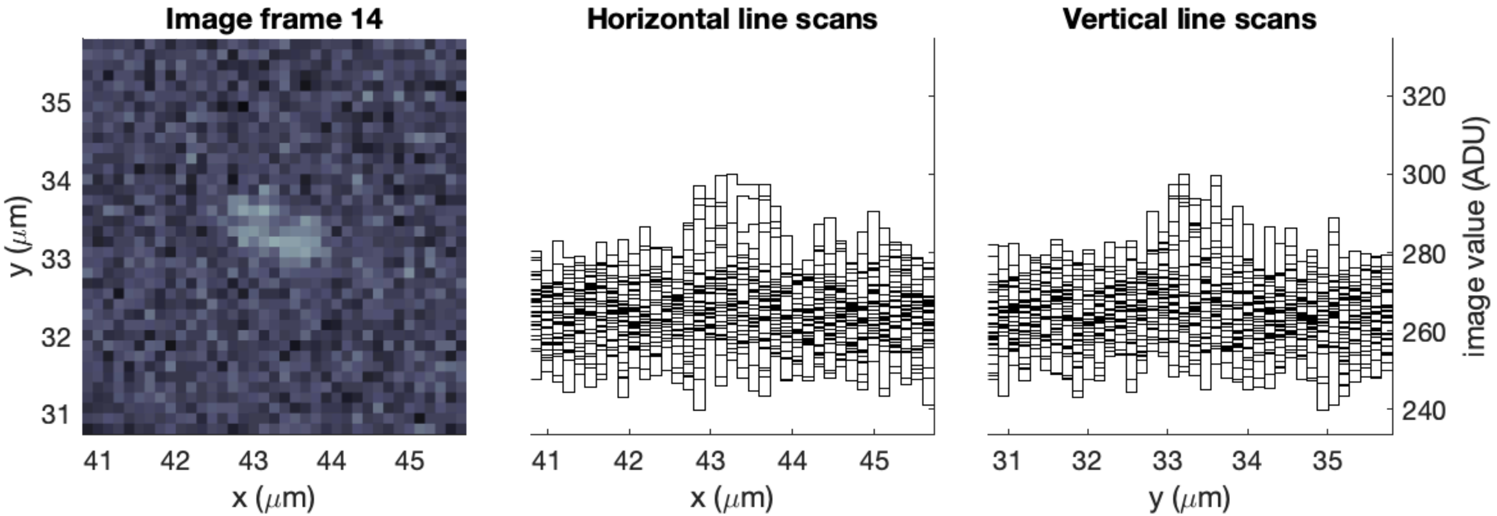

Image frame 14
Horizontal line scans
Vertical line scans
y (μm)
35
34
33
32
31
41
42
43
44
45
x (μm)
x (μm)
y (μm)
320
300
280
260
240
image value (ADU)

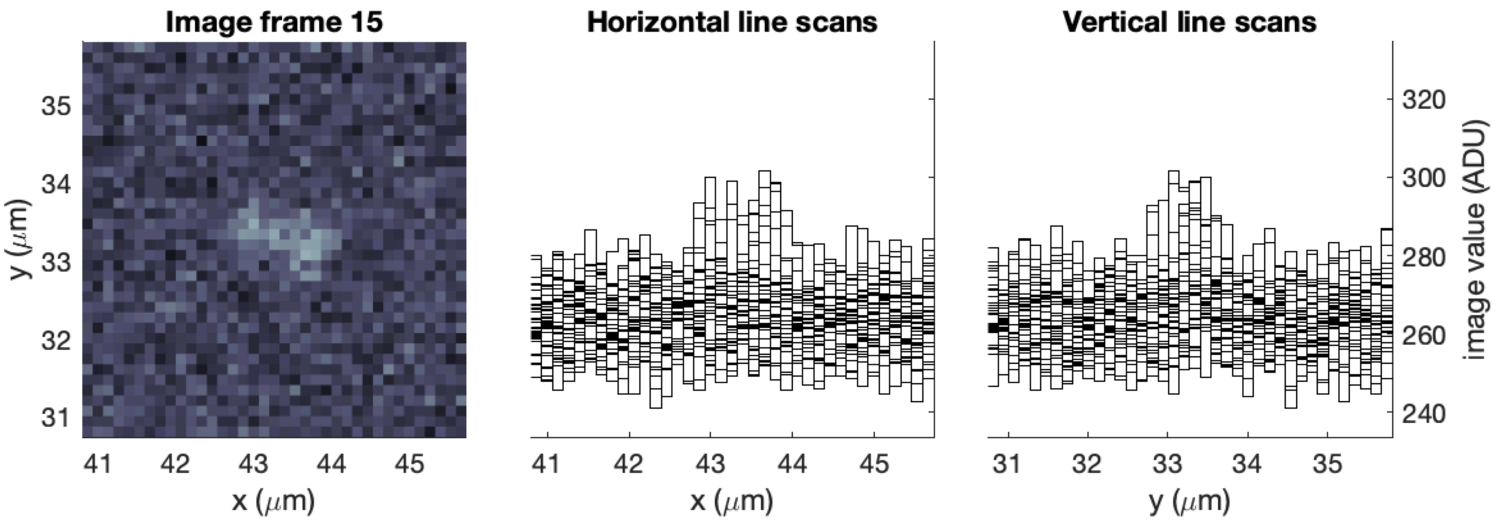

Image frame 15
Horizontal line scans
Vertical line scans
y (μm)
x (μm)
x (μm)
y (μm)
image value (ADU)
31
32
33
34
35
41
42
43
44
45
240
260
280
300
320

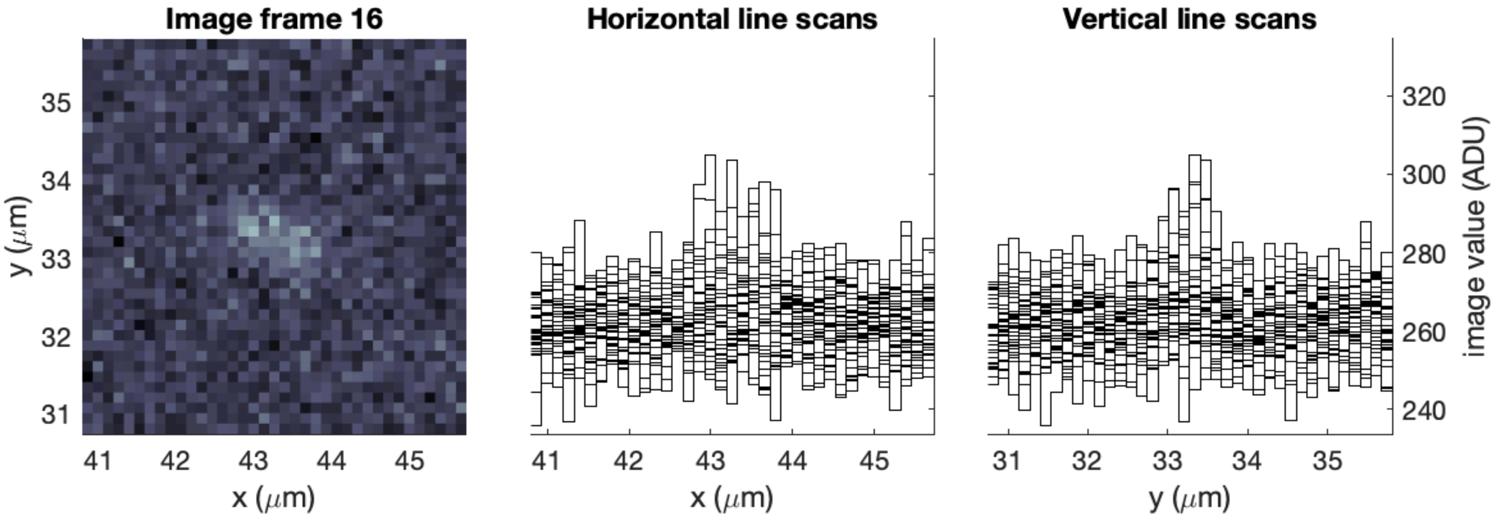

Image frame 16
Horizontal line scans
Vertical line scans
y (μm)
x (μm)
x (μm)
y (μm)
image value (ADU)
35
34
33
32
31
41
42
43
44
45
31
32
33
34
35
320
300
280
260
240

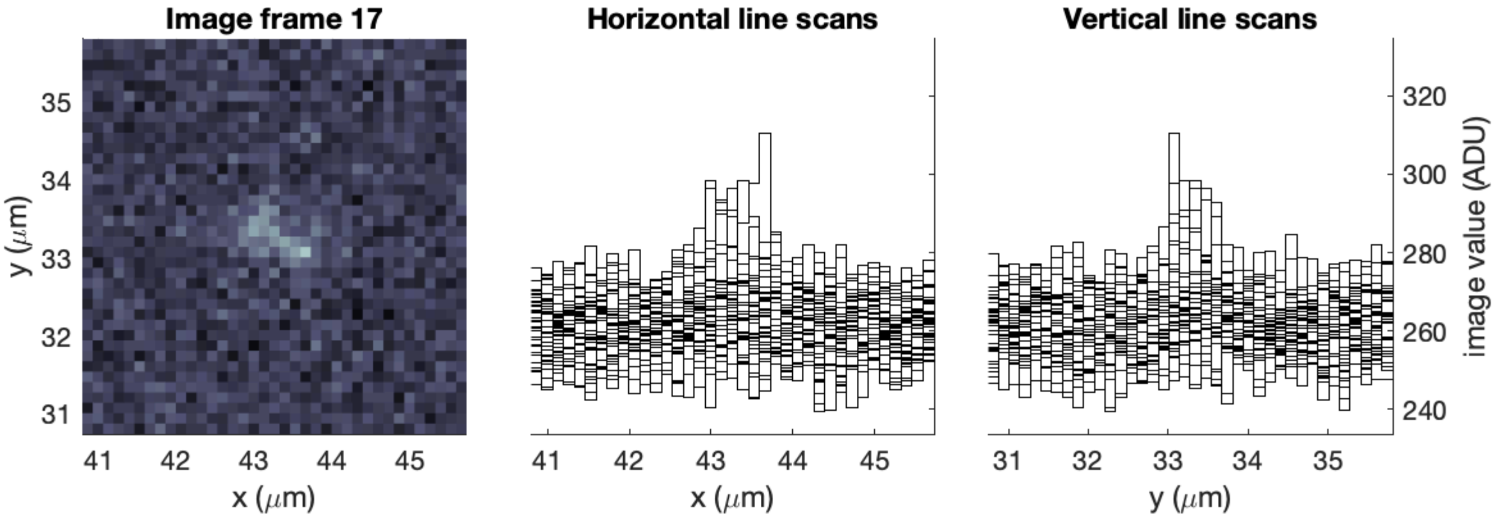
Image frame 17
Horizontal line scans
Vertical line scans
y (μm)
35
34
33
32
31
41
42
43
44
45
x (μm)
41
42
43
44
45
x (μm)
31
32
33
34
35
y (μm)
320
300
280
260
240
image value (ADU)

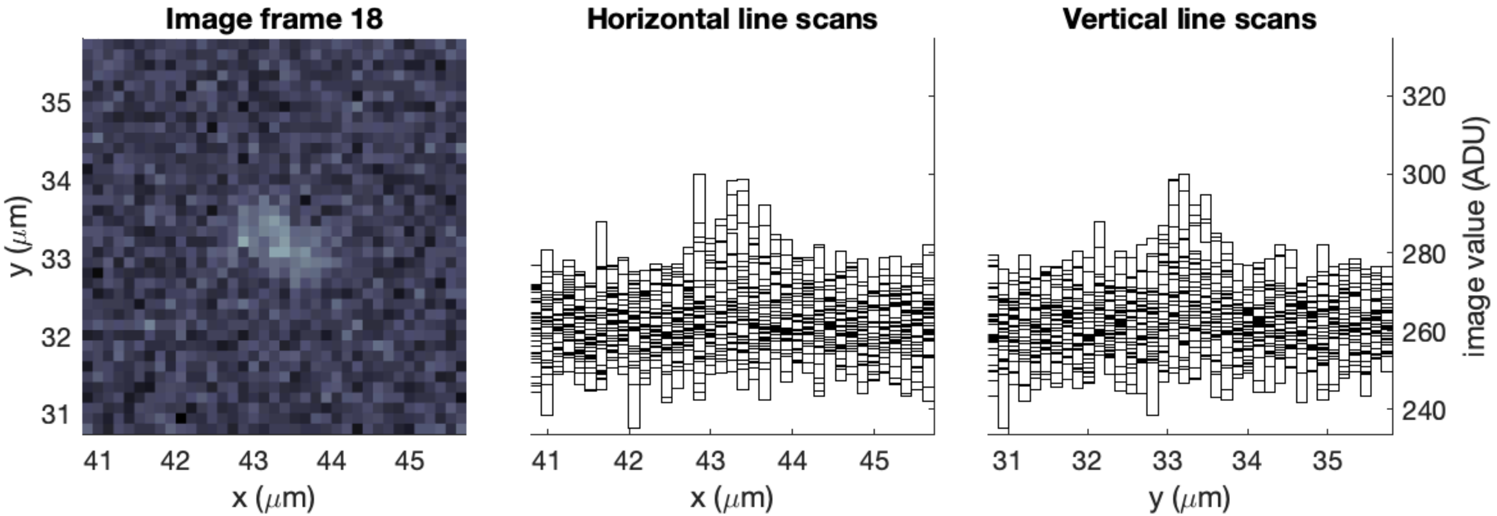

Image frame 18
Horizontal line scans
Vertical line scans
35
34
33
32
31
y (μm)
41
42
43
44
45
x (μm)
41
42
43
44
45
x (μm)
31
32
33
34
35
y (μm)
320
300
280
260
240
image value (ADU)

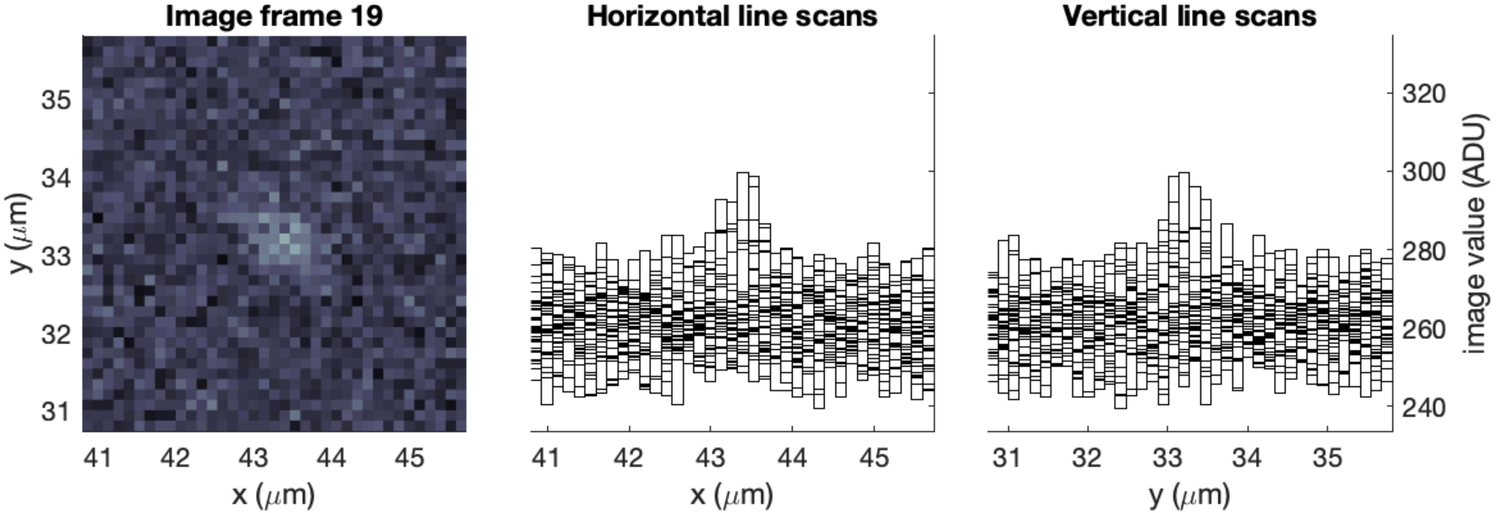

Image frame 19
Horizontal line scans
Vertical line scans
y (μm)
x (μm)
x (μm)
y (μm)
image value (ADU)
35
34
33
32
31
41
42
43
44
45
320
300
280
260
240

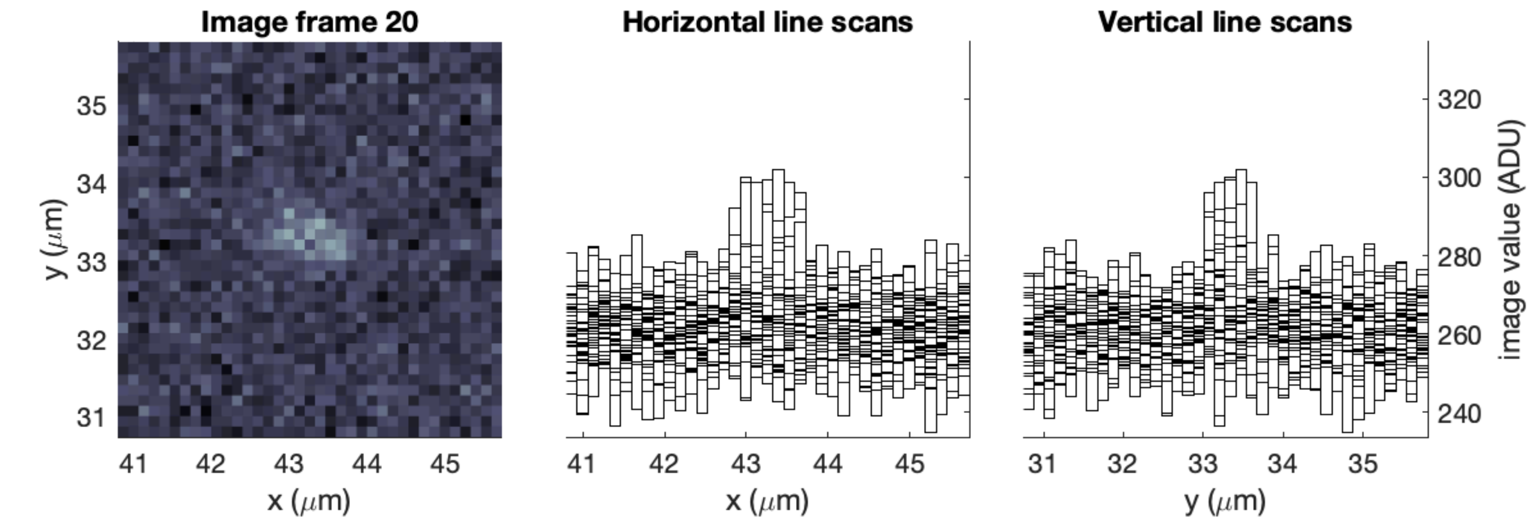
Image frame 20
Horizontal line scans
Vertical line scans
y (μm)
x (μm)
x (μm)
y (μm)
image value (ADU)
35
34
33
32
31
41
42
43
44
45
320
300
280
260
240

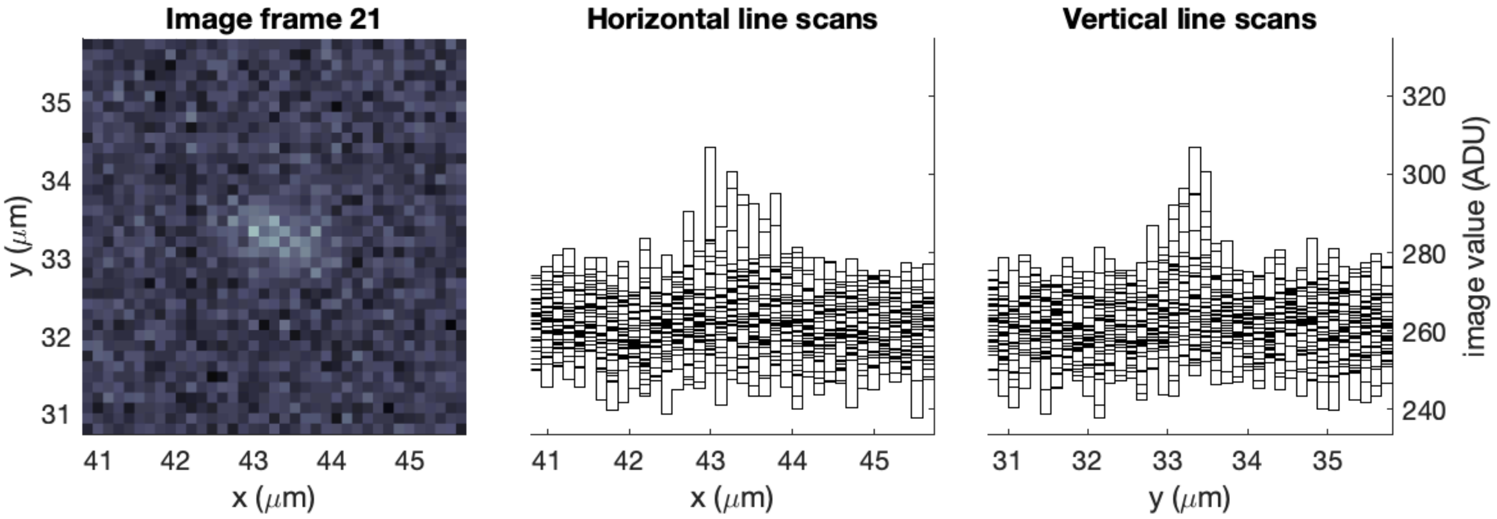
Image frame 21
Horizontal line scans
Vertical line scans
y (μm)
x (μm)
x (μm)
y (μm)
image value (ADU)
35
34
33
32
31
41
42
43
44
45
31
32
33
34
35
320
300
280
260
240

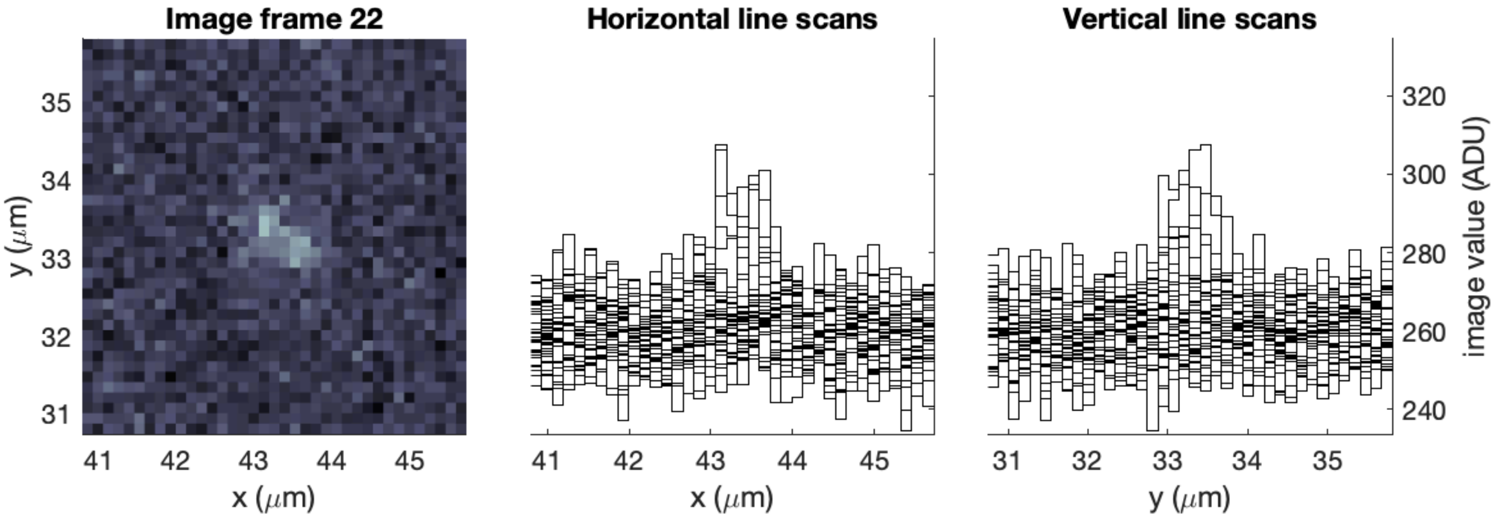

Image frame 22
Horizontal line scans
Vertical line scans
y (μm)
35
34
33
32
31
41
42
43
44
45
x (μm)
41
42
43
44
45
x (μm)
31
32
33
34
35
y (μm)
320
300
280
260
240
image value (ADU)
