# Supplementary figures and images for: BNP-Track: a framework for superresolved tracking
Source: Nat Methods. 2024 Jul 22;21(9):1716–24. doi: 10.1038/s41592-024-02349-9 (PMC11399105; doi:10.1038/s41592-024-02349-9)

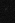

Supplement: Supplementary file 6 — Raw synthetic image stacks. [file 41592_2024_2349_MOESM6_ESM.tiff]
